# Osteosarcopenia, osteoarthritis and frailty: a two-sample Mendelian randomization study

**DOI:** 10.1007/s40520-025-03012-9

**Published:** 2025-04-21

**Authors:** Jili Liu, Xin Xia, Zhaolin Wang, Yanqin Wang, Gang Qin

**Affiliations:** 1 Department of Geriatrics, The First Hospital, Shanxi Medical University, Taiyuan, Shanxi Province 030001 China; 2 The Center of Gerontology and Geriatrics and National Clinical Research Center for Geriatrics, West China Hospital, Sichuan University, Chengdu, Sichuan Province 610041 China; 3 Department of Traditional Chinese Medicine, The Second Hospital, Shanxi Medical University, Taiyuan, Shanxi Province 030001 China; 4 Department of Hematology, Shanxi Hospital of Traditional Chinese Medicine, Taiyuan, Shanxi Province 030012 China

**Keywords:** Osteosarcopenia, Osteoarthritis, Frailty, Mendelian randomization

## Abstract

**Background:**

Musculoskeletal disease, which has a complicated relationship with frailty, is a common clinical problem among elderly individuals.

**Aims:**

This study evaluated the potential causal relationships between osteosarcopenia, osteoarthritis and frailty by Mendelian Randomization (MR) analysis.

**Methods:**

This study employed a two-sample MR approach to investigate the causal relationships among osteosarcopenia, osteoarthritis and frailty. Published summary statistics were used to obtain instrumental variables at the genome-wide significance level.

**Results:**

Among the age groups with osteoporosis, high total bone mineral density (TBMD) (45—60, OR = 0.966, 95% CI 0.940–0.993, *P* = 0.013) and TBMD (over 60, OR = 0.974, 95% CI 0.954–0.994, *P* = 0.011) reduced the risk of frailty. Similarly, high forearm BMD (FA-BMD), high ultradistal forearm BMD (UFA-BMD), and high Heel-BMD at different sites also reduced the risk of frailty (OR = 0.966, 95% CI 0.936–0.996, *P* = 0.028; OR = 0.975, 95% CI 0.953–0.997, *P* = 0.029; OR = 0.981, 95% CI 0.967–0.995, *P* = 0.008). Among the characteristics related to sarcopenia, grip strength in the left hand, grip strength in the right hand, appendicular lean mass, and walking pace were all protective factors for frailty (OR = 0.788, 95% CI 0.721–0.862, *P* < 0.001; OR = 0.800, 95% CI 0.737–0.869, *P* < 0.001; OR = 0.955, 95% CI 0.937–0.974, *P* = 0.000; OR = 0.480, 95% CI 0.388–0.593, *P* < 0.001), with low grip strength in those over 60 years of age significantly positively correlated with frailty (OR = 1.168, 95% CI 1.059–1.289, *P* = 0.002). The MR results of osteoarthritis and frailty revealed a causal relationship between specific joint sites and frailty, including KOA (OR = 1.086, 95% CI 1.017–1.160, *P* = 0.014), HOA (OR = 1.028, 95% CI 1.007–1.049, *P* = 0.009), and KOA/HOA (OR = 1.082, 95% CI 1.053–1.113, *P* = 0.000), increasing the risk of frailty.

**Conclusion:**

Osteosarcopenia, osteoarthritis and frailty exhibit significant causal effects, rendering them risk factors for frailty. Therefore, in clinical practice, patients with osteosarcopenia and osteoarthritis should be required to undergo relevant interventions to reduce the risk of frailty.

**Supplementary Information:**

The online version contains supplementary material available at 10.1007/s40520-025-03012-9.

## Background

Frailty is a geriatric multisystem syndrome characterized by decreased physiological reserves, increased vulnerability to stressors, a diminished ability to withstand insults, and decreased resilience following stress. It is a major contributor to functional decline and mortality in older adults. In a meta-analysis covering 62 countries and territories, the prevalence of frailty in community-dwelling participants ranged from 11–51% [1]. At the same time, statistics show that approximately 10% of community-dwelling older adults aged 60 years and above in China are frail, with the prevalence increasing with age, reaching approximately 15% in those aged 75–84 years and approximately 25% in those aged 85 years and above [2, 3, 4] and approximately 30% in hospitalized older adults [5]. The frailty index (FI) is a reliable instrument for identifying individuals at risk of frailty [6]. Frailty increases susceptibility to various adverse health outcomes and predicts an increased risk of falls, disability, hospitalization, and death [7]. Frailty is closely associated with OS, as frail individuals are more likely to exhibit decreased bone mass, reduced skeletal muscle mass, and lower muscle strength [8].

Osteosarcopenia (OS) was initially coined by Duque and colleagues [9] to describe a subset of older adults affected by both osteoporosis (OP)/osteopenia and sarcopenia (SP). Patients with OP/osteopenia exhibit decreased bone mineral density (BMD) and increased bone fragility, whereas those with SP experience a progressive decline in muscle mass, muscle strength and/or functional capacity. A meta-analysis of the global epidemiological characteristics and impact of OS revealed an overall prevalence rate of 18.5%, with the prevalence rate in the elderly (> 80 years old) subgroup being 24.8% [10]. Cell factors secreted by bone and skeletal muscle mediate interactions between these two tissues. Bone factors secreted by bone cells affect the maintenance of skeletal muscle homeostasis during periods of inactivity, activity, and exercise, whereas muscle factors produced, expressed and released by muscle fibres participate in regulating bone formation and resorption [11]. The coordinated action of bone and skeletal muscle maintains the normal function of the musculoskeletal system. Research has confirmed overlapping risk factors and pathogenic mechanisms between OP and SP [12], both of which increase the risk of frailty, falls, fractures, hospitalization, and death [13, 14, 15].

A large-scale cohort study in Korea revealed that knee osteoarthritis (OA) is associated with an increased risk of frailty [16]. Osteoarthritis (OA), a chronic degenerative joint disease, is characterized by joint pain and stiffness, leading to a decreased ability to withstand external pressure sources [17, 18]. Data from the EVKALIPT epidemiological study in Russia show that the prevalence of OA in people aged 65 years and above is as high as 57.6%, making it one of the major diseases affecting the duration and quality of active life and leading to loss of autonomy [19]. Other studies suggest that frailty may increase the risk of muscle atrophy, inflammation, and fall-related injuries, thus becoming a risk factor for the occurrence and progression of OA [20].

The above findings provide evidence that the relationships among OP, SP, OA, and frailty are complex, with interwoven influences on each other. Mendelian randomization (MR) provides a method to assess the causal relationship between exposure and outcome by avoiding unmeasured confounding factors and reverse causation. Genetic variations in instrumental variables (IVs) are randomly allocated at conception and are not influenced by disease progression [21]. In this study, two-sample MR analysis was conducted via IV from genome-wide association study (GWAS) datasets to explore the possible causal relationships between genetically predicted OS, OA, and frailty.

## Methods

### Study design

**Fig. 1:**
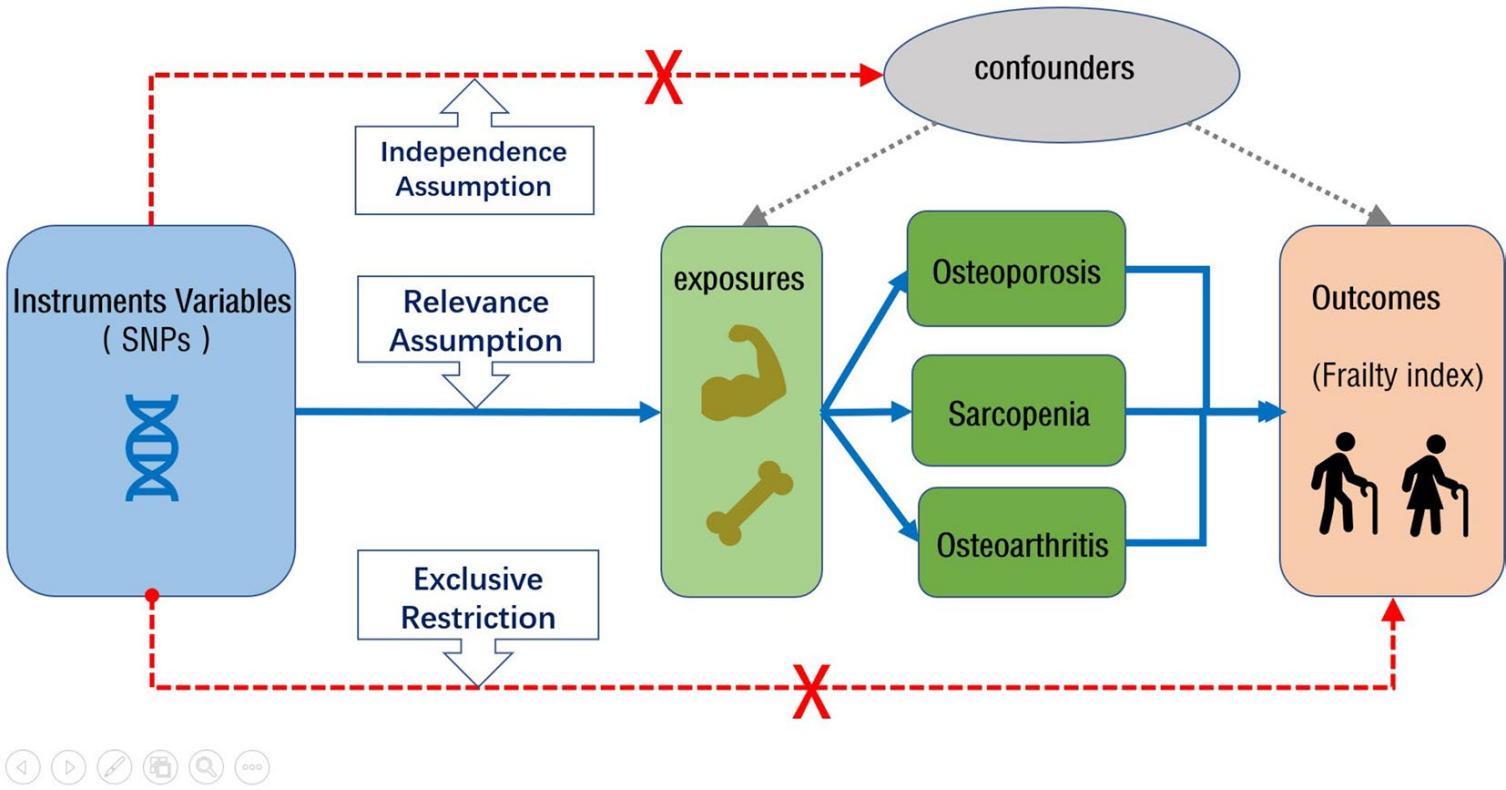
The flowchart of the two-sample MR study

A two-sample MR approach was employed to assess the potential causal effects between OS, OA, and frailty. Different age groups, specific site BMDs, characteristics related to SPs, specific joint OAs as exposures, and frailty index (FI) were used as outcomes. In MR analysis, only genetic variants that meet the following three stringent core assumptions are considered IVs: (1) the relevance assumption: IVs are highly correlated with the exposure factor; (2) the independence assumption: IVs are unrelated to confounding factors; and (3) the exclusion restriction assumption: IVs affect the outcome only through the exposure factor and not through any other pathways. The design flowchart is presented in Fig. 1.

### Data sources

#### BMD data sources

The BMD data consisted of phenotypes measured at three different age groups, total BMD (TBMD) and BMD measured at five different skeletal sites: TBMD (30–45 years), TBMD (45–60 years), TBMD (60 years and above), forearm BMD (FA-BMD), ultradistal forearm BMD (UFA-BMD), femoral neck BMD (FN-BMD), lumbar spine BMD (LS-BMD) and heel-BMD. The TBMD data for different age groups were derived from a meta-analysis of summary statistics from GWASs of whole-body BMD, comprising 30 epidemiological studies involving approximately 66,628 individuals from the United States, Europe and Australia, including those aged 30–45 years (*N* = 10,062), 45–60 years (*N* = 18,805), and 60 years and above (*N* = 22,504) [22]. Summary statistical data for FA-BMD (*N* = 8,143), FN-BMD (*N* = 32,735), and LS-BMD (*N* = 28,489) were obtained from the Genetic Factors for Osteoporosis (GeFOS) GWAS meta-analysis, which included a total of 53,236 individuals of European ancestry [23]. UFA-BMD data were sourced from the Nord-Trøndelag Health Study (HUNT) cohort (*N* = 21,907) [24], a population-based study in Nord-Trøndelag County, Norway. Heel-BMD data were obtained from the UK Biobank database (*N* = 583,314) of individuals of European ancestry [25]. In total, 86,302,665 SNPs were identified, with no overlap between samples.

#### Data sources for SP-related traits

SP-related traits include low grip strength, appendicular lean mass (ALM), and usual walking pace. Summary data for low grip strength were integrated from a meta-analysis of GWASs conducted in 22 independent cohorts [26] ^[24]^, including the UK Biobank, the Health and Retirement Study in the United States, and the Framingham Heart Study. The analysis involved a total of 256,523 individuals of European ancestry aged 60 years or older (48,596 cases and 207,927 controls) from the CHARGE consortium. Additionally, relevant data for grip strength, including 461,089 individuals for right hand grip strength and 461,026 individuals for left hand grip strength [27], were collected from the UK Biobank, all of which are of European ancestry. Summary statistical data for appendicular lean mass (ALM) were obtained from a GWAS involving 450,243 participants from the UK Biobank (244,730 females and 205,513 males) [28]. Summary statistical data for usual walking pace were derived from a GWAS dataset of 335,349 individuals of European ancestry from the UK Biobank (https://data.bris.ac.uk/data/dataset/pnoat8cxo0u52p6ynfaekeigi). In total, 47,111,667 SNPs were included in the analysis.

#### Data sources for OA

This research collected data from a meta-analysis of GWASs conducted with the UK Biobank and arcOGEN resources, involving up to 455,221 individuals of European descent (77,052 cases/378,169 controls) [29]. The dataset includes OA of the knee (KOA, 24,955 cases/378,169 controls), OA of the hip (HOA, 15,704 cases/378,169 controls), and knee and/or hip OA (KOA/HOA, 39,427 cases/378,169 controls), covering 90,036,274 SNPs.

Basic information regarding the enrolled characteristics is summarized in Table 1.

**Table 1:**
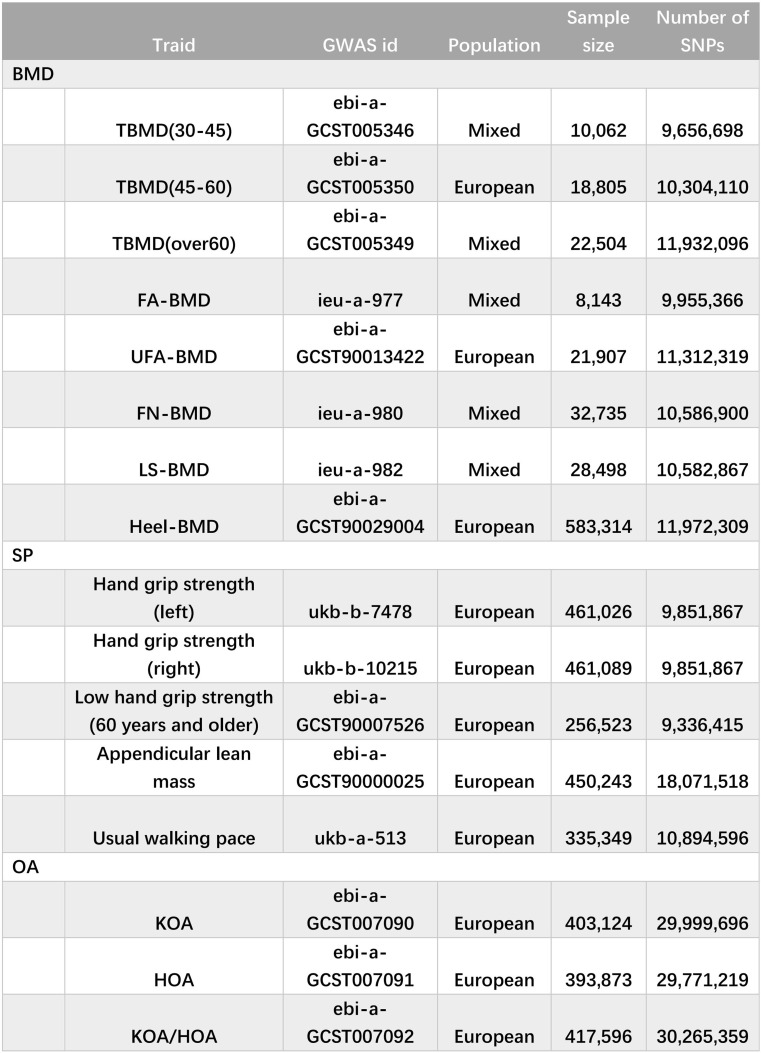
Details of the exposure and outcome data

### Selection of instrumental variables

This article extracted SNPs that showed significant associations (*p* < 5 × 10^-8) with exposure from the entire genome. Then, clumping within a 10,000 kb distance was performed to remove SNPs in linkage disequilibrium (r2 < 0.001) to ignore potential pleiotropic effects and ensure the independence of each SNP. This research used PhenoScanner to search for and exclude SNPs associated with the outcome to reduce potential confounding factors [30]. The R2 and F statistics (defined as the ratio of the mean square of the model to the mean square of the error) are then calculated, and weak IVs with F statistics lower than 10 are removed to mitigate potential bias introduced by weak IVs [31]. The detailed information of the included SNPs is summarized in Supplementary Table 1.

### MR analysis

Two-sample MR analysis was performed via R software (version 4.3.2) with the TwoSampleMR package (version 0.5.8). After selecting effective SNPs, the inverse variance weighting (IVW) method, which is considered the most reliable, was chosen as the primary method for MR analysis [32]. Additionally, supplementary analysis methods, including the weighted median estimator (WME), MR‒Egger regression, weighted mode, and simple mode, were used [33]. MR estimates are presented in the form of beta (β) values, odds ratios (ORs), and their corresponding 95% confidence intervals (CIs), with *P* < 0.05 indicating significant differences.

### Reliability assessment

To ensure the reliability and robustness of the results, this research conducted Cochran’s Q test, the MR‒Egger intercept test, funnel plot analysis, and leave-one-out sensitivity analysis [34]. Cochran’s Q test was used to assess potential heterogeneity, with *P* < 0.05 indicating statistically significant heterogeneity among SNPs. The MR‒Egger regression intercept test was performed to evaluate directional pleiotropy, where a lack of statistical significance compared with 0 suggests the absence of pleiotropy among SNPs [35]. Funnel plots were employed to visually inspect the symmetry of the effect estimate distributions. Finally, leave-one-out analysis was conducted to ensure the reliability of the associations with individual SNPs. Forest plots, scatter plots, leave‒one-out plots, and funnel plots were generated to assess the characteristics and effects of using individual SNPs in MR analysis.

## Results

### The causal effects of BMD on frailty index estimated by MR

**Fig. 2:**
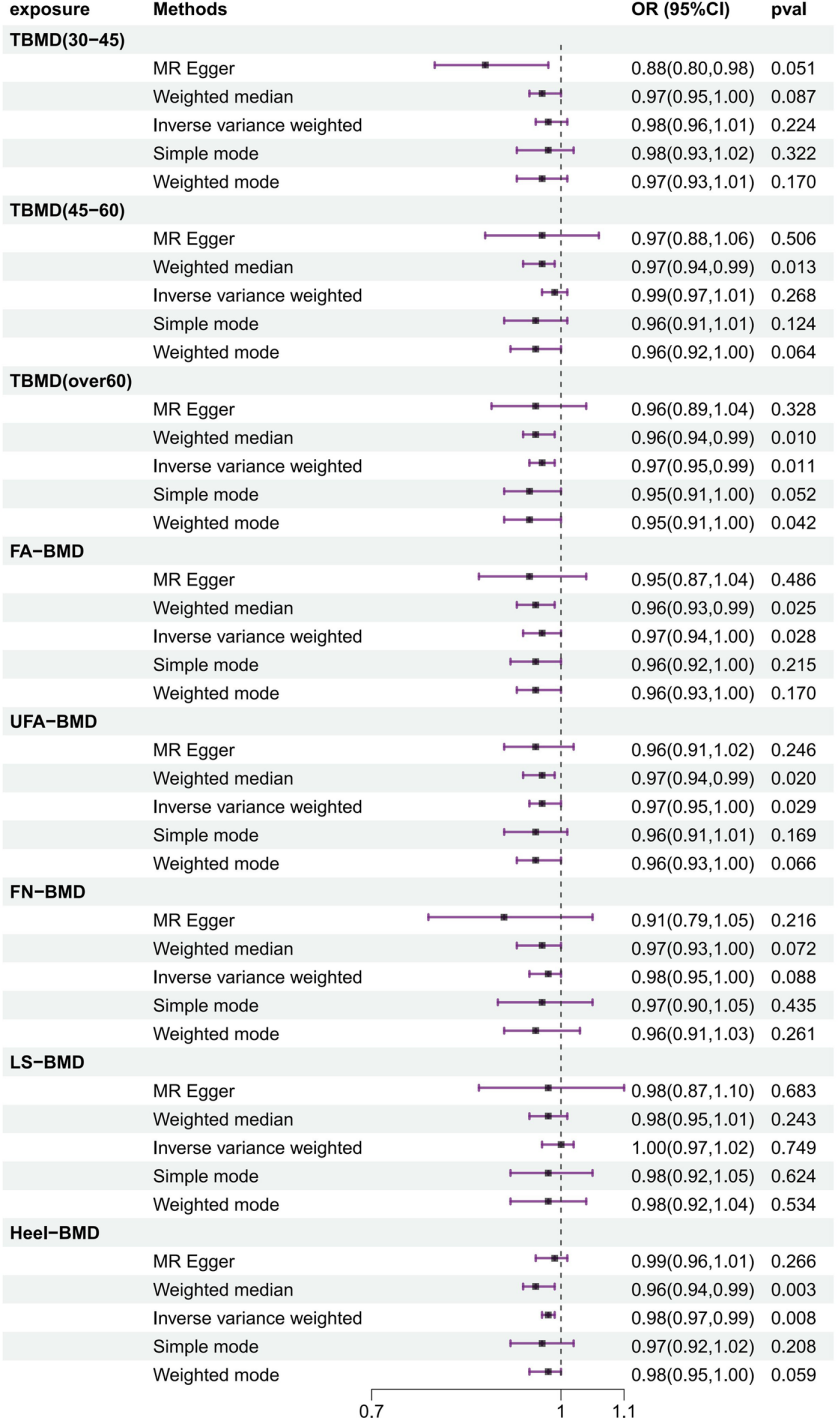
The results of MR analysis on OP-related traits and frailty

In the frailty MR analysis of BMD, the IVW method revealed a significant reduce in frailty risk for high TBMD (over 60), FA-BMD, UFA-BMD, and Heel-BMD (OR = 0.974, 95% CI 0.954–0.994, *P* = 0.011; OR = 0.966, 95% CI 0.936–0.996, *P* = 0.028; OR = 0.975, 95% CI 0.953–0.997, *P* = 0.029; OR = 0.981, 95% CI 0.967–0.995, *P* = 0.008), whereas the weighted median method also revealed an reduced frailty risk for high TBMD (45–60) (OR = 0.966, 95% CI 0.940–0.993, *P* = 0.013), with significant differences observed. No causal effects of TBMD (30–45), FN-BMD, or LS-BMD on frailty were found (Fig. 2). In subsequent sensitivity analyses, none of the exposure features showed heterogeneity. Phenotypic traits of OS, including TBMD (45–60), TBMD (over 60), FA-BMD, UFA-BMD, and Heel-BMD, presented no evidence of directional pleiotropy with a frailty index (intercepts = 0.002, *P* = 0.653; intercepts = 0.002, *P* = 0.564; intercepts = 0.002, *P* = 0.712; intercepts = 0.001, *P* = 0.601; intercepts = -0.0001, *P* = 0.724).

### The causal effects of SP-related traits on frailty index estimated by MR

**Fig. 3:**
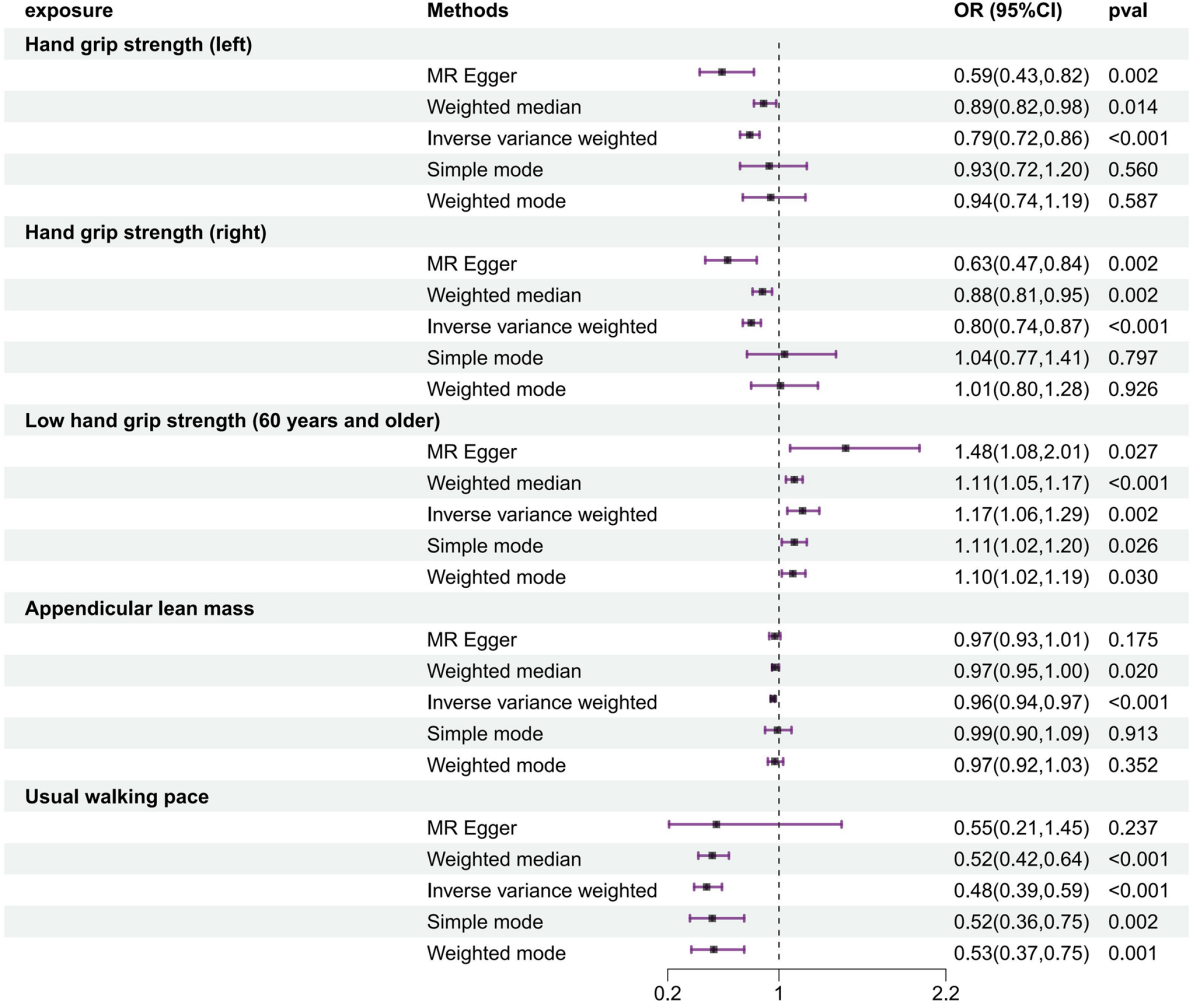
The results of MR analysis on SP-related traits and frailty

In the frailty causal analysis of SP, the IVW method yielded satisfactory results, indicating that increased grip strength in the left hand or right hand, increased appendicular lean mass, and fast walking pace reduced frailty risk (OR = 0.788, 95% CI 0.721–0.862, *P* = 0.000; OR = 0.800, 95% CI 0.737–0.869, *P* = 0.000; OR = 0.955, 95% CI 0.937–0.974, *P* = 0.000; OR = 0.480, 95% CI 0.388–0.593, *P* = 0.000) with low grip strength in individuals over 60 positively correlated with frailty (OR = 1.168, 95% CI 1.059–1.289, *P* = 0.002) (Fig. 3). SP characteristics, such as left grip strength (intercept = 0.003, *P* = 0.088), right grip strength (intercept = 0.003, *P* = 0.106), low grip strength in individuals over 60 (intercept = -0.015, *P* = 0.094), appendicular lean mass (intercept = -0.0003, *P* = 0.499), and usual walking pace (intercept = -0.001, *P* = 0.856), showed no potential pleiotropy.

### The causal effects of OA on frailty index estimated by MR

**Fig. 4:**
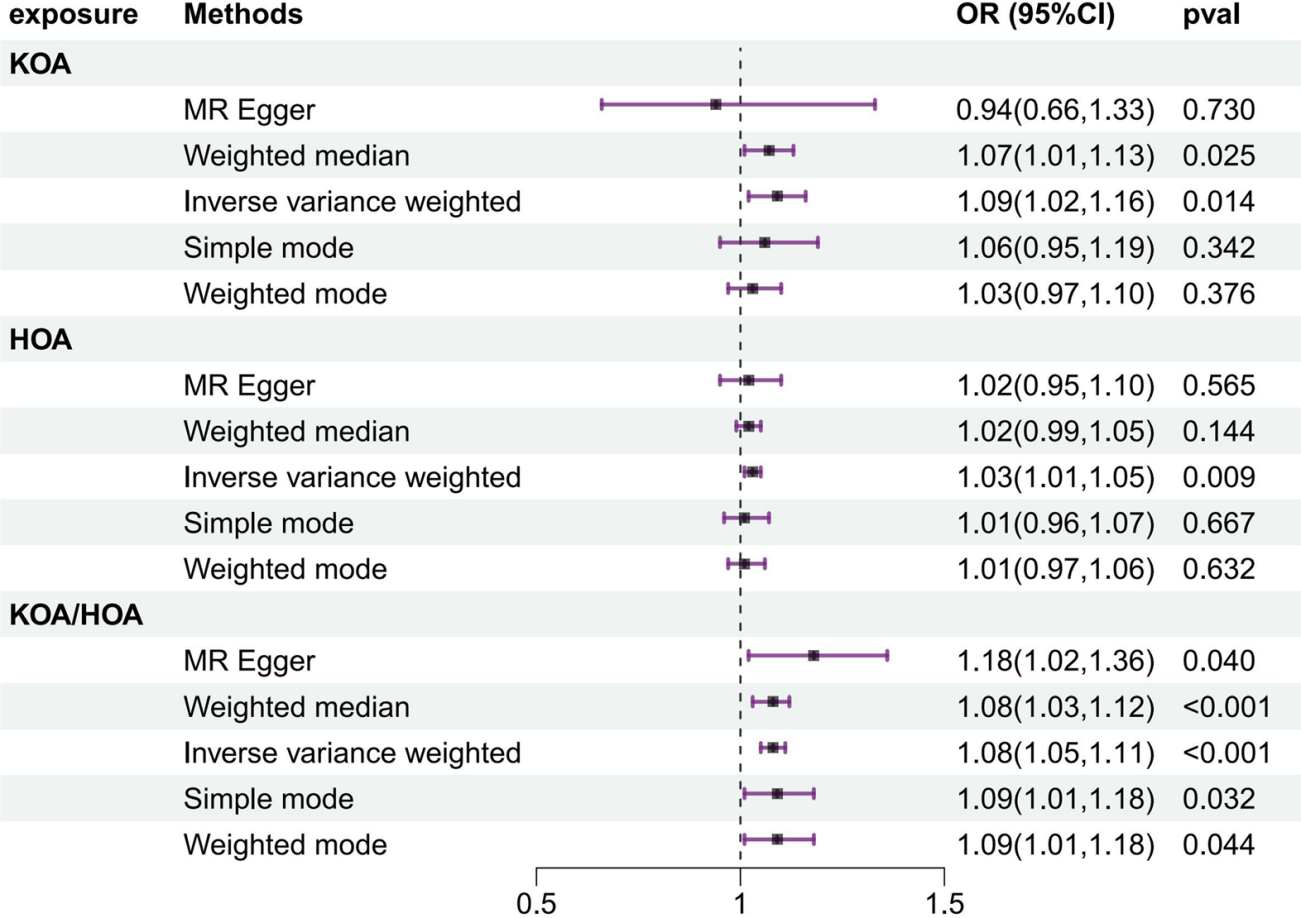
The results of MR analysis on OA-related traits and frailty

In the frailty MR analysis of OA, the IVW method revealed a significant positive correlation between specific joint sites and frailty, including KOA (OR = 1.086, 95% CI 1.017–1.160, *P* = 0.014), HOA (OR = 1.028, 95% CI 1.007–1.049, *P* = 0.009) and KOA/HOA (OR = 1.082, 95% CI 1.053–1.113, *P* = 0.000), indicating an increased likelihood of frailty (Fig. 4). Similarly, no pleiotropy was detected between OA phenotypes, including KOA (intercept = 0.011, *P* = 0.322), HOA (intercept = -0.0001, *P* = 0.979), or KOA/HOA (intercept = -0.004, *P* = 0.261), and frailty.

Leave-one-out analysis demonstrated that the causal effects were not driven by a single instrumental variable, indicating the stability of the results. Scatter plots, forest plots, leave‒one-out plots, and funnel plots for all the SNPs can be found in the supplementary materials (Supplementary Figures SI1-SI12).

## Discussion

Previous epidemiological and observational studies not only elucidated the associations and bidirectional relationships between musculoskeletal diseases and frailty but also identified partial associations among musculoskeletal diseases themselves. These intertwined relationships increase the complexity of these diseases, potentially introducing bias into the evidence due to the possibility of mutual confounding factors and making establishing causality difficult. In this study, extensive GWAS summary statistics were utilized to conduct Mendelian randomization analysis on the relationships between genetically predicted OS, OA and frailty, thereby avoiding potential confounding factors and reverse causation and comprehensively evaluating possible causal effects at the same time. The results successfully yielded causal conclusions, showing significant positive correlations between OS, OA and frailty, thereby increasing the risk of frailty.

OP/osteopenia is a common metabolic bone disease among middle-aged and elderly individuals, and the World Health Organization recommends bone mineral density (BMD) measured by dual-energy X-ray absorptiometry (DXA) as the diagnostic criterion for OS [36]. In this study, BMD was used as the exposure factor in the discussion of the relationship. Considering the close relationship between diseases and age, with the involvement of specific site BMD, TBMD was added to three age groups, namely, 30–45, 45–60, and over 60 years, as the objective of this study. The results were quite clear: the IVW method revealed that TBMD (over 60), FA-BMD, UFA-BMD, and Heel-BMD significantly increased frailty risk (OR = 0.974, 95% CI 0.954–0.994, *P* = 0.011; OR = 0.966, 95% CI 0.936–0.996, *P* = 0.028; OR = 0.975, 95% CI 0.953–0.997, *P* = 0.029; OR = 0.981, 95% CI 0.967–0.995, *P* = 0.008), and the results were highly consistent with those obtained via the weighted median method. The weighted median method revealed a negative correlation between TBMD (45–60) and frailty, indicating that low TBMD in the 45–60 age group also increased frailty risk (OR = 0.966, 95% CI 0.940–0.993, *P* = 0.013). These findings demonstrate a potential causal relationship between low BMD and frailty, regardless of the specific site, including the forearm, forearm ultradistal (wrist), or heel. Moreover, across different age groups, especially individuals over 45 years old, decreased TBMD similarly increased frailty risk, serving as an effective predictor of frailty. However, no causal relationships were found between TBMD (30–45), FN-BMD, LS-BMD, and frailty. Previous observational studies have yielded conflicting results. For example, one study involving 392 community-dwelling men reported an association between frailty severity and lower FN-BMD, but this association was not independent of age [37]. Another study of 3231 elderly European men revealed that severe frailty was associated with lower LS-BMD but not lower FN-BMD [38]. Notably, degenerative changes in the spinal region of elderly individuals can lead to inaccuracies in BMD readings, affecting the reliability of the results. This study avoided potential confounding factors and genetically demonstrated that decreased BMD is a contributing factor to frailty. This finding is consistent with recent similar studies [39], but this study added age stratification, making the design more comprehensive and providing genetic evidence that increasing BMD in individuals over 45 years of age reduces the risk of frailty.

SP is an important component of frailty, and both exhibit partial overlap in clinical presentation, primarily focusing on declines in physical function and diminished quality of life in the elderly population. A cross-sectional study involving 835 elderly inpatients and outpatients in Vietnam revealed a significant positive correlation between SP and increasing frailty severity after adjusting for sociodemographic and clinical factors [40]. Although SP and frailty have independent associations, they do exist as distinct geriatric syndromes in the elderly population. Identifying potential causal relationships between their interactions and correlations is essential. The results of this study successfully demonstrated the causal relationship between SP and frailty: decreased grip strength in the left and right hands, low grip strength in individuals over 60 years of age, increased frailty risk (OR = 0.788, 95% CI 0.721–0.862, *P* = 0.000; OR = 0.800, 95% CI 0.737–0.869, *P* = 0.000; OR = 1.168, 95% CI 1.059–1.289, *P* = 0.002), whereas increased ALM and faster walking pace significantly reduced frailty risk (OR = 0.955, 95% CI 0.937–0.974, *P* = 0.000; OR = 0.480, 95% CI 0.388–0.593, *P* = 0.000). However, a recent study analysing physical examination data from 393 residents aged 65 years and above in Sounkyo, Hokkaido, Japan, revealed no associations between grip strength or ALM and frailty or prefrailty after adjusting for age, sex, the serum ALB concentration, and medical history [41]. The generation of negative results might be due to the relatively small sample size of this study, with only a few frail individuals (9 males and 15 females), most of whom were participants in health check-ups, potentially introducing selection bias and the possibility of underestimating the results. In contrast, this study utilized large-scale summary data from GWAS databases, ensuring significant and reliable results.

In the causal association analysis between OA and frailty, the results are equally promising. The IVW method revealed a significant causal relationship between specific sites of OA and frailty, including KOA (OR = 1.086, 95% CI 1.017–1.160, *P* = 0.014), HOA (OR = 1.028, 95% CI 1.007–1.049, *P* = 0.009), and KOA/HOA (OR = 1.082, 95% CI 1.053–1.113, *P* = 0.000), increasing the likelihood of frailty. OA patients often reduce physical activity due to joint pain and restricted movement, leading to muscle atrophy and decreased physical fitness, exacerbating the state of frailty [42]. An observational study involving 1775 osteoarthritis patients revealed that individuals with osteoarthritis and pain were more likely to develop frailty than were those without pain or osteoarthritis, and elderly individuals with lower limb osteoarthritis pain had an even greater risk [43]. Similarly, a cohort study from the United States revealed that elderly individuals with knee pain in the general population had a 1.89-fold greater risk of developing frailty over the next 4 years, with even greater risks for those with bilateral knee pain [44]. This study yielded conclusions consistent with previous observational studies to provide evidence for early intervention in OA to reduce the risk of frailty, indicating a causal effect between OA coexisting in the knee, hip, and knee‒hip joints and frailty. Low-grade inflammation is common in patients with osteoarthritis (OA), and several pro-inflammatory markers, such as interleukin-6 (IL-6) and C-reactive protein (CRP), have been identified as independent predictors of the development of osteoarthritis [45, 46]. These inflammatory factors have also been linked to frailty [47, 48]. Inflammation may be the pathway that connects osteoarthritis (OA) with frailty.

OS, the coexistence of OP/osteopenia and SP, involves bones and muscles that are anatomically adjacent. They can mutually release mechanical signals to coordinate bone density and muscle mass and secrete biochemical factors such as myostatin, irisin, osteocalcin, and osteoprotegerin to regulate each other [49]. Basic research has investigated similar biological mechanisms between OS and OA simultaneously. Increased proinflammatory cytokines cause an imbalance in protein synthesis and degradation in muscles and cartilage, ultimately leading to muscle loss and cartilage damage [50]. Additionally, the levels of irisin in serum and synovial fluid are negatively correlated with the severity of OA [51]. In pathway studies, the Notch signalling pathway has been shown to extensively regulate cell proliferation, differentiation, and fate determination. Notch signalling activation is associated with degenerative musculoskeletal diseases (including intervertebral disc degeneration, OP, SP, and OA) [52]. These shared mechanisms and pathways are conducive to finding consistent treatment strategies for OS and OA, thereby providing direction and evidence for effectively reducing the risk of frailty occurrence and reversing the early progression of frailty.

The innovation of this study lies in the recognition of frailty as an early and reversible process. By preventing reversible risk factors and actively intervening at an early stage, the progression of frailty and disability in healthy elderly individuals can be delayed. OS and OA, as age-related musculoskeletal diseases, are closely associated with frailty. This study used genetic methods to establish causal relationships between OS, OA, and frailty. Interventions and treatments for OP, SP, and OA may facilitate the early reversal of frailty and potentially delay its progression. Limitations: We did not conduct reverse MR studies on frailty, OS, or OA, which may preclude the possibility of bidirectional causality. From the perspective of clinical intervention, multi-mode intervention targeting muscle and bone metabolism may provide clues and basis for reducing the occurrence of frailty.

## Conclusion

OS and OA are common musculoskeletal diseases in the elderly population. They coexist and intertwine with frailty all the time and increase the risk of adverse events. Mendelian randomization analysis provides genetic evidence supporting the causal relationships among OS, OA, and frailty. Decreased BMD, SP, and knee and hip OA significantly increase the risk of frailty. In clinical practice, patients with osteosarcopenia and osteoarthritis should be required to undergo relevant interventions to reduce the risk of frailty. Early identification, intervention, and treatment of these musculoskeletal diseases can effectively reduce the occurrence of frailty and delay its progression. Further refinements and supplements of the possible causal effects between frailty and musculoskeletal diseases will be provided in subsequent studies.

## Electronic supplementary material

Below is the link to the electronic supplementary material.

Supplementary Material 1

Supplementary Material 2

Supplementary Material 3

Supplementary Material 4

Supplementary Material 5

Supplementary Material 6

Supplementary Material 7

Supplementary Material 8

Supplementary Material 9

Supplementary Material 10

Supplementary Material 11

Supplementary Material 12

Supplementary Material 13

Supplementary Material 14

Supplementary Material 15

Supplementary Material 16

Supplementary Material 17

## Data Availability

No datasets were generated or analysed during the current study.
