# Supplementary figures and images for: Osteosarcopenia, osteoarthritis and frailty: a two-sample Mendelian randomization study
Source: Aging Clin Exp Res. 2025 Apr 21;37(1):132. doi: 10.1007/s40520-025-03012-9 (PMC12011954; doi:10.1007/s40520-025-03012-9)

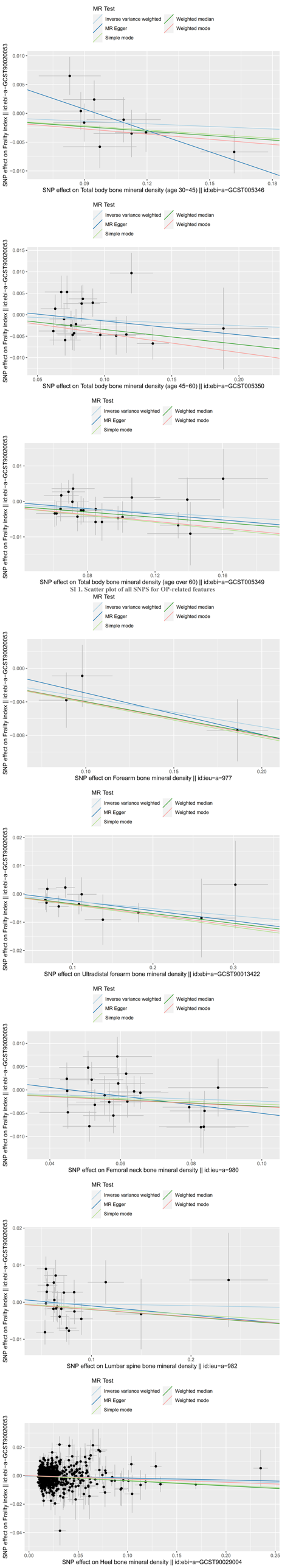

Supplement: Supplementary file 1 — Supplementary Material 1 [file 40520_2025_3012_MOESM1_ESM.tif]

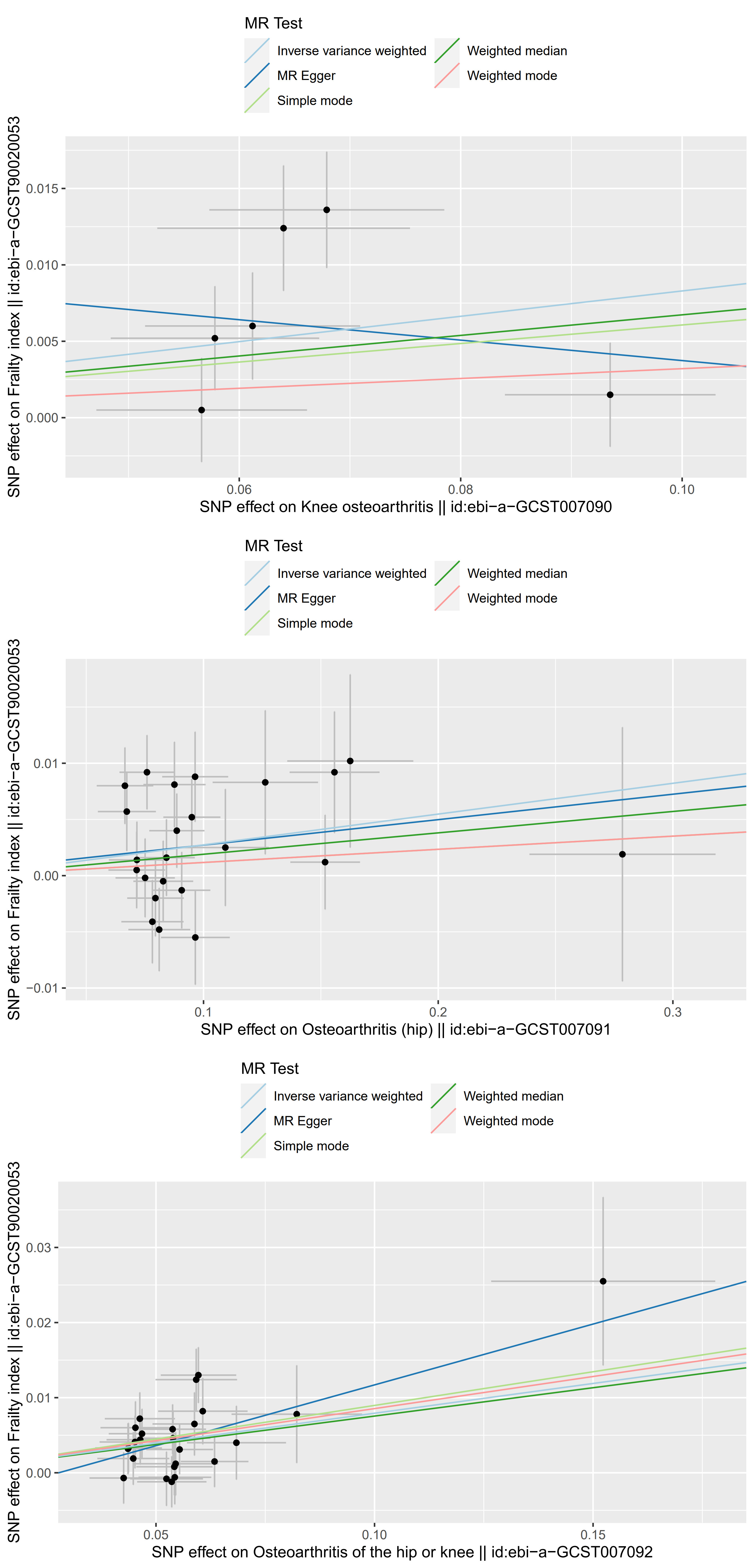

Supplement: Supplementary file 12 — Supplementary Material 12 [file 40520_2025_3012_MOESM12_ESM.tif]

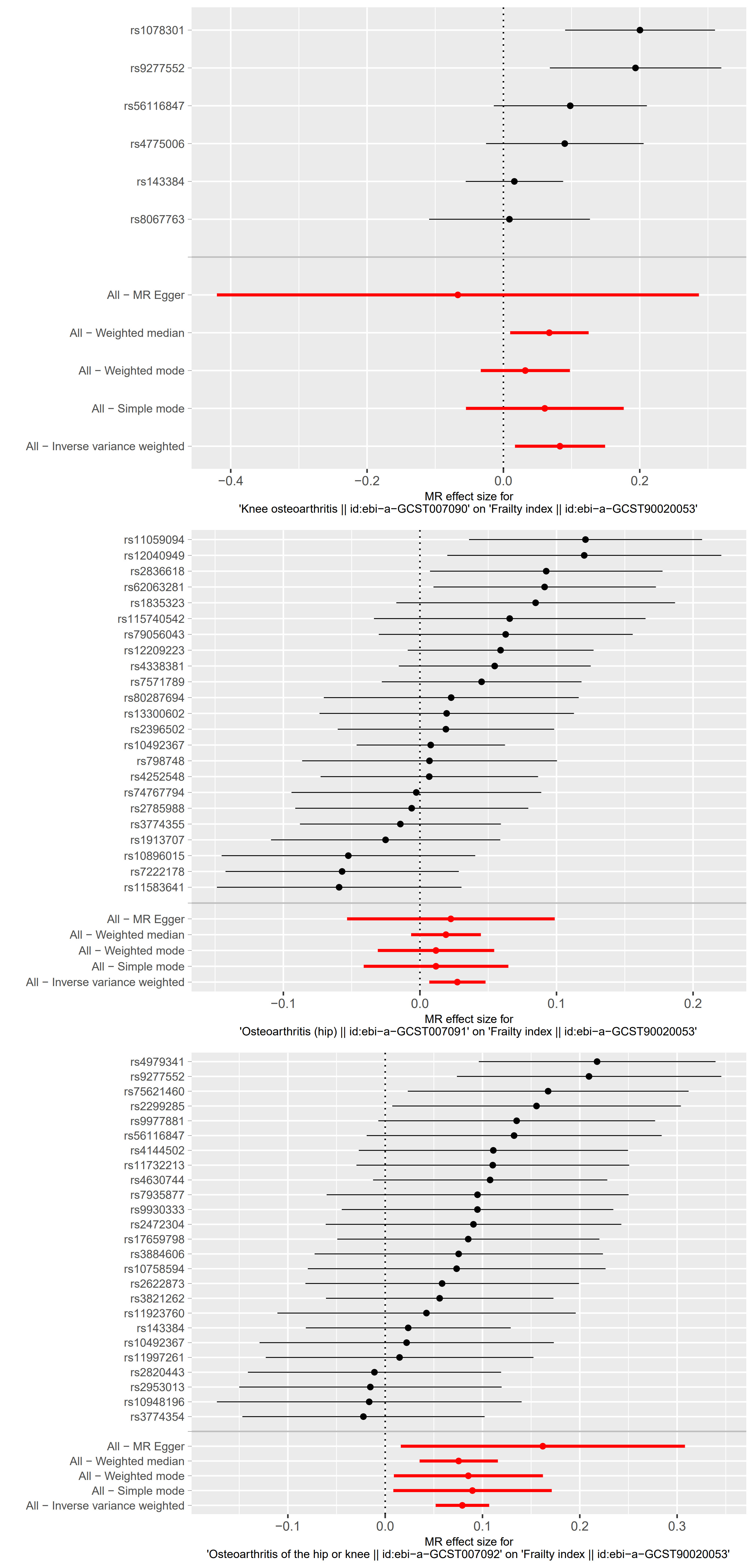

Supplement: Supplementary file 13 — Supplementary Material 13 [file 40520_2025_3012_MOESM13_ESM.tif]

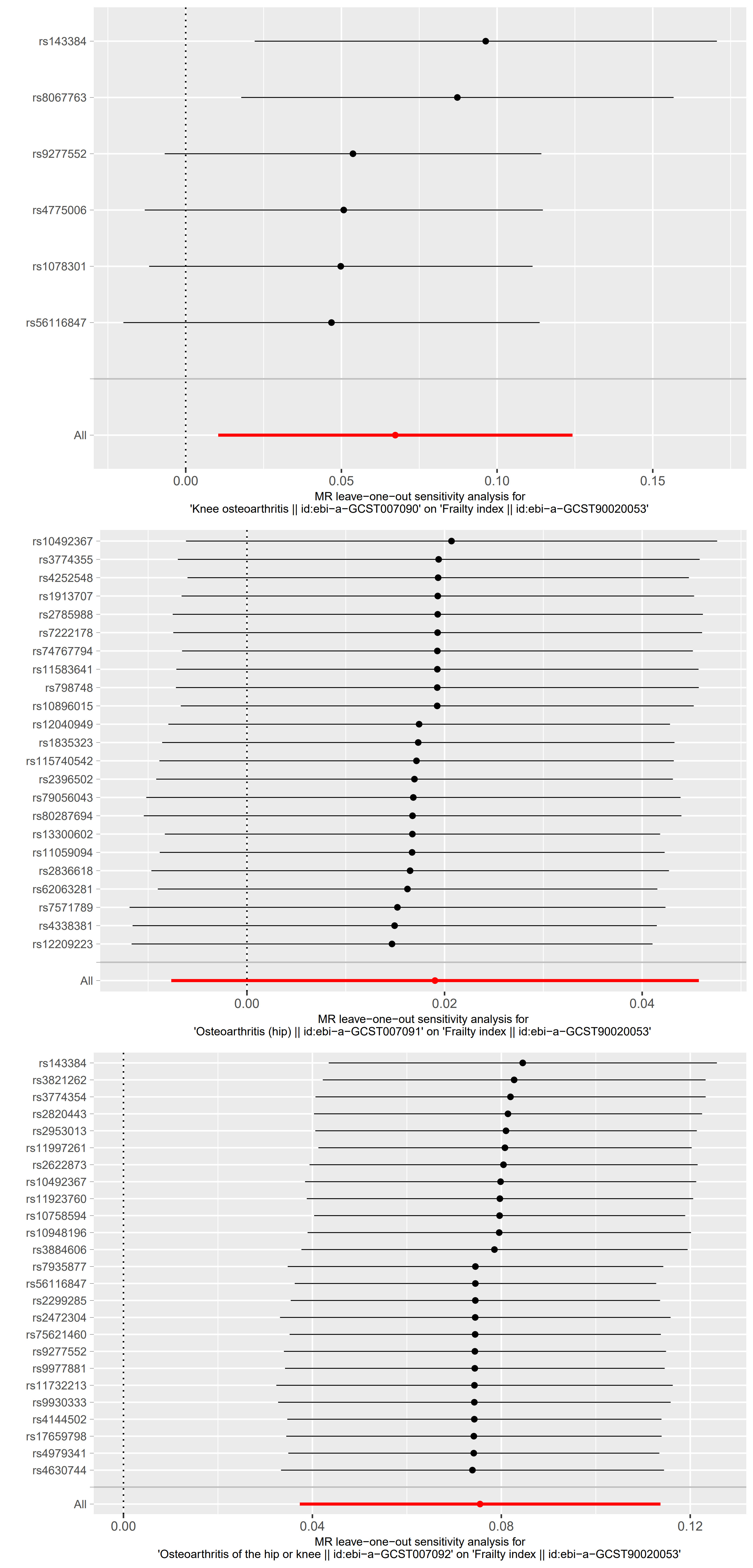

Supplement: Supplementary file 14 — Supplementary Material 14 [file 40520_2025_3012_MOESM14_ESM.tif]

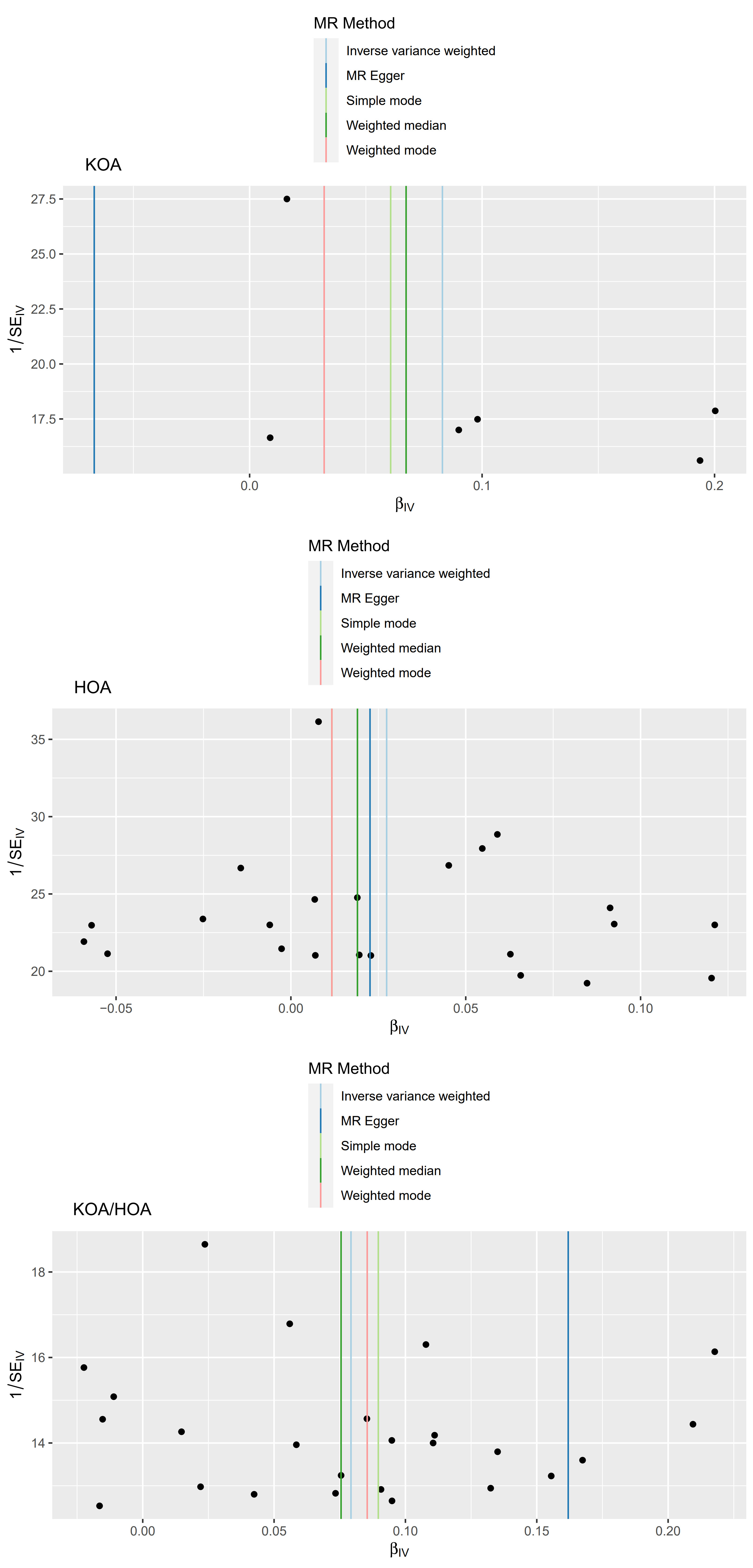

Supplement: Supplementary file 15 — Supplementary Material 15 [file 40520_2025_3012_MOESM15_ESM.tif]
