# Supplementary material for: Osteosarcopenia, osteoarthritis and frailty: a two-sample Mendelian randomization study
Source: Aging Clin Exp Res. 2025 Apr 21;37(1):132. doi: 10.1007/s40520-025-03012-9 (PMC12011954; doi:10.1007/s40520-025-03012-9)
Supplement: Supplementary file 16 — Supplementary Material 16 [file 40520_2025_3012_MOESM16_ESM.docx]

|  | SNP | Beta | Se | R | Fscore | Pvalue |
| --- | --- | --- | --- | --- | --- | --- |
| TBMD(30-45) |  |  |  |  |  |  |
|  | rs12742784 | 0.1126 | 0.0182 | 0.061566 | 38.27666 | 6.64E-10 |
|  | rs62259232 | 0.0899 | 0.0148 | 0.060451 | 36.89742 | 1.21E-09 |
|  | rs10005067 | -0.0883 | 0.0148 | -0.05938 | 35.59574 | 2.16E-09 |
|  | rs7761420 | 0.1195 | 0.0148 | 0.080243 | 65.19471 | 7.95E-16 |
|  | rs12534510 | 0.083 | 0.0148 | 0.055826 | 31.45088 | 2.2E-08 |
|  | rs3801387 | 0.1617 | 0.0162 | 0.099028 | 99.62997 | 1.42E-23 |
|  | rs55781332 | 0.1088 | 0.0177 | 0.061171 | 37.78429 | 6.98E-10 |
|  | rs11228240 | -0.0974 | 0.0175 | -0.05541 | 30.97718 | 2.58E-08 |
|  | rs8001611 | 0.0947 | 0.0148 | 0.063666 | 40.9427 | 1.54E-10 |
| TBMD(45-60) |  |  |  |  |  |  |
|  | rs6679981 | 0.1162 | 0.0138 | 0.061291 | 70.90128 | 4.81E-17 |
|  | rs12410251 | -0.0829 | 0.0136 | -0.04441 | 37.1562 | 9.85E-10 |
|  | rs2566752 | 0.075 | 0.0111 | 0.049215 | 45.65376 | 1.42E-11 |
|  | rs1968294 | -0.0632 | 0.0108 | -0.04264 | 34.24417 | 4.41E-09 |
|  | rs444561 | 0.0831 | 0.0107 | 0.056547 | 60.31627 | 1.02E-14 |
|  | rs6827815 | -0.12 | 0.016 | -0.05461 | 56.25 | 6.19E-14 |
|  | rs13130558 | -0.0698 | 0.0109 | -0.04665 | 41.00699 | 1.47E-10 |
|  | rs6894139 | -0.0618 | 0.0108 | -0.04169 | 32.74383 | 9.74E-09 |
|  | rs6557155 | 0.0968 | 0.011 | 0.064044 | 77.44 | 1.42E-18 |
|  | rs7740042 | -0.0773 | 0.0134 | -0.04203 | 33.2774 | 6.99E-09 |
|  | rs6465510 | 0.0787 | 0.0112 | 0.051177 | 49.37572 | 1.79E-12 |
|  | rs3801387 | 0.1359 | 0.0119 | 0.082996 | 130.4202 | 3.49E-30 |
|  | rs7010267 | 0.0838 | 0.0106 | 0.057558 | 62.49947 | 3.34E-15 |
|  | rs7105218 | -0.1086 | 0.0147 | -0.0538 | 54.57893 | 1.79E-13 |
|  | rs476597 | -0.0706 | 0.0115 | -0.04473 | 37.68892 | 8.33E-10 |
|  | rs118115924 | -0.3132 | 0.0506 | -0.04509 | 38.31267 | 6.1E-10 |
|  | rs7398996 | -0.0762 | 0.0113 | -0.04912 | 45.47294 | 1.56E-11 |
|  | rs9594738 | -0.0912 | 0.0106 | -0.06262 | 74.02492 | 9.95E-18 |
|  | rs78667121 | 0.1885 | 0.0332 | 0.04137 | 32.2364 | 1.3E-08 |
|  | rs884205 | 0.072 | 0.0126 | 0.041636 | 32.65306 | 9.97E-09 |
|  | rs6510186 | -0.0677 | 0.0122 | -0.04044 | 30.7934 | 3.11E-08 |
| TBMD(over60) |  |  |  |  |  |  |
|  | rs56104760 | -0.0715 | 0.0127 | -0.0375 | 31.69601 | 2E-08 |
|  | rs2566752 | 0.0776 | 0.0101 | 0.051152 | 59.03108 | 1.55E-14 |
|  | rs7548588 | 0.0617 | 0.0099 | 0.041511 | 38.84185 | 3.8E-10 |
|  | rs34920465 | 0.1008 | 0.0126 | 0.053255 | 64 | 9.41E-16 |
|  | rs55983207 | 0.1409 | 0.0255 | 0.03681 | 30.53104 | 3.37E-08 |
|  | rs2371447 | 0.0711 | 0.0099 | 0.047822 | 51.57851 | 6.58E-13 |
|  | rs6942191 | -0.0645 | 0.0103 | -0.04171 | 39.21435 | 4.23E-10 |
|  | rs6557155 | 0.0981 | 0.0102 | 0.063983 | 92.49913 | 5.18E-22 |
|  | rs1936792 | 0.0607 | 0.011 | 0.036761 | 30.45033 | 3.72E-08 |
|  | rs7740042 | -0.0741 | 0.0121 | -0.04079 | 37.50297 | 9.05E-10 |
|  | rs34670419 | -0.1603 | 0.0265 | -0.04029 | 36.59109 | 1.4E-09 |
|  | rs7787512 | 0.0849 | 0.0101 | 0.055949 | 70.65984 | 3.56E-17 |
|  | rs6978070 | 0.0641 | 0.0103 | 0.041451 | 38.72947 | 4.72E-10 |
|  | rs3801387 | 0.1337 | 0.0108 | 0.082248 | 153.2552 | 2.82E-35 |
|  | rs11995824 | -0.0806 | 0.0098 | -0.05475 | 67.64223 | 2.8E-16 |
|  | rs10824760 | 0.0886 | 0.0149 | 0.039609 | 35.35859 | 3.05E-09 |
|  | rs7131442 | 0.0762 | 0.0119 | 0.042648 | 41.00304 | 1.81E-10 |
|  | rs61884328 | 0.1063 | 0.0168 | 0.042143 | 40.03575 | 2.46E-10 |
|  | rs11228240 | -0.0848 | 0.0114 | -0.04953 | 55.33272 | 1.12E-13 |
|  | rs9533094 | -0.0688 | 0.0097 | -0.04723 | 50.30758 | 1.3E-12 |
|  | rs8073650 | -0.0958 | 0.0174 | -0.03668 | 30.31325 | 3.59E-08 |
|  | rs2741856 | 0.1391 | 0.0194 | 0.047744 | 51.41038 | 7.03E-13 |
| FA-TMD |  |  |  |  |  |  |
|  | rs13423976 | 0.097978 | 0.017179 | 0.054791 | 32.5283 | 2.3E-08 |
|  | rs6894139 | -0.08894 | 0.015638 | -0.05464 | 32.34466 | 2.52E-08 |
|  | rs7776725 | 0.186109 | 0.017421 | 0.102245 | 114.127 | 1.21E-25 |
| UFA-TMD |  |  |  |  |  |  |
|  | rs140902470 | 0.260415 | 0.043191 | 0.040705 | 36.35394 | 1.65E-09 |
|  | rs55983207 | 0.137643 | 0.022532 | 0.041239 | 37.31685 | 1E-09 |
|  | rs192701514 | -0.30262 | 0.040506 | -0.05041 | 55.81536 | 7.96E-14 |
|  | rs4505759 | 0.068504 | 0.011521 | 0.040143 | 35.35567 | 2.75E-09 |
|  | rs10656721 | 0.0699 | 0.010584 | 0.044579 | 43.61829 | 3.99E-11 |
|  | rs4869742 | -0.10786 | 0.011995 | -0.06064 | 80.85151 | 2.43E-19 |
|  | rs7741021 | 0.067504 | 0.010609 | 0.042952 | 40.48742 | 1.98E-10 |
|  | rs2707518 | 0.18182 | 0.011083 | 0.110171 | 269.1438 | 1.75E-60 |
|  | rs6973667 | 0.066054 | 0.011369 | 0.039227 | 33.75759 | 6.24E-09 |
|  | rs489247 | -0.08268 | 0.012172 | -0.04585 | 46.13943 | 1.1E-11 |
|  | rs2147161 | -0.09091 | 0.011785 | -0.05205 | 59.50904 | 1.22E-14 |
|  | rs76410205 | 0.111294 | 0.018303 | 0.041051 | 36.97623 | 1.2E-09 |
| FN-BMD |  |  |  |  |  |  |
|  | rs2566752 | 0.061943 | 0.007699 | 0.035963 | 64.73152 | 3.65E-15 |
|  | rs7524102 | 0.083798 | 0.009823 | 0.038128 | 72.77446 | 7.36E-17 |
|  | rs10170839 | -0.05936 | 0.007521 | -0.03528 | 62.28842 | 1.2E-14 |
|  | rs436448 | 0.063845 | 0.007565 | 0.037721 | 71.22557 | 1.56E-16 |
|  | rs1366594 | -0.07945 | 0.007525 | -0.04717 | 111.4827 | 5.44E-25 |
|  | rs10946458 | -0.04492 | 0.007974 | -0.02519 | 31.73134 | 3.63E-08 |
|  | rs13194508 | -0.05184 | 0.008915 | -0.026 | 33.81719 | 1.3E-08 |
|  | rs9478217 | 0.052736 | 0.007608 | 0.030989 | 48.04778 | 1.23E-11 |
|  | rs4448201 | 0.065631 | 0.007895 | 0.037156 | 69.10568 | 4.37E-16 |
|  | rs4281029 | 0.056816 | 0.009361 | 0.027137 | 36.83807 | 2.96E-09 |
|  | rs3779381 | 0.057989 | 0.008522 | 0.030421 | 46.3029 | 2.87E-11 |
|  | rs1485307 | -0.0616 | 0.00761 | -0.03618 | 65.51637 | 2.49E-15 |
|  | rs11024028 | 0.05552 | 0.009698 | 0.025598 | 32.77439 | 2.18E-08 |
|  | rs7108738 | 0.082771 | 0.009715 | 0.03808 | 72.589 | 8.07E-17 |
|  | rs1785493 | -0.04511 | 0.008037 | -0.0251 | 31.50202 | 4.06E-08 |
|  | rs4759320 | -0.04481 | 0.007933 | -0.02526 | 31.90904 | 3.33E-08 |
|  | rs71390846 | -0.05911 | 0.009756 | -0.02709 | 36.70452 | 3.16E-09 |
|  | rs10794639 | -0.05119 | 0.007546 | -0.03033 | 46.01535 | 3.3E-11 |
|  | rs7209460 | 0.050829 | 0.008199 | 0.027718 | 38.43274 | 1.35E-09 |
|  | rs11652763 | 0.083568 | 0.012659 | 0.029514 | 43.5794 | 1.09E-10 |
|  | rs2741856 | 0.087631 | 0.014131 | 0.027726 | 38.4565 | 1.34E-09 |
| LS-BMD |  |  |  |  |  |  |
|  | rs7524102 | 0.089822 | 0.011494 | 0.036925 | 61.06932 | 2.41E-14 |
|  | rs2566752 | 0.082922 | 0.008947 | 0.043781 | 85.89832 | 1.49E-19 |
|  | rs11680288 | -0.05424 | 0.008933 | -0.0287 | 36.86079 | 3.12E-09 |
|  | rs11692564 | 0.23782 | 0.039471 | 0.028477 | 36.30283 | 4.1E-09 |
|  | rs401680 | 0.056521 | 0.008802 | 0.030348 | 41.23414 | 3.7E-10 |
|  | rs1023940 | -0.06454 | 0.00876 | -0.03482 | 54.28292 | 6.47E-13 |
|  | rs1357651 | 0.068117 | 0.009151 | 0.035174 | 55.40818 | 3.75E-13 |
|  | rs6965122 | -0.06184 | 0.009266 | -0.03154 | 44.53322 | 7.4E-11 |
|  | rs35681117 | 0.055162 | 0.009645 | 0.027032 | 32.70962 | 2.39E-08 |
|  | rs7807953 | 0.075102 | 0.009698 | 0.036592 | 59.97063 | 4.11E-14 |
|  | rs2220189 | -0.08276 | 0.008797 | -0.04444 | 88.49725 | 4.25E-20 |
|  | rs11002249 | 0.069743 | 0.011142 | 0.029584 | 39.18095 | 1.01E-09 |
|  | rs11024028 | 0.065776 | 0.011325 | 0.027452 | 33.73327 | 1.44E-08 |
|  | rs2291467 | -0.07742 | 0.010148 | -0.03605 | 58.20451 | 9.64E-14 |
|  | rs894738 | -0.06278 | 0.009136 | -0.03248 | 47.225 | 2E-11 |
|  | rs78667121 | 0.149862 | 0.025668 | 0.027596 | 34.0878 | 1.21E-08 |
|  | rs9533094 | -0.08263 | 0.008741 | -0.04465 | 89.35992 | 2.8E-20 |
|  | rs73326583 | 0.072363 | 0.011887 | 0.028772 | 37.05857 | 2.83E-09 |
|  | rs71390846 | -0.06409 | 0.011373 | -0.02664 | 31.75832 | 3.8E-08 |
|  | rs9921222 | -0.05335 | 0.008789 | -0.02869 | 36.83906 | 3.16E-09 |
|  | rs884205 | 0.062204 | 0.010212 | 0.028789 | 37.10351 | 2.77E-09 |
|  | rs9749364 | 0.11438 | 0.018078 | 0.029903 | 40.03127 | 6.64E-10 |
|  | rs2235811 | -0.0543 | 0.009045 | -0.02837 | 36.04114 | 4.66E-09 |
|  | rs13046645 | -0.05586 | 0.009829 | -0.02686 | 32.30203 | 2.92E-08 |
| Heel-BMD |  |  |  |  |  |  |
|  | rs56104760 | -0.04346 | 0.002384 | -0.02386 | 332.2747 | 1.5E-81 |
|  | rs76183105 | -0.02031 | 0.003254 | -0.00817 | 38.95575 | 7.4E-13 |
|  | rs277402 | 0.012457 | 0.00219 | 0.007447 | 32.35487 | 1E-08 |
|  | rs3790608 | 0.040575 | 0.002595 | 0.020467 | 244.4611 | 1.3E-55 |
|  | rs1444436 | -0.01605 | 0.002006 | -0.01047 | 64.0075 | 1.9E-17 |
|  | rs6691053 | -0.0163 | 0.00226 | -0.00944 | 52.03687 | 8.7E-14 |
|  | rs80250119 | 0.012111 | 0.002236 | 0.00709 | 29.32678 | 4E-08 |
|  | rs77597677 | -0.01746 | 0.002612 | -0.00875 | 44.69223 | 4.9E-12 |
|  | rs80289296 | -0.03572 | 0.003454 | -0.01354 | 106.9603 | 2E-25 |
|  | rs6427847 | -0.01752 | 0.001903 | -0.01205 | 84.73756 | 3.2E-21 |
|  | rs2795315 | -0.03213 | 0.002443 | -0.01722 | 173.0148 | 1.6E-41 |
|  | rs1414660 | 0.084492 | 0.00235 | 0.04703 | 1293.065 | 1E-200 |
|  | rs7535617 | 0.019998 | 0.002318 | 0.011293 | 74.4004 | 2.3E-17 |
|  | rs3765971 | 0.029103 | 0.001953 | 0.019506 | 222.0239 | 4.4E-54 |
|  | rs12043801 | -0.01628 | 0.002369 | -0.009 | 47.20966 | 1.9E-13 |
|  | rs302686 | 0.011951 | 0.001965 | 0.007964 | 36.99497 | 8.7E-09 |
|  | rs80195615 | -0.02649 | 0.00395 | -0.00878 | 44.97504 | 2.1E-11 |
|  | rs2566752 | 0.035284 | 0.00192 | 0.024049 | 337.543 | 3.6E-77 |
|  | rs1325273 | -0.02168 | 0.002073 | -0.01369 | 109.4038 | 2.7E-27 |
|  | rs35492837 | 0.017294 | 0.001886 | 0.012005 | 84.07533 | 1.6E-20 |
|  | rs72692840 | -0.02796 | 0.003195 | -0.01146 | 76.60571 | 1.6E-19 |
|  | rs58688161 | 0.020558 | 0.00199 | 0.013525 | 106.73 | 2.9E-27 |
|  | rs12401678 | 0.01097 | 0.001875 | 0.007661 | 34.2401 | 7E-09 |
|  | rs7516171 | 0.027665 | 0.002428 | 0.014916 | 129.804 | 1.3E-31 |
|  | rs2879815 | 0.024437 | 0.005055 | 0.006329 | 23.36747 | 3.6E-08 |
|  | rs67631072 | 0.022033 | 0.001874 | 0.015393 | 138.2433 | 3.2E-32 |
|  | rs61780431 | -0.02094 | 0.002251 | -0.01218 | 86.53676 | 8.6E-23 |
|  | rs1766790 | -0.02701 | 0.002465 | -0.01435 | 120.0954 | 1.9E-28 |
|  | rs74487757 | -0.01519 | 0.002725 | -0.0073 | 31.04611 | 3.2E-09 |
|  | rs139603701 | -0.10468 | 0.007552 | -0.01815 | 192.1541 | 3.4E-46 |
|  | rs12124126 | -0.01944 | 0.001966 | -0.01295 | 97.85451 | 5.4E-25 |
|  | rs6540965 | 0.013939 | 0.001969 | 0.009269 | 50.12188 | 4.1E-15 |
|  | rs10917449 | -0.02049 | 0.002178 | -0.01231 | 88.43555 | 2.3E-21 |
|  | rs114268629 | -0.07219 | 0.005293 | -0.01786 | 186.0558 | 3.1E-45 |
|  | rs6684375 | 0.062694 | 0.002428 | 0.033783 | 666.4778 | 1.5E-155 |
|  | rs7546500 | 0.015753 | 0.001978 | 0.010428 | 63.43996 | 1.8E-15 |
|  | rs12127020 | 0.018078 | 0.002366 | 0.010003 | 58.37387 | 2.2E-15 |
|  | rs2566774 | -0.03766 | 0.002375 | -0.02076 | 251.5439 | 3.7E-64 |
|  | rs56732321 | -0.01486 | 0.002245 | -0.00867 | 43.80978 | 9.3E-13 |
|  | rs11576308 | 0.017244 | 0.001904 | 0.011861 | 82.07113 | 1.2E-18 |
|  | rs10737748 | -0.01344 | 0.002025 | -0.00869 | 44.06558 | 8.7E-11 |
|  | rs945508 | 0.012295 | 0.001857 | 0.008669 | 43.83788 | 1.2E-11 |
|  | rs12737669 | -0.02607 | 0.001884 | -0.01811 | 191.4277 | 4.9E-48 |
|  | rs10922299 | 0.015067 | 0.002059 | 0.009579 | 53.52516 | 8.2E-14 |
|  | rs11118447 | 0.022104 | 0.001996 | 0.014496 | 122.6017 | 6E-30 |
|  | rs7527300 | -0.02537 | 0.001893 | -0.01754 | 179.5657 | 5E-43 |
|  | rs10192149 | 0.011998 | 0.001854 | 0.008472 | 41.87484 | 2E-10 |
|  | rs13002567 | 0.019715 | 0.001961 | 0.013164 | 101.0995 | 1.4E-24 |
|  | rs56240884 | 0.020981 | 0.002036 | 0.013493 | 106.2151 | 5.2E-26 |
|  | rs4305309 | -0.07588 | 0.001938 | -0.05121 | 1533.849 | 1E-200 |
|  | rs11688492 | -0.01308 | 0.001859 | -0.00921 | 49.49737 | 7.2E-14 |
|  | rs34441013 | 0.03058 | 0.002074 | 0.019305 | 217.4697 | 6.2E-50 |
|  | rs10221698 | 0.015988 | 0.001866 | 0.011218 | 73.41729 | 7.2E-21 |
|  | rs6759927 | 0.019808 | 0.00199 | 0.013031 | 99.06256 | 6.4E-26 |
|  | rs10490046 | -0.02771 | 0.002241 | -0.01619 | 152.8534 | 2.5E-37 |
|  | rs7599234 | -0.01349 | 0.001853 | -0.00954 | 53.03817 | 3.3E-15 |
|  | rs35657711 | 0.052109 | 0.002259 | 0.030189 | 532.0862 | 1.1E-119 |
|  | rs2971887 | 0.052761 | 0.002082 | 0.03317 | 642.4853 | 2.8E-153 |
|  | rs7597759 | 0.011463 | 0.001949 | 0.007701 | 34.5992 | 3.3E-09 |
|  | rs6546334 | -0.01862 | 0.00195 | -0.0125 | 91.17859 | 1.8E-22 |
|  | rs10211539 | -0.00975 | 0.001874 | -0.00681 | 27.07801 | 1.1E-08 |
|  | rs713111 | 0.023716 | 0.002813 | 0.011038 | 71.08367 | 5.5E-18 |
|  | rs112900993 | 0.013623 | 0.002073 | 0.008603 | 43.17866 | 2E-10 |
|  | rs12622690 | -0.05029 | 0.00207 | -0.03179 | 590.1429 | 1.2E-140 |
|  | rs59243338 | -0.01256 | 0.002143 | -0.00768 | 34.38771 | 6.5E-09 |
|  | rs4664604 | 0.017471 | 0.002461 | 0.009296 | 50.40639 | 2.2E-13 |
|  | rs10191559 | -0.0237 | 0.00202 | -0.01536 | 137.6265 | 6.7E-33 |
|  | rs12477810 | 0.028053 | 0.00254 | 0.01446 | 121.9982 | 2.3E-29 |
|  | rs15380 | 0.016463 | 0.002294 | 0.009395 | 51.48759 | 6.4E-15 |
|  | rs62111830 | -0.01581 | 0.002109 | -0.00981 | 56.19187 | 6.1E-14 |
|  | rs55772556 | 0.014246 | 0.001975 | 0.009445 | 52.0445 | 5.4E-13 |
|  | rs10185019 | 0.014532 | 0.001957 | 0.00972 | 55.11664 | 2.4E-14 |
|  | rs11126989 | -0.01221 | 0.001864 | -0.00858 | 42.91365 | 1.3E-10 |
|  | rs10185316 | 0.016794 | 0.001972 | 0.011149 | 72.51154 | 1.2E-17 |
|  | rs2346204 | -0.01639 | 0.002136 | -0.01004 | 58.85757 | 3.8E-15 |
|  | rs67674876 | 0.021376 | 0.001927 | 0.014521 | 123.0248 | 4.9E-31 |
|  | rs72884306 | 0.015607 | 0.002375 | 0.008602 | 43.16853 | 4.8E-12 |
|  | rs11893248 | -0.0145 | 0.002383 | -0.00797 | 37.04411 | 6.1E-10 |
|  | rs10206992 | -0.02002 | 0.002125 | -0.01233 | 88.76177 | 9.3E-22 |
|  | rs114658808 | 0.062624 | 0.00606 | 0.01353 | 106.8086 | 5E-25 |
|  | rs150731260 | -0.04464 | 0.007806 | -0.00749 | 32.70491 | 5.7E-09 |
|  | rs6722557 | 0.019574 | 0.00213 | 0.012033 | 84.4763 | 1.7E-18 |
|  | rs12466877 | -0.02762 | 0.003751 | -0.00964 | 54.21352 | 1.8E-13 |
|  | rs12611565 | -0.01115 | 0.002031 | -0.00719 | 30.16122 | 2.1E-08 |
|  | rs6716216 | 0.036131 | 0.002826 | 0.016737 | 163.4442 | 5.9E-40 |
|  | rs4294980 | 0.017123 | 0.002261 | 0.009913 | 57.32815 | 1.6E-15 |
|  | rs78058190 | 0.037549 | 0.004764 | 0.01032 | 62.1303 | 6.7E-15 |
|  | rs73102769 | 0.019377 | 0.001864 | 0.013608 | 108.0337 | 4.4E-25 |
|  | rs1897468 | -0.01251 | 0.002039 | -0.00803 | 37.64811 | 1.1E-08 |
|  | rs13411485 | 0.022274 | 0.002788 | 0.010461 | 63.83882 | 4.8E-17 |
|  | rs665783 | -0.02016 | 0.002017 | -0.01309 | 99.93257 | 2.9E-24 |
|  | rs9631060 | -0.0204 | 0.002242 | -0.01192 | 82.82845 | 7.5E-21 |
|  | rs11688555 | 0.010895 | 0.002045 | 0.006974 | 28.37377 | 6.6E-09 |
|  | rs141795717 | 0.235186 | 0.007337 | 0.041931 | 1027.409 | 1E-200 |
|  | rs769079 | 0.010971 | 0.001895 | 0.007581 | 33.52788 | 8.8E-09 |
|  | rs76557027 | -0.02015 | 0.003294 | -0.00801 | 37.41973 | 2.4E-09 |
|  | rs6740159 | 0.020005 | 0.002803 | 0.009345 | 50.94791 | 7.7E-13 |
|  | rs10931982 | 0.055286 | 0.002215 | 0.032662 | 622.9307 | 4.5E-143 |
|  | rs10932006 | -0.0453 | 0.005473 | -0.01083 | 68.48303 | 1.8E-16 |
|  | rs35453391 | 0.013966 | 0.002134 | 0.008567 | 42.8156 | 1.6E-11 |
|  | rs7582828 | -0.01984 | 0.002163 | -0.01201 | 84.15427 | 4.1E-22 |
|  | rs9823123 | 0.01434 | 0.001892 | 0.009922 | 57.4346 | 2.1E-15 |
|  | rs13070996 | -0.01553 | 0.002085 | -0.00975 | 55.4638 | 6.4E-15 |
|  | rs2607748 | 0.010481 | 0.001885 | 0.007278 | 30.90081 | 2.6E-08 |
|  | rs1286662 | -0.02205 | 0.002412 | -0.01197 | 83.54084 | 2.6E-19 |
|  | rs150059308 | -0.07063 | 0.008676 | -0.01066 | 66.267 | 1.7E-16 |
|  | rs55786173 | -0.02294 | 0.004209 | -0.00714 | 29.69804 | 7.4E-09 |
|  | rs62259531 | 0.040921 | 0.003662 | 0.014631 | 124.8902 | 3.8E-31 |
|  | rs2306272 | 0.011791 | 0.002063 | 0.007483 | 32.66042 | 1.2E-08 |
|  | rs55814436 | 0.011838 | 0.002303 | 0.00673 | 26.42241 | 3.3E-08 |
|  | rs12487905 | 0.01358 | 0.002309 | 0.007701 | 34.59557 | 1.1E-08 |
|  | rs6794670 | -0.01514 | 0.002329 | -0.00851 | 42.28842 | 1.2E-09 |
|  | rs9861426 | -0.01192 | 0.001951 | -0.008 | 37.33573 | 1.8E-09 |
|  | rs7614560 | 0.017432 | 0.002273 | 0.01004 | 58.81021 | 1.9E-14 |
|  | rs346073 | -0.0278 | 0.002459 | -0.0148 | 127.8817 | 4.3E-31 |
|  | rs60682984 | 0.03596 | 0.005814 | 0.008097 | 38.24867 | 4.7E-10 |
|  | rs13065094 | 0.02181 | 0.001884 | 0.015153 | 133.9736 | 3.7E-35 |
|  | rs13088318 | 0.01599 | 0.001976 | 0.010593 | 65.46283 | 3E-16 |
|  | rs1991431 | -0.01979 | 0.001883 | -0.01376 | 110.4973 | 2E-27 |
|  | rs344081 | -0.04801 | 0.002843 | -0.02211 | 285.3025 | 4E-66 |
|  | rs10937104 | 0.011572 | 0.001992 | 0.007606 | 33.75068 | 8.3E-10 |
|  | rs370387 | 0.046919 | 0.001877 | 0.032707 | 624.6764 | 1.3E-148 |
|  | rs11915970 | 0.043646 | 0.002899 | 0.019712 | 226.7453 | 1.3E-52 |
|  | rs9829214 | -0.0137 | 0.001926 | -0.00931 | 50.59106 | 6.6E-13 |
|  | rs2465281 | 0.011782 | 0.001965 | 0.007849 | 35.93885 | 1.7E-10 |
|  | rs9864772 | -0.00927 | 0.001908 | -0.00636 | 23.60454 | 2E-08 |
|  | rs2819574 | -0.01188 | 0.002015 | -0.00772 | 34.7338 | 1.7E-09 |
|  | rs28373428 | 0.020318 | 0.002659 | 0.010005 | 58.39258 | 2.3E-15 |
|  | rs72871127 | -0.02785 | 0.00388 | -0.0094 | 51.52979 | 2.5E-13 |
|  | rs6549447 | 0.011803 | 0.002147 | 0.007197 | 30.21737 | 4.6E-08 |
|  | rs7637171 | 0.010572 | 0.00194 | 0.007136 | 29.70142 | 3.9E-08 |
|  | rs11709758 | -0.0197 | 0.002748 | -0.00938 | 51.37355 | 7.3E-12 |
|  | rs1482852 | -0.02933 | 0.001913 | -0.02007 | 235.1401 | 4.8E-57 |
|  | rs9290351 | 0.021406 | 0.002859 | 0.009804 | 56.07056 | 6.2E-15 |
|  | rs34782298 | 0.014937 | 0.002012 | 0.009718 | 55.09269 | 5.7E-14 |
|  | rs73207790 | -0.02304 | 0.002578 | -0.0117 | 79.83645 | 1.9E-20 |
|  | rs4694691 | 0.009229 | 0.001874 | 0.006449 | 24.26063 | 3.2E-08 |
|  | rs7699338 | -0.04654 | 0.00324 | -0.01881 | 206.3632 | 3.6E-48 |
|  | rs1386625 | -0.04561 | 0.003191 | -0.01871 | 204.3579 | 2.6E-51 |
|  | rs899631 | 0.019779 | 0.00192 | 0.013487 | 106.1287 | 1.1E-23 |
|  | rs13137483 | 0.01909 | 0.001937 | 0.012904 | 97.14689 | 1.8E-21 |
|  | rs66758084 | -0.02176 | 0.002459 | -0.01159 | 78.3509 | 4.9E-20 |
|  | rs56055826 | -0.02483 | 0.003962 | -0.00821 | 39.27407 | 5.2E-10 |
|  | rs3733424 | -0.0176 | 0.00189 | -0.01219 | 86.73107 | 2.3E-20 |
|  | rs1396190 | 0.013129 | 0.002054 | 0.00837 | 40.86992 | 1.5E-11 |
|  | rs7677709 | -0.01464 | 0.001899 | -0.01009 | 59.42818 | 9.2E-16 |
|  | rs79980676 | 0.036324 | 0.002999 | 0.015858 | 146.7177 | 5.8E-36 |
|  | rs2627696 | 0.020737 | 0.001876 | 0.014474 | 122.2256 | 3.4E-28 |
|  | rs12647210 | -0.01754 | 0.002607 | -0.00881 | 45.27796 | 3.2E-12 |
|  | rs6844904 | 0.030553 | 0.002499 | 0.016005 | 149.4648 | 6.1E-35 |
|  | rs13128681 | -0.01636 | 0.001954 | -0.01096 | 70.08491 | 3.9E-18 |
|  | rs6828759 | 0.012554 | 0.001912 | 0.008595 | 43.09365 | 1.4E-10 |
|  | rs13104911 | -0.01124 | 0.002011 | -0.00732 | 31.24415 | 1.8E-08 |
|  | rs327098 | 0.014187 | 0.002345 | 0.007922 | 36.6082 | 1.2E-09 |
|  | rs2702554 | 0.016155 | 0.001981 | 0.010677 | 66.49976 | 1.1E-16 |
|  | rs11729023 | 0.025931 | 0.002882 | 0.011782 | 80.98257 | 6.4E-21 |
|  | rs11940434 | -0.02325 | 0.002574 | -0.01182 | 81.54418 | 1.2E-19 |
|  | rs13108253 | -0.01808 | 0.001949 | -0.01215 | 86.10473 | 1.3E-21 |
|  | rs4505759 | 0.057987 | 0.002032 | 0.037341 | 814.4722 | 2.9E-195 |
|  | rs2174633 | 0.018575 | 0.002121 | 0.011464 | 76.67691 | 5.1E-19 |
|  | rs7694707 | -0.01369 | 0.001929 | -0.00929 | 50.35108 | 1.6E-12 |
|  | rs11934731 | -0.03682 | 0.002007 | -0.02402 | 336.6406 | 5.9E-78 |
|  | rs56389448 | 0.024356 | 0.002859 | 0.011154 | 72.58211 | 1E-18 |
|  | rs12503601 | 0.014668 | 0.001944 | 0.00988 | 56.94219 | 7.2E-16 |
|  | rs313146 | 0.015143 | 0.002 | 0.009911 | 57.30524 | 1.8E-15 |
|  | rs1550270 | 0.016399 | 0.002042 | 0.010516 | 64.51796 | 5.1E-15 |
|  | rs8180282 | 0.015291 | 0.002244 | 0.008923 | 46.45095 | 7.2E-12 |
|  | rs6866190 | -0.01821 | 0.00255 | -0.00935 | 50.99312 | 1.8E-12 |
|  | rs56083896 | -0.02356 | 0.003165 | -0.00975 | 55.42261 | 1.4E-13 |
|  | rs4957962 | 0.026436 | 0.004532 | 0.007637 | 34.01931 | 3.2E-08 |
|  | rs368510 | 0.025329 | 0.001981 | 0.016741 | 163.5189 | 2.1E-39 |
|  | rs4073717 | 0.013815 | 0.002332 | 0.007757 | 35.09813 | 6.8E-09 |
|  | rs7703857 | 0.018966 | 0.001908 | 0.013014 | 98.81069 | 1.5E-24 |
|  | rs55646464 | 0.011589 | 0.002039 | 0.007443 | 32.31217 | 9.8E-10 |
|  | rs7717596 | 0.01114 | 0.002022 | 0.007215 | 30.36637 | 1.2E-08 |
|  | rs4129645 | 0.019193 | 0.002091 | 0.012019 | 84.28192 | 5E-21 |
|  | rs10061435 | 0.032155 | 0.003179 | 0.013243 | 102.3211 | 5.3E-24 |
|  | rs7703751 | -0.02589 | 0.002138 | -0.01585 | 146.65 | 8.6E-34 |
|  | rs12332674 | 0.021654 | 0.0026 | 0.010902 | 69.34215 | 4.3E-18 |
|  | rs258758 | 0.015575 | 0.002404 | 0.008484 | 41.98654 | 3.1E-11 |
|  | rs1428968 | 0.023725 | 0.002434 | 0.012762 | 95.01824 | 4.1E-24 |
|  | rs13179493 | 0.030526 | 0.00208 | 0.019212 | 215.3736 | 2.2E-53 |
|  | rs116453584 | -0.0438 | 0.007291 | -0.00787 | 36.09025 | 3.8E-09 |
|  | rs35752015 | -0.01988 | 0.003041 | -0.00856 | 42.72533 | 2.2E-11 |
|  | rs7728907 | -0.01769 | 0.002213 | -0.01047 | 63.93292 | 3.4E-17 |
|  | rs10474093 | -0.0126 | 0.001895 | -0.00871 | 44.22987 | 7E-12 |
|  | rs437112 | 0.014912 | 0.001899 | 0.01028 | 61.64702 | 7.7E-16 |
|  | rs6885822 | -0.02372 | 0.002548 | -0.01219 | 86.66048 | 4.7E-22 |
|  | rs2546985 | -0.01207 | 0.002254 | -0.00701 | 28.66289 | 1.9E-08 |
|  | rs111251222 | -0.01383 | 0.002144 | -0.00845 | 41.61199 | 2.7E-11 |
|  | rs35151606 | -0.02182 | 0.003777 | -0.00756 | 33.36412 | 9.5E-10 |
|  | rs426975 | 0.013023 | 0.002227 | 0.007655 | 34.18733 | 5.2E-09 |
|  | rs62365472 | 0.032742 | 0.003122 | 0.013731 | 110.0026 | 2.7E-25 |
|  | rs2052480 | 0.027088 | 0.002071 | 0.017121 | 171.0315 | 4.2E-41 |
|  | rs6875585 | 0.014447 | 0.001995 | 0.009482 | 52.45087 | 9.7E-14 |
|  | rs10463643 | 0.014082 | 0.001961 | 0.009404 | 51.5875 | 8.4E-13 |
|  | rs4836373 | -0.01845 | 0.001874 | -0.01289 | 96.92226 | 2.3E-22 |
|  | rs6882422 | -0.03014 | 0.002928 | -0.01348 | 105.9346 | 6.6E-26 |
|  | rs4912661 | -0.01059 | 0.001881 | -0.00737 | 31.68846 | 1.7E-08 |
|  | rs7445576 | -0.01777 | 0.00231 | -0.01007 | 59.15828 | 1.8E-16 |
|  | rs10458143 | -0.02395 | 0.001854 | -0.01691 | 166.7623 | 7.9E-40 |
|  | rs3893538 | -0.02177 | 0.002752 | -0.01036 | 62.59683 | 2.9E-14 |
|  | rs9296151 | -0.04043 | 0.004958 | -0.01068 | 66.48151 | 2.6E-16 |
|  | rs1502201 | -0.0184 | 0.002131 | -0.0113 | 74.50377 | 1.6E-20 |
|  | rs150445982 | 0.093082 | 0.006503 | 0.018738 | 204.8693 | 1.9E-47 |
|  | rs530894 | 0.012254 | 0.001915 | 0.008379 | 40.95551 | 9E-11 |
|  | rs2764265 | -0.0108 | 0.001917 | -0.00738 | 31.75211 | 1.5E-08 |
|  | rs3941888 | 0.012393 | 0.002025 | 0.008014 | 37.46554 | 2.3E-11 |
|  | rs9482773 | 0.079608 | 0.001859 | 0.055976 | 1833.413 | 1E-200 |
|  | rs9340903 | -0.05241 | 0.005435 | -0.01262 | 92.98497 | 8.3E-22 |
|  | rs11752827 | 0.021266 | 0.003039 | 0.009163 | 48.97766 | 8.9E-12 |
|  | rs73029263 | -0.03222 | 0.002742 | -0.01538 | 138.0457 | 8.6E-35 |
|  | rs7765461 | -0.01363 | 0.002599 | -0.00687 | 27.50752 | 4.6E-09 |
|  | rs11758083 | 0.010372 | 0.00197 | 0.006895 | 27.72885 | 1.7E-08 |
|  | rs72868839 | 0.049135 | 0.003654 | 0.017604 | 180.83 | 2.8E-42 |
|  | rs852921 | 0.011913 | 0.002083 | 0.007487 | 32.69853 | 2.8E-09 |
|  | rs36009197 | 0.022018 | 0.00291 | 0.009906 | 57.24031 | 4.6E-15 |
|  | rs9385629 | -0.01422 | 0.002028 | -0.00918 | 49.1681 | 5.7E-14 |
|  | rs190038209 | -0.03512 | 0.006004 | -0.00766 | 34.2214 | 2.8E-09 |
|  | rs28585071 | -0.02512 | 0.002445 | -0.01345 | 105.4806 | 1.3E-25 |
|  | rs9503194 | -0.01347 | 0.001888 | -0.00934 | 50.85637 | 1.7E-12 |
|  | rs116228246 | 0.087145 | 0.006216 | 0.018352 | 196.5325 | 1E-46 |
|  | rs1155102 | 0.015544 | 0.002983 | 0.006822 | 27.1492 | 3.2E-08 |
|  | rs6940390 | 0.012343 | 0.001912 | 0.008453 | 41.6805 | 2.4E-10 |
|  | rs1977576 | 0.021595 | 0.00191 | 0.014803 | 127.8401 | 9.8E-28 |
|  | rs159408 | -0.01544 | 0.001895 | -0.01066 | 66.33291 | 3.4E-17 |
|  | rs17215781 | 0.027623 | 0.003531 | 0.010243 | 61.20838 | 5.5E-15 |
|  | rs9378485 | -0.01444 | 0.001908 | -0.00991 | 57.29865 | 1.6E-14 |
|  | rs9466056 | 0.012185 | 0.001906 | 0.008372 | 40.8844 | 1.3E-11 |
|  | rs578789 | 0.020046 | 0.002408 | 0.0109 | 69.31334 | 1.6E-17 |
|  | rs6904202 | -0.01159 | 0.002003 | -0.00757 | 33.46017 | 2.5E-09 |
|  | rs9359555 | -0.02213 | 0.002026 | -0.0143 | 119.367 | 5.6E-29 |
|  | rs1933801 | 0.015256 | 0.001985 | 0.010063 | 59.0744 | 8.7E-15 |
|  | rs1406667 | -0.02388 | 0.003124 | -0.01001 | 58.44474 | 3.2E-14 |
|  | rs4895959 | -0.05364 | 0.001864 | -0.03766 | 828.3604 | 1.1E-189 |
|  | rs553051 | 0.014206 | 0.002308 | 0.008058 | 37.88 | 6.4E-09 |
|  | rs41302867 | -0.02708 | 0.002824 | -0.01256 | 91.97281 | 1.4E-23 |
|  | rs74971894 | -0.03595 | 0.002905 | -0.0162 | 153.1241 | 2.3E-35 |
|  | rs75230517 | -0.10096 | 0.004227 | -0.03126 | 570.463 | 1.5E-133 |
|  | rs76813275 | -0.01874 | 0.003051 | -0.00804 | 37.7368 | 6.4E-10 |
|  | rs9442952 | -0.02446 | 0.001863 | -0.01719 | 172.3627 | 8.2E-43 |
|  | rs7774446 | 0.013694 | 0.002198 | 0.008157 | 38.81636 | 4.3E-10 |
|  | rs4869739 | -0.08394 | 0.002041 | -0.05377 | 1691.078 | 1E-200 |
|  | rs2941741 | 0.077477 | 0.00188 | 0.053874 | 1697.909 | 1E-200 |
|  | rs4870340 | 0.02483 | 0.002611 | 0.012449 | 90.42118 | 1.1E-22 |
|  | rs34220916 | 0.021962 | 0.00248 | 0.011595 | 78.43565 | 1.3E-17 |
|  | rs7806799 | -0.03627 | 0.003152 | -0.01506 | 132.3635 | 2E-31 |
|  | rs2732772 | 0.010463 | 0.001854 | 0.007389 | 31.85307 | 9.4E-10 |
|  | rs42038 | -0.02508 | 0.002018 | -0.01626 | 154.3453 | 3.3E-39 |
|  | rs6465495 | -0.01922 | 0.001937 | -0.01299 | 98.46348 | 6.9E-22 |
|  | rs140676008 | -0.04387 | 0.006707 | -0.00856 | 42.78618 | 4.5E-11 |
|  | rs6977460 | -0.01668 | 0.002108 | -0.01036 | 62.5849 | 3.7E-15 |
|  | rs73479996 | -0.02536 | 0.003667 | -0.00905 | 47.80776 | 2E-13 |
|  | rs2906146 | -0.01216 | 0.001973 | -0.00807 | 37.94807 | 3.1E-11 |
|  | rs7791093 | -0.02854 | 0.001879 | -0.01988 | 230.7087 | 1.1E-52 |
|  | rs1548607 | -0.01885 | 0.001966 | -0.01255 | 91.94383 | 4.9E-23 |
|  | rs2906193 | 0.017432 | 0.002238 | 0.010197 | 60.66276 | 5.9E-15 |
|  | rs12534970 | 0.010804 | 0.001849 | 0.007649 | 34.12721 | 4.5E-08 |
|  | rs3823871 | 0.02863 | 0.001946 | 0.019259 | 216.4466 | 4E-53 |
|  | rs87 | 0.036911 | 0.002318 | 0.020848 | 253.6354 | 8.3E-58 |
|  | rs10264106 | 0.04843 | 0.00237 | 0.026745 | 417.5305 | 3.3E-92 |
|  | rs212417 | -0.02901 | 0.001969 | -0.01929 | 217.207 | 1.4E-51 |
|  | rs71569053 | 0.097241 | 0.009091 | 0.014004 | 114.4165 | 8.5E-26 |
|  | rs150967545 | 0.057586 | 0.005495 | 0.013721 | 109.8398 | 2E-25 |
|  | rs148825265 | -0.04572 | 0.008368 | -0.00715 | 29.84536 | 5.9E-09 |
|  | rs798544 | 0.016133 | 0.002017 | 0.010471 | 63.9659 | 2.1E-17 |
|  | rs12673062 | 0.012808 | 0.002248 | 0.00746 | 32.46485 | 3.6E-09 |
|  | rs73083768 | 0.031334 | 0.002174 | 0.018865 | 207.6713 | 2.2E-50 |
|  | rs62449135 | -0.03859 | 0.004332 | -0.01166 | 79.35312 | 1E-18 |
|  | rs78941622 | 0.028672 | 0.005 | 0.007507 | 32.87762 | 2.4E-08 |
|  | rs74910854 | -0.0223 | 0.003651 | -0.008 | 37.3171 | 1.2E-10 |
|  | rs149078150 | 0.047372 | 0.0081 | 0.007657 | 34.20453 | 3.1E-10 |
|  | rs113488960 | 0.060227 | 0.007457 | 0.010574 | 65.22276 | 2.7E-17 |
|  | rs77847666 | 0.071438 | 0.004835 | 0.019343 | 218.3228 | 1.8E-51 |
|  | rs2707493 | 0.055705 | 0.006043 | 0.01207 | 84.9863 | 1.6E-21 |
|  | rs2908007 | 0.169344 | 0.001885 | 0.116821 | 8070.721 | 1E-200 |
|  | rs2303931 | -0.01982 | 0.002121 | -0.01224 | 87.3402 | 2.9E-21 |
|  | rs10244904 | -0.01788 | 0.002331 | -0.01004 | 58.8534 | 1.4E-14 |
|  | rs6973667 | 0.061803 | 0.001972 | 0.040995 | 981.9659 | 1E-200 |
|  | rs571356 | -0.01399 | 0.001984 | -0.00923 | 49.73468 | 9.3E-13 |
|  | rs6965122 | -0.04562 | 0.001964 | -0.0304 | 539.6529 | 2.2E-131 |
|  | rs7797740 | 0.014649 | 0.002664 | 0.007199 | 30.23567 | 2.8E-08 |
|  | rs117124857 | -0.078 | 0.006543 | -0.01561 | 142.1213 | 1.3E-34 |
|  | rs62462473 | 0.016033 | 0.002463 | 0.008523 | 42.37415 | 7.4E-11 |
|  | rs3098871 | -0.0285 | 0.001877 | -0.01988 | 230.5253 | 1.2E-55 |
|  | rs4304349 | -0.0109 | 0.001949 | -0.00733 | 31.3116 | 9.6E-09 |
|  | rs7823835 | -0.02246 | 0.002313 | -0.01271 | 94.29206 | 7.6E-25 |
|  | rs72656010 | 0.024894 | 0.002777 | 0.011737 | 80.36979 | 6.8E-19 |
|  | rs17679410 | 0.024591 | 0.002251 | 0.014303 | 119.3615 | 1.2E-27 |
|  | rs689411 | 0.014658 | 0.001961 | 0.009786 | 55.8646 | 1.9E-14 |
|  | rs2929308 | -0.03326 | 0.003514 | -0.01239 | 89.57125 | 1.8E-21 |
|  | rs569209 | -0.01231 | 0.002231 | -0.00722 | 30.42725 | 5.1E-09 |
|  | rs6471752 | -0.02446 | 0.002608 | -0.01228 | 87.93592 | 1.1E-22 |
|  | rs62515437 | -0.01333 | 0.002242 | -0.00778 | 35.34752 | 5.3E-09 |
|  | rs11785920 | -0.01604 | 0.00246 | -0.00853 | 42.49437 | 1.5E-11 |
|  | rs2581260 | 0.024736 | 0.002613 | 0.012395 | 89.63809 | 7.4E-23 |
|  | rs12545602 | 0.020912 | 0.002799 | 0.009783 | 55.82966 | 1.3E-15 |
|  | rs2737252 | 0.039848 | 0.002087 | 0.02499 | 364.5084 | 7.9E-89 |
|  | rs7017252 | 0.014111 | 0.001933 | 0.009556 | 53.2674 | 3.2E-13 |
|  | rs749368 | 0.031688 | 0.003935 | 0.010544 | 64.852 | 1.3E-17 |
|  | rs77599507 | -0.03377 | 0.003318 | -0.01332 | 103.5579 | 2.5E-23 |
|  | rs2128944 | -0.01175 | 0.002124 | -0.00724 | 30.60928 | 8.8E-09 |
|  | rs523374 | -0.01223 | 0.002054 | -0.0078 | 35.47674 | 7.8E-11 |
|  | rs1392788 | -0.0116 | 0.001889 | -0.00804 | 37.72524 | 2.8E-10 |
|  | rs7826493 | 0.023312 | 0.002327 | 0.013113 | 100.3216 | 2.4E-25 |
|  | rs7000279 | 0.013475 | 0.00213 | 0.008284 | 40.03114 | 3E-11 |
|  | rs2442599 | 0.014412 | 0.002107 | 0.008956 | 46.78891 | 6E-12 |
|  | rs6557839 | 0.015416 | 0.001961 | 0.010293 | 61.80243 | 1.3E-15 |
|  | rs4739697 | -0.0203 | 0.001991 | -0.01335 | 103.9935 | 3.3E-28 |
|  | rs36116061 | -0.01351 | 0.002382 | -0.00743 | 32.17768 | 2.3E-08 |
|  | rs1487241 | -0.02726 | 0.00201 | -0.01775 | 183.8429 | 4.1E-45 |
|  | rs680567 | 0.014297 | 0.002087 | 0.00897 | 46.9377 | 4E-12 |
|  | rs1078081 | -0.01627 | 0.002426 | -0.00878 | 44.96638 | 3.7E-11 |
|  | rs11142400 | 0.012952 | 0.001996 | 0.008495 | 42.10158 | 4.1E-10 |
|  | rs10760442 | 0.012389 | 0.001939 | 0.008367 | 40.83599 | 5.9E-11 |
|  | rs12340775 | -0.04125 | 0.004221 | -0.0128 | 95.51916 | 1.8E-22 |
|  | rs13293062 | -0.02283 | 0.003771 | -0.00793 | 36.67086 | 1E-09 |
|  | rs1022834 | -0.01486 | 0.001972 | -0.00987 | 56.77485 | 7.3E-14 |
|  | rs67624512 | 0.016199 | 0.002111 | 0.010049 | 58.91461 | 6.1E-14 |
|  | rs2900220 | 0.011278 | 0.001926 | 0.007668 | 34.30207 | 2.6E-09 |
|  | rs1934280 | -0.01356 | 0.002516 | -0.00706 | 29.06495 | 7.2E-09 |
|  | rs10124307 | -0.01263 | 0.001935 | -0.00854 | 42.56491 | 5.5E-11 |
|  | rs6559947 | 0.015255 | 0.002112 | 0.009457 | 52.17775 | 4.5E-14 |
|  | rs4743930 | 0.028842 | 0.002158 | 0.017493 | 178.5466 | 1.2E-42 |
|  | rs7027772 | -0.02542 | 0.002058 | -0.01617 | 152.5272 | 3.5E-35 |
|  | rs7024195 | 0.018134 | 0.002151 | 0.01104 | 71.09882 | 1.9E-18 |
|  | rs7871955 | 0.013172 | 0.002035 | 0.008475 | 41.89751 | 1.4E-11 |
|  | rs9657746 | -0.02651 | 0.001975 | -0.01757 | 180.1695 | 1.8E-45 |
|  | rs3829848 | 0.017454 | 0.001887 | 0.012112 | 85.58341 | 1E-19 |
|  | rs4363285 | -0.01472 | 0.002588 | -0.00745 | 32.36876 | 9.4E-10 |
|  | rs10739971 | 0.020557 | 0.002009 | 0.013395 | 104.6792 | 5.7E-29 |
|  | rs79158536 | 0.038341 | 0.003513 | 0.01429 | 119.1351 | 2.9E-30 |
|  | rs4961733 | 0.027413 | 0.002358 | 0.015217 | 135.104 | 1.9E-31 |
|  | rs7039458 | 0.011145 | 0.002167 | 0.006734 | 26.44878 | 2.5E-08 |
|  | rs28504650 | -0.02863 | 0.001963 | -0.01909 | 212.7602 | 1.3E-51 |
|  | rs2491105 | 0.022648 | 0.002235 | 0.013268 | 102.7102 | 7E-23 |
|  | rs630510 | -0.00978 | 0.001875 | -0.00683 | 27.20223 | 5.2E-09 |
|  | rs7906744 | -0.01735 | 0.002108 | -0.01078 | 67.76327 | 3.9E-17 |
|  | rs10795075 | -0.01739 | 0.00188 | -0.01211 | 85.54899 | 2.5E-21 |
|  | rs17688827 | -0.01986 | 0.001888 | -0.01377 | 110.6783 | 1.4E-28 |
|  | rs72809678 | -0.06137 | 0.008992 | -0.00894 | 46.58963 | 7.1E-13 |
|  | rs10159685 | 0.013084 | 0.002187 | 0.007834 | 35.79845 | 1.5E-09 |
|  | rs12242541 | -0.03631 | 0.00688 | -0.00691 | 27.86087 | 2.7E-08 |
|  | rs1649059 | -0.01733 | 0.001872 | -0.01212 | 85.71586 | 9.1E-21 |
|  | rs146232160 | -0.01533 | 0.002522 | -0.00796 | 36.95533 | 1.1E-09 |
|  | rs12262228 | 0.013829 | 0.001867 | 0.009696 | 54.84821 | 6.2E-13 |
|  | rs147819275 | 0.022173 | 0.003418 | 0.008495 | 42.09337 | 1.3E-11 |
|  | rs12358555 | 0.018829 | 0.002183 | 0.011291 | 74.37275 | 3.5E-18 |
|  | rs11196170 | -0.02878 | 0.002307 | -0.01633 | 155.6564 | 4.7E-37 |
|  | rs7080479 | 0.017041 | 0.001958 | 0.011392 | 75.71154 | 9.1E-19 |
|  | rs2015176 | -0.01181 | 0.002265 | -0.00683 | 27.17699 | 2.6E-08 |
|  | rs4881483 | 0.012161 | 0.001972 | 0.008075 | 38.04057 | 1.6E-10 |
|  | rs12359178 | -0.01438 | 0.002649 | -0.0071 | 29.4439 | 2.2E-08 |
|  | rs74119759 | -0.02797 | 0.00257 | -0.01425 | 118.4641 | 1.7E-30 |
|  | rs137979902 | 0.057432 | 0.006778 | 0.011093 | 71.79077 | 3.3E-19 |
|  | rs4917613 | -0.01321 | 0.001882 | -0.00919 | 49.23386 | 2E-12 |
|  | rs115223355 | 0.02493 | 0.004429 | 0.007369 | 31.67826 | 1.6E-08 |
|  | rs67771234 | 0.011911 | 0.002209 | 0.007059 | 29.06627 | 4.6E-08 |
|  | rs1148175 | 0.014023 | 0.001888 | 0.009724 | 55.15632 | 1.6E-15 |
|  | rs11238756 | 0.012447 | 0.001873 | 0.008703 | 44.1835 | 2.6E-13 |
|  | rs11002989 | -0.13349 | 0.003035 | -0.05749 | 1934.38 | 1E-200 |
|  | rs1912617 | 0.014569 | 0.002172 | 0.008784 | 45.0082 | 6.2E-12 |
|  | rs2579753 | -0.01861 | 0.002481 | -0.00982 | 56.25438 | 1.6E-14 |
|  | rs1463598 | 0.015867 | 0.001945 | 0.010682 | 66.56915 | 3.1E-16 |
|  | rs17173698 | 0.052186 | 0.005968 | 0.011448 | 76.45983 | 1.1E-17 |
|  | rs603424 | -0.02559 | 0.002476 | -0.01353 | 106.8274 | 1.2E-26 |
|  | rs947091 | 0.040502 | 0.001873 | 0.028298 | 467.4783 | 2.8E-109 |
|  | rs1159798 | -0.06723 | 0.002234 | -0.03937 | 905.3543 | 1E-200 |
|  | rs10740042 | 0.014599 | 0.002024 | 0.009446 | 52.04817 | 2.9E-12 |
|  | rs1272351 | 0.011157 | 0.001888 | 0.007739 | 34.93375 | 1.2E-09 |
|  | rs4979905 | -0.02883 | 0.002427 | -0.01555 | 141.0756 | 1.9E-34 |
|  | rs10885434 | -0.02159 | 0.002085 | -0.01356 | 107.2262 | 1.9E-26 |
|  | rs2672601 | 0.019586 | 0.002598 | 0.00987 | 56.8246 | 1.7E-14 |
|  | rs1133400 | 0.029824 | 0.00225 | 0.017355 | 175.7468 | 6.8E-43 |
|  | rs1484434 | -0.01067 | 0.002059 | -0.00679 | 26.87851 | 4.3E-08 |
|  | rs387619 | 0.028024 | 0.001871 | 0.019606 | 224.3135 | 1.8E-54 |
|  | rs11228240 | -0.04243 | 0.002083 | -0.02666 | 414.9985 | 2.8E-99 |
|  | rs608966 | -0.06911 | 0.002004 | -0.04512 | 1189.934 | 1E-200 |
|  | rs10765567 | -0.01645 | 0.001926 | -0.01118 | 72.92926 | 8.7E-20 |
|  | rs670401 | 0.012256 | 0.002065 | 0.007772 | 35.24009 | 7.7E-09 |
|  | rs10835060 | -0.01434 | 0.002358 | -0.00796 | 36.97096 | 3.2E-09 |
|  | rs2653559 | -0.02982 | 0.002505 | -0.01558 | 141.6644 | 5.3E-36 |
|  | rs111838776 | -0.0455 | 0.006326 | -0.00942 | 51.7422 | 4.4E-13 |
|  | rs1834554 | -0.02304 | 0.00246 | -0.01226 | 87.70577 | 4E-20 |
|  | rs7121746 | -0.05875 | 0.0019 | -0.04045 | 956.1058 | 1E-200 |
|  | rs2509353 | -0.02209 | 0.001874 | -0.01543 | 138.9734 | 5.1E-33 |
|  | rs611307 | 0.015524 | 0.001977 | 0.010282 | 61.67418 | 7.2E-16 |
|  | rs17507577 | 0.058327 | 0.003536 | 0.021592 | 272.0683 | 7.2E-67 |
|  | rs11023882 | -0.01371 | 0.00187 | -0.0096 | 53.77103 | 3.9E-13 |
|  | rs4595506 | -0.0212 | 0.001864 | -0.01489 | 129.4201 | 1.2E-30 |
|  | rs1519479 | -0.01299 | 0.001863 | -0.00913 | 48.63914 | 2.4E-12 |
|  | rs7118404 | -0.03402 | 0.00199 | -0.02239 | 292.4666 | 1.3E-73 |
|  | rs117111740 | -0.11325 | 0.00587 | -0.02525 | 372.2564 | 2.6E-86 |
|  | rs10896426 | 0.026775 | 0.001874 | 0.018707 | 204.1978 | 1.1E-48 |
|  | rs2894965 | -0.01865 | 0.003211 | -0.00761 | 33.7463 | 6E-10 |
|  | rs61918361 | -0.0104 | 0.00187 | -0.00728 | 30.92517 | 2.1E-08 |
|  | rs1261876 | -0.09389 | 0.007006 | -0.01754 | 179.5716 | 1.7E-43 |
|  | rs564614 | 0.011223 | 0.001964 | 0.00748 | 32.64076 | 1.6E-08 |
|  | rs7123749 | -0.04793 | 0.001939 | -0.03236 | 611.3703 | 3.4E-148 |
|  | rs11602333 | -0.02467 | 0.004029 | -0.00802 | 37.49788 | 9.1E-10 |
|  | rs183084030 | 0.034694 | 0.004948 | 0.00918 | 49.16424 | 3.5E-12 |
|  | rs1622638 | 0.014336 | 0.001904 | 0.009857 | 56.68491 | 2.1E-13 |
|  | rs35549817 | 0.01145 | 0.001929 | 0.00777 | 35.22131 | 2.2E-10 |
|  | rs1866818 | 0.027685 | 0.005098 | 0.007109 | 29.48449 | 4.4E-08 |
|  | rs174598 | 0.014068 | 0.001989 | 0.009261 | 50.03087 | 2.8E-13 |
|  | rs10750766 | 0.02375 | 0.002054 | 0.01514 | 133.7349 | 2.2E-32 |
|  | rs7125361 | 0.034895 | 0.001891 | 0.024154 | 340.5164 | 1.1E-80 |
|  | rs35223785 | 0.037783 | 0.00217 | 0.022794 | 303.2197 | 3.8E-69 |
|  | rs71457140 | 0.023219 | 0.002421 | 0.012554 | 91.94898 | 5.7E-23 |
|  | rs11175773 | 0.024288 | 0.003243 | 0.009806 | 56.09584 | 4.2E-13 |
|  | rs2887623 | -0.01743 | 0.001971 | -0.01158 | 78.19508 | 6.7E-20 |
|  | rs117481343 | 0.128746 | 0.005736 | 0.029378 | 503.8687 | 9.9E-120 |
|  | rs118115924 | -0.19925 | 0.008671 | -0.03007 | 527.9756 | 4.4E-121 |
|  | rs34833957 | -0.01269 | 0.002036 | -0.00816 | 38.84792 | 1.8E-10 |
|  | rs55849728 | 0.053815 | 0.001906 | 0.036949 | 797.4644 | 2.2E-186 |
|  | rs2374653 | -0.0118 | 0.001875 | -0.00824 | 39.64228 | 9.4E-11 |
|  | rs4759545 | -0.01444 | 0.001918 | -0.00986 | 56.67359 | 1.4E-15 |
|  | rs215224 | 0.025228 | 0.001911 | 0.017283 | 174.2865 | 1.1E-41 |
|  | rs111490848 | 0.013472 | 0.002055 | 0.008584 | 42.97997 | 3E-11 |
|  | rs12228756 | 0.0328 | 0.003788 | 0.011336 | 74.96687 | 2.9E-19 |
|  | rs1581630 | -0.02294 | 0.002224 | -0.0135 | 106.3572 | 7.2E-26 |
|  | rs79640667 | 0.031134 | 0.003685 | 0.011061 | 71.38069 | 8.6E-18 |
|  | rs7313563 | 0.01192 | 0.001968 | 0.007929 | 36.6715 | 2.1E-09 |
|  | rs2241820 | 0.016523 | 0.001903 | 0.011369 | 75.401 | 1.2E-17 |
|  | rs56043651 | 0.014407 | 0.002465 | 0.007652 | 34.15267 | 6.1E-10 |
|  | rs7135535 | -0.01501 | 0.00188 | -0.01045 | 63.73752 | 2E-17 |
|  | rs2583223 | 0.015017 | 0.001994 | 0.009859 | 56.70523 | 2.2E-15 |
|  | rs112073168 | -0.04149 | 0.005707 | -0.00952 | 52.85369 | 1.9E-14 |
|  | rs3963361 | -0.01132 | 0.001889 | -0.00784 | 35.87969 | 3E-09 |
|  | rs61921611 | 0.019608 | 0.002027 | 0.012665 | 93.58115 | 6.9E-23 |
|  | rs61754233 | -0.04568 | 0.006685 | -0.00895 | 46.69559 | 5E-12 |
|  | rs10777536 | 0.016027 | 0.001874 | 0.011195 | 73.11443 | 3.2E-17 |
|  | rs11107263 | 0.018626 | 0.002808 | 0.008685 | 44.00532 | 7.2E-12 |
|  | rs78985577 | 0.019001 | 0.002687 | 0.009258 | 50.00064 | 4.4E-14 |
|  | rs34373370 | -0.01377 | 0.002602 | -0.00693 | 27.98151 | 4.3E-08 |
|  | rs2887569 | -0.02092 | 0.001894 | -0.01446 | 122.0172 | 5E-30 |
|  | rs2430689 | -0.01498 | 0.00191 | -0.01027 | 61.56835 | 2.7E-16 |
|  | rs7969486 | 0.01253 | 0.001882 | 0.008718 | 44.33563 | 2.2E-11 |
|  | rs1872992 | 0.02866 | 0.002173 | 0.017266 | 173.9469 | 2.6E-40 |
|  | rs143348825 | -0.05174 | 0.007213 | -0.00939 | 51.453 | 1.7E-14 |
|  | rs11175835 | -0.02343 | 0.002066 | -0.01485 | 128.5833 | 5.4E-31 |
|  | rs11067228 | 0.015829 | 0.001875 | 0.011055 | 71.30336 | 2E-19 |
|  | rs12824957 | -0.02274 | 0.002334 | -0.01275 | 94.87728 | 3.8E-25 |
|  | rs8002850 | -0.02891 | 0.001989 | -0.01903 | 211.2749 | 2.8E-51 |
|  | rs76983463 | 0.03808 | 0.004723 | 0.010556 | 65.01103 | 1.4E-15 |
|  | rs34144741 | 0.023791 | 0.003618 | 0.008609 | 43.23095 | 9.8E-12 |
|  | rs56150718 | -0.01722 | 0.002985 | -0.00755 | 33.28981 | 7.7E-09 |
|  | rs912146 | 0.024926 | 0.002579 | 0.012652 | 93.39311 | 5E-24 |
|  | rs2812243 | 0.025799 | 0.003152 | 0.010716 | 66.98666 | 2.2E-16 |
|  | rs4307828 | -0.01088 | 0.001903 | -0.00748 | 32.64946 | 8.2E-10 |
|  | rs7329483 | -0.02412 | 0.002922 | -0.01081 | 68.14692 | 1E-17 |
|  | rs9594738 | -0.048 | 0.001877 | -0.03346 | 653.7185 | 9.4E-152 |
|  | rs3118906 | 0.028499 | 0.00209 | 0.017847 | 185.8591 | 4.2E-43 |
|  | rs3812849 | -0.01876 | 0.00213 | -0.01153 | 77.55716 | 6.4E-20 |
|  | rs112616813 | -0.06028 | 0.007065 | -0.01117 | 72.80283 | 3.3E-18 |
|  | rs7983462 | -0.03501 | 0.002043 | -0.02243 | 293.5531 | 5.4E-70 |
|  | rs9521510 | -0.02037 | 0.002013 | -0.01325 | 102.352 | 5.5E-27 |
|  | rs78667121 | 0.053781 | 0.005438 | 0.012948 | 97.80516 | 9.1E-25 |
|  | rs9545559 | 0.00968 | 0.001876 | 0.006755 | 26.62001 | 4.3E-08 |
|  | rs9557349 | -0.02219 | 0.002346 | -0.01238 | 89.44352 | 6.6E-23 |
|  | rs10145299 | 0.022277 | 0.001892 | 0.015415 | 138.6321 | 2.1E-30 |
|  | rs56744189 | -0.01762 | 0.002107 | -0.01095 | 69.92334 | 5.6E-19 |
|  | rs1286075 | 0.030758 | 0.002485 | 0.016203 | 153.1726 | 1.2E-38 |
|  | rs1042704 | -0.02804 | 0.00229 | -0.01603 | 149.9346 | 3.4E-34 |
|  | rs75663481 | 0.020458 | 0.002137 | 0.012533 | 91.64493 | 1.2E-22 |
|  | rs72707344 | 0.015207 | 0.002655 | 0.0075 | 32.81403 | 4.8E-09 |
|  | rs1209075 | -0.02556 | 0.003884 | -0.00862 | 43.32499 | 1.1E-11 |
|  | rs210374 | 0.013295 | 0.002127 | 0.008184 | 39.07536 | 4.7E-11 |
|  | rs12590407 | 0.017222 | 0.002063 | 0.010928 | 69.66973 | 4.5E-17 |
|  | rs71422098 | -0.01942 | 0.003052 | -0.00833 | 40.5003 | 4E-11 |
|  | rs35107139 | 0.059208 | 0.001979 | 0.039152 | 895.5174 | 1E-200 |
|  | rs76172201 | 0.04138 | 0.007094 | 0.007638 | 34.02907 | 5.6E-11 |
|  | rs13379119 | 0.01685 | 0.001883 | 0.011717 | 80.09007 | 4.1E-20 |
|  | rs11627442 | -0.01694 | 0.001893 | -0.01172 | 80.10462 | 9.7E-19 |
|  | rs12323717 | 0.014921 | 0.001969 | 0.00992 | 57.40252 | 1.1E-14 |
|  | rs3783727 | 0.014347 | 0.001986 | 0.009457 | 52.17532 | 2.9E-14 |
|  | rs78932264 | 0.02319 | 0.003983 | 0.007622 | 33.89151 | 1.1E-09 |
|  | rs62007686 | -0.04332 | 0.001976 | -0.0287 | 480.8609 | 1.2E-111 |
|  | rs112766772 | 0.015841 | 0.002262 | 0.009169 | 49.03937 | 1.5E-13 |
|  | rs74710884 | -0.02349 | 0.003849 | -0.00799 | 37.26002 | 2.3E-10 |
|  | rs34317102 | -0.01231 | 0.002268 | -0.00711 | 29.48122 | 1.3E-08 |
|  | rs868127 | -0.0179 | 0.002106 | -0.01113 | 72.25057 | 4E-18 |
|  | rs2310792 | 0.018478 | 0.003072 | 0.007876 | 36.1873 | 4.8E-08 |
|  | rs17601876 | 0.029769 | 0.001883 | 0.020697 | 249.9845 | 3.5E-60 |
|  | rs12903143 | 0.031467 | 0.00575 | 0.007165 | 29.94407 | 2.3E-08 |
|  | rs12443188 | 0.02335 | 0.002235 | 0.013678 | 109.1466 | 9.3E-27 |
|  | rs2456049 | 0.010458 | 0.001958 | 0.006992 | 28.51602 | 3.9E-08 |
|  | rs7167692 | -0.05019 | 0.004215 | -0.01559 | 141.8349 | 1.3E-35 |
|  | rs28840973 | 0.023045 | 0.004117 | 0.007329 | 31.33344 | 2.7E-08 |
|  | rs61998565 | -0.01776 | 0.002671 | -0.0087 | 44.18825 | 2.1E-11 |
|  | rs59429575 | 0.021362 | 0.002161 | 0.01294 | 97.69169 | 1.4E-23 |
|  | rs4299103 | -0.01565 | 0.002008 | -0.0102 | 60.73118 | 2E-14 |
|  | rs35713954 | -0.01066 | 0.001903 | -0.00734 | 31.38627 | 1.2E-08 |
|  | rs1706708 | -0.02774 | 0.002007 | -0.0181 | 191.1335 | 5.3E-50 |
|  | rs11632673 | 0.02436 | 0.001891 | 0.016863 | 165.9246 | 4.8E-43 |
|  | rs6600235 | -0.01172 | 0.002222 | -0.00691 | 27.81697 | 1.2E-08 |
|  | rs2052354 | 0.02049 | 0.00214 | 0.012538 | 91.71308 | 1.3E-22 |
|  | rs72805220 | -0.06013 | 0.003782 | -0.02081 | 252.8025 | 2.9E-59 |
|  | rs17680862 | -0.07717 | 0.00587 | -0.01721 | 172.849 | 5.2E-43 |
|  | rs7198843 | 0.013745 | 0.001899 | 0.009474 | 52.36564 | 5.8E-14 |
|  | rs12918989 | 0.010733 | 0.001932 | 0.007272 | 30.84503 | 4.4E-08 |
|  | rs71378512 | -0.09572 | 0.004888 | -0.02563 | 383.4374 | 2E-88 |
|  | rs180831566 | 0.021817 | 0.003179 | 0.008986 | 47.1034 | 1.1E-11 |
|  | rs2531992 | 0.023693 | 0.002596 | 0.011949 | 83.30264 | 9.2E-22 |
|  | rs78124868 | 0.023871 | 0.003419 | 0.009141 | 48.74568 | 4.7E-13 |
|  | rs1585528 | 0.031802 | 0.002238 | 0.018601 | 201.8952 | 3.8E-51 |
|  | rs8056705 | 0.022986 | 0.004001 | 0.007522 | 33.00922 | 2.9E-09 |
|  | rs4081747 | -0.01323 | 0.001948 | -0.00889 | 46.13605 | 7.8E-11 |
|  | rs17232812 | -0.01365 | 0.002558 | -0.00699 | 28.49006 | 4.8E-08 |
|  | rs2386890 | -0.01283 | 0.002008 | -0.00836 | 40.81297 | 3.6E-12 |
|  | rs62038775 | 0.018751 | 0.002223 | 0.011045 | 71.16583 | 7E-19 |
|  | rs4967985 | -0.01895 | 0.002202 | -0.01127 | 74.05136 | 9.3E-19 |
|  | rs12917722 | 0.012947 | 0.001908 | 0.008885 | 46.05428 | 4.8E-13 |
|  | rs28571100 | 0.020177 | 0.003272 | 0.008073 | 38.01961 | 1.4E-10 |
|  | rs7102 | -0.02022 | 0.001951 | -0.01357 | 107.4736 | 1.3E-26 |
|  | rs62028332 | 0.046173 | 0.00279 | 0.021662 | 273.8411 | 4.2E-68 |
|  | rs7501078 | -0.02233 | 0.001921 | -0.01522 | 135.1342 | 2.3E-33 |
|  | rs1000454 | 0.023825 | 0.001884 | 0.016558 | 159.9676 | 5.3E-38 |
|  | rs71390846 | -0.04272 | 0.002408 | -0.02323 | 314.8326 | 6.1E-78 |
|  | rs4782351 | -0.01529 | 0.001924 | -0.01041 | 63.16688 | 7.2E-16 |
|  | rs2272443 | -0.02059 | 0.002248 | -0.01199 | 83.89349 | 1.3E-20 |
|  | rs11657636 | 0.051373 | 0.002035 | 0.033036 | 637.2959 | 1.8E-149 |
|  | rs11653826 | -0.0207 | 0.003187 | -0.00851 | 42.21129 | 4.8E-10 |
|  | rs9896306 | -0.02621 | 0.002103 | -0.01632 | 155.3193 | 7.4E-36 |
|  | rs72856728 | -0.02885 | 0.004532 | -0.00834 | 40.52842 | 1.6E-10 |
|  | rs62063906 | 0.020484 | 0.003568 | 0.007518 | 32.9672 | 3.1E-09 |
|  | rs55888531 | -0.02193 | 0.001981 | -0.01449 | 122.5527 | 3.3E-30 |
|  | rs76833657 | -0.04408 | 0.003549 | -0.01626 | 154.2875 | 3.4E-37 |
|  | rs3760456 | -0.02405 | 0.001885 | -0.0167 | 162.7835 | 1.2E-38 |
|  | rs315464 | 0.011103 | 0.001903 | 0.007639 | 34.04076 | 8.8E-09 |
|  | rs9898613 | 0.033436 | 0.002137 | 0.02048 | 244.765 | 2.3E-57 |
|  | rs57043009 | 0.054453 | 0.002638 | 0.027015 | 426.0217 | 3.3E-98 |
|  | rs12945403 | -0.01658 | 0.001983 | -0.01094 | 69.88132 | 6.2E-18 |
|  | rs2663351 | -0.02431 | 0.001982 | -0.01606 | 150.4192 | 2E-37 |
|  | rs241770 | 0.012734 | 0.002033 | 0.0082 | 39.22651 | 5.2E-11 |
|  | rs7212160 | -0.01658 | 0.002163 | -0.01004 | 58.80085 | 2E-15 |
|  | rs56107545 | 0.011246 | 0.001921 | 0.007664 | 34.26777 | 4.8E-09 |
|  | rs60891864 | 0.029303 | 0.001907 | 0.020118 | 236.189 | 1.7E-52 |
|  | rs1029830 | -0.01435 | 0.001871 | -0.01004 | 58.79081 | 7.9E-15 |
|  | rs2075060 | -0.01459 | 0.001887 | -0.01012 | 59.77716 | 1E-15 |
|  | rs1724409 | 0.022355 | 0.002143 | 0.013656 | 108.8008 | 3.3E-27 |
|  | rs61749930 | -0.03512 | 0.005299 | -0.00868 | 43.93578 | 1.2E-10 |
|  | rs4969187 | -0.01591 | 0.00195 | -0.01068 | 66.5671 | 1.7E-16 |
|  | rs8080283 | -0.01309 | 0.001883 | -0.0091 | 48.31788 | 2.7E-13 |
|  | rs12942138 | -0.01891 | 0.002354 | -0.01052 | 64.55384 | 1.9E-16 |
|  | rs9896203 | -0.01455 | 0.002034 | -0.00936 | 51.13684 | 4.3E-12 |
|  | rs12449814 | 0.034179 | 0.005074 | 0.00882 | 45.37647 | 2.2E-10 |
|  | rs2741856 | 0.074507 | 0.003416 | 0.028549 | 475.8201 | 7.2E-111 |
|  | rs11652501 | 0.019604 | 0.001945 | 0.013196 | 101.5961 | 1.4E-23 |
|  | rs4798783 | 0.012503 | 0.002061 | 0.007943 | 36.804 | 1.9E-10 |
|  | rs78015143 | 0.02779 | 0.003158 | 0.011521 | 77.43454 | 8E-21 |
|  | rs1941135 | -0.01704 | 0.003207 | -0.00695 | 28.21231 | 1.6E-08 |
|  | rs4635400 | -0.05116 | 0.001957 | -0.03421 | 683.4062 | 3.3E-162 |
|  | rs884205 | 0.023543 | 0.002179 | 0.014148 | 116.7797 | 4.2E-29 |
|  | rs4940236 | 0.016889 | 0.001962 | 0.011272 | 74.12439 | 8.3E-19 |
|  | rs7236090 | 0.012984 | 0.001891 | 0.008991 | 47.15398 | 1.9E-13 |
|  | rs11660070 | -0.01313 | 0.002225 | -0.00773 | 34.82216 | 5.5E-10 |
|  | rs206432 | 0.015741 | 0.001883 | 0.010944 | 69.86665 | 2.4E-16 |
|  | rs957772 | -0.01389 | 0.001895 | -0.0096 | 53.76591 | 6.7E-13 |
|  | rs4369779 | -0.01933 | 0.0023 | -0.011 | 70.59577 | 6.5E-18 |
|  | rs8095921 | 0.015645 | 0.002263 | 0.009052 | 47.80091 | 3.7E-13 |
|  | rs35518690 | 0.01278 | 0.002059 | 0.008127 | 38.52488 | 1E-09 |
|  | rs2337106 | 0.018419 | 0.001891 | 0.01275 | 94.84634 | 3.4E-24 |
|  | rs72966926 | 0.014661 | 0.002797 | 0.006863 | 27.47388 | 2.4E-08 |
|  | rs3848474 | 0.014528 | 0.001897 | 0.010025 | 58.62953 | 1.3E-15 |
|  | rs8104911 | 0.103915 | 0.003233 | 0.042054 | 1033.42 | 1E-200 |
|  | rs10407062 | -0.01311 | 0.001889 | -0.00909 | 48.1959 | 8.1E-13 |
|  | rs1122233 | -0.0271 | 0.004162 | -0.00852 | 42.39205 | 2.7E-10 |
|  | rs147496952 | -0.03146 | 0.00502 | -0.0082 | 39.26223 | 2.5E-11 |
|  | rs12984621 | 0.016717 | 0.002057 | 0.010642 | 66.07386 | 4.2E-16 |
|  | rs11880992 | -0.01958 | 0.001909 | -0.01343 | 105.2577 | 3.4E-26 |
|  | rs59813731 | 0.023615 | 0.002375 | 0.013019 | 98.88638 | 5.1E-24 |
|  | rs12459676 | 0.014259 | 0.002476 | 0.00754 | 33.1651 | 1.1E-09 |
|  | rs28364580 | -0.02161 | 0.002195 | -0.01289 | 96.91755 | 2.7E-24 |
|  | rs57813069 | -0.0348 | 0.002027 | -0.02248 | 294.888 | 1.3E-67 |
|  | rs4806862 | 0.021241 | 0.002013 | 0.013816 | 111.3636 | 5.9E-27 |
|  | rs60018147 | -0.03269 | 0.003029 | -0.01413 | 116.4347 | 7.5E-28 |
|  | rs11670056 | -0.03039 | 0.003548 | -0.01122 | 73.37968 | 1.9E-18 |
|  | rs7251807 | -0.02404 | 0.002917 | -0.01079 | 67.92952 | 1.8E-17 |
|  | rs425105 | -0.01882 | 0.002537 | -0.00971 | 55.02915 | 7.3E-15 |
|  | rs1004246 | 0.01176 | 0.00212 | 0.007262 | 30.75954 | 3.9E-08 |
|  | rs56242234 | -0.0113 | 0.001919 | -0.00771 | 34.64897 | 2.2E-10 |
|  | rs4455225 | -0.01182 | 0.001906 | -0.00812 | 38.47913 | 6.6E-10 |
|  | rs1206760 | 0.020024 | 0.001899 | 0.013805 | 111.1854 | 2.1E-26 |
|  | rs35308216 | -0.06127 | 0.003418 | -0.02346 | 321.2645 | 4.4E-77 |
|  | rs293736 | -0.0129 | 0.002098 | -0.00805 | 37.77347 | 1.1E-10 |
|  | rs7264777 | 0.020951 | 0.001953 | 0.014047 | 115.1251 | 3.3E-29 |
|  | rs6085968 | 0.019222 | 0.003213 | 0.007833 | 35.78997 | 7.5E-10 |
|  | rs6117854 | -0.03805 | 0.002017 | -0.02469 | 355.7351 | 2.6E-87 |
|  | rs6120804 | 0.024894 | 0.002445 | 0.013332 | 103.703 | 1.6E-26 |
|  | rs928486 | 0.016715 | 0.002274 | 0.009626 | 54.05454 | 5.5E-13 |
|  | rs1973924 | 0.031019 | 0.003296 | 0.012323 | 88.58659 | 2.9E-21 |
|  | rs6040286 | 0.035978 | 0.001892 | 0.024891 | 361.6124 | 1.7E-83 |
|  | rs13042961 | -0.04888 | 0.0047 | -0.01362 | 108.1487 | 9.4E-27 |
|  | rs6129493 | -0.01435 | 0.002101 | -0.00894 | 46.65934 | 2.5E-11 |
|  | rs17265513 | -0.03479 | 0.002351 | -0.01937 | 219.0362 | 2.6E-55 |
|  | rs12482821 | 0.064928 | 0.006438 | 0.013204 | 101.7144 | 9.4E-27 |
|  | rs9974172 | 0.017321 | 0.002681 | 0.008458 | 41.7277 | 1.8E-11 |
|  | rs11088458 | -0.04752 | 0.002097 | -0.02965 | 513.2926 | 1.3E-120 |
|  | rs56178309 | -0.01267 | 0.002315 | -0.00717 | 29.94795 | 2.7E-08 |
|  | rs9983027 | 0.013424 | 0.002278 | 0.007715 | 34.71833 | 3.9E-10 |
|  | rs2836620 | 0.028245 | 0.002194 | 0.016856 | 165.7733 | 8.8E-41 |
|  | rs139791219 | -0.02824 | 0.004788 | -0.00772 | 34.78153 | 2.4E-10 |
|  | rs2830913 | 0.02373 | 0.001904 | 0.016313 | 155.268 | 1.7E-35 |
|  | rs2242761 | -0.01736 | 0.001978 | -0.01149 | 77.01584 | 6.9E-20 |
|  | rs9606139 | -0.11836 | 0.003088 | -0.05012 | 1468.739 | 1E-200 |
|  | rs73156468 | -0.01819 | 0.002697 | -0.00883 | 45.4872 | 1.1E-11 |
|  | rs55709850 | -0.01682 | 0.002082 | -0.01058 | 65.26097 | 1.5E-16 |
|  | rs134613 | -0.04405 | 0.001975 | -0.02919 | 497.5879 | 6.3E-118 |
|  | rs1056322 | -0.01249 | 0.002015 | -0.00811 | 38.39105 | 1.4E-10 |
|  | rs62228067 | 0.021499 | 0.002224 | 0.012654 | 93.41698 | 9.4E-25 |
|  | rs28707249 | -0.0238 | 0.003017 | -0.01033 | 62.22084 | 7.1E-16 |
|  | rs13057405 | 0.013784 | 0.002349 | 0.007683 | 34.43526 | 4.2E-10 |
|  | rs132530 | 0.056913 | 0.005307 | 0.014039 | 114.9964 | 1.2E-28 |
|  | rs7290979 | -0.02544 | 0.002082 | -0.016 | 149.3795 | 2.4E-39 |
|  | rs5750263 | 0.014969 | 0.002568 | 0.007633 | 33.98629 | 7E-10 |
| Hand grip strength (left) |  |  |  |  |  |  |
|  | rs6680160 | 0.010069 | 0.001539 | 0.009636 | 42.81393 | 6E-11 |
|  | rs7516571 | 0.009378 | 0.001694 | 0.008155 | 30.66139 | 3.1E-08 |
|  | rs150330307 | -0.03078 | 0.004217 | -0.01075 | 53.26712 | 2.9E-13 |
|  | rs2800789 | 0.00826 | 0.001493 | 0.00815 | 30.62624 | 3.1E-08 |
|  | rs1044299 | 0.01401 | 0.001494 | 0.013813 | 87.98245 | 6.6E-21 |
|  | rs11121542 | -0.01576 | 0.002259 | -0.01027 | 48.66581 | 3E-12 |
|  | rs4121165 | -0.01142 | 0.001819 | -0.00925 | 39.40795 | 3.4E-10 |
|  | rs58670122 | -0.01181 | 0.002145 | -0.00811 | 30.34954 | 3.6E-08 |
|  | rs10788958 | 0.014198 | 0.001563 | 0.013379 | 82.53636 | 1E-19 |
|  | rs4335354 | -0.00939 | 0.001603 | -0.00863 | 34.35962 | 4.6E-09 |
|  | rs1884447 | 0.008461 | 0.001514 | 0.008231 | 31.23284 | 2.3E-08 |
|  | rs61818100 | 0.013442 | 0.002313 | 0.008558 | 33.76432 | 6.2E-09 |
|  | rs823130 | -0.01133 | 0.001501 | -0.01112 | 56.99547 | 4.4E-14 |
|  | rs11204664 | -0.00865 | 0.0015 | -0.00849 | 33.2544 | 8.1E-09 |
|  | rs6689375 | -0.01602 | 0.001912 | -0.01234 | 70.20564 | 5.3E-17 |
|  | rs6433478 | 0.009057 | 0.0015 | 0.008895 | 36.47918 | 1.5E-09 |
|  | rs12473732 | 0.010987 | 0.001486 | 0.010888 | 54.66469 | 1.4E-13 |
|  | rs7571789 | 0.012954 | 0.001487 | 0.012829 | 75.88905 | 3E-18 |
|  | rs7575451 | -0.00973 | 0.001553 | -0.00923 | 39.2368 | 3.8E-10 |
|  | rs1434095 | 0.01403 | 0.002259 | 0.009146 | 38.57145 | 5.3E-10 |
|  | rs17630248 | -0.00918 | 0.001562 | -0.00866 | 34.58701 | 4.1E-09 |
|  | rs1981612 | 0.009228 | 0.001511 | 0.008997 | 37.31763 | 1E-09 |
|  | rs11125803 | 0.014285 | 0.001697 | 0.012399 | 70.88284 | 3.8E-17 |
|  | rs1641457 | 0.012046 | 0.001783 | 0.00995 | 45.64602 | 1.4E-11 |
|  | rs3819121 | 0.014096 | 0.001529 | 0.013579 | 85.02651 | 2.9E-20 |
|  | rs10176878 | -0.01295 | 0.001896 | -0.01006 | 46.62552 | 8.6E-12 |
|  | rs61286123 | -0.0101 | 0.001771 | -0.0084 | 32.54649 | 1.2E-08 |
|  | rs34030812 | -0.01017 | 0.00154 | -0.00972 | 43.56967 | 4.1E-11 |
|  | rs10205394 | -0.01132 | 0.001857 | -0.00898 | 37.18381 | 1.1E-09 |
|  | rs1551042 | -0.01113 | 0.001552 | -0.01056 | 51.43132 | 7.4E-13 |
|  | rs9866627 | -0.0156 | 0.002678 | -0.00858 | 33.93855 | 5.7E-09 |
|  | rs112485536 | 0.016178 | 0.002818 | 0.008455 | 32.95741 | 9.4E-09 |
|  | rs62253653 | 0.010699 | 0.001631 | 0.009657 | 43.00242 | 5.5E-11 |
|  | rs6802071 | -0.0094 | 0.001502 | -0.00922 | 39.20817 | 3.8E-10 |
|  | rs71298370 | 0.014876 | 0.002704 | 0.008102 | 30.26477 | 3.8E-08 |
|  | rs13091492 | -0.00848 | 0.001534 | -0.00814 | 30.54935 | 3.3E-08 |
|  | rs10934857 | 0.009287 | 0.001702 | 0.008037 | 29.77832 | 4.8E-08 |
|  | rs4498020 | -0.01042 | 0.00167 | -0.00919 | 38.96236 | 4.3E-10 |
|  | rs4677601 | 0.009059 | 0.001485 | 0.008984 | 37.21279 | 1.1E-09 |
|  | rs2871960 | 0.012088 | 0.001492 | 0.01193 | 65.61997 | 5.5E-16 |
|  | rs35609019 | 0.009477 | 0.001541 | 0.009059 | 37.83958 | 7.7E-10 |
|  | rs13107325 | -0.02616 | 0.002821 | -0.01365 | 85.97552 | 1.8E-20 |
|  | rs56338231 | -0.01085 | 0.001697 | -0.00941 | 40.8372 | 1.7E-10 |
|  | rs13146142 | -0.0202 | 0.002029 | -0.01466 | 99.15698 | 2.3E-23 |
|  | rs34587452 | -0.01136 | 0.001807 | -0.00926 | 39.53342 | 3.2E-10 |
|  | rs13106087 | 0.01162 | 0.001974 | 0.008669 | 34.65272 | 3.9E-09 |
|  | rs997850 | -0.00887 | 0.001528 | -0.00855 | 33.72691 | 6.3E-09 |
|  | rs34722008 | 0.008586 | 0.001555 | 0.008134 | 30.50407 | 3.3E-08 |
|  | rs2850379 | -0.00829 | 0.0015 | -0.00814 | 30.54083 | 3.3E-08 |
|  | rs75497896 | -0.02067 | 0.003375 | -0.00902 | 37.51328 | 9.1E-10 |
|  | rs116409670 | -0.01524 | 0.002739 | -0.0082 | 30.9737 | 2.6E-08 |
|  | rs55681913 | 0.013774 | 0.002437 | 0.008325 | 31.95554 | 1.6E-08 |
|  | rs13356200 | -0.00877 | 0.001529 | -0.00845 | 32.8987 | 9.7E-09 |
|  | rs2431112 | -0.00958 | 0.001495 | -0.00944 | 41.09764 | 1.4E-10 |
|  | rs2631360 | -0.01092 | 0.001484 | -0.01083 | 54.09978 | 1.9E-13 |
|  | rs6882168 | -0.00935 | 0.001574 | -0.00875 | 35.3323 | 2.8E-09 |
|  | rs113918482 | -0.0101 | 0.001785 | -0.00833 | 32.01754 | 1.5E-08 |
|  | rs2974438 | -0.01007 | 0.001824 | -0.00813 | 30.50514 | 3.3E-08 |
|  | rs185320691 | -0.01669 | 0.002689 | -0.00914 | 38.50398 | 5.5E-10 |
|  | rs12528131 | -0.00883 | 0.001486 | -0.00875 | 35.30855 | 2.8E-09 |
|  | rs9371201 | -0.00934 | 0.001574 | -0.00874 | 35.20889 | 3E-09 |
|  | rs77485342 | 0.032968 | 0.005582 | 0.008698 | 34.8829 | 3.5E-09 |
|  | rs9388769 | -0.01408 | 0.001581 | -0.01312 | 79.37129 | 5.1E-19 |
|  | rs35175534 | -0.01637 | 0.002355 | -0.01024 | 48.34766 | 3.6E-12 |
|  | rs113315602 | -0.01819 | 0.002663 | -0.01006 | 46.63473 | 8.6E-12 |
|  | rs723588 | 0.01279 | 0.002119 | 0.008887 | 36.41746 | 1.6E-09 |
|  | rs11243202 | 0.009722 | 0.001491 | 0.009604 | 42.52529 | 7E-11 |
|  | rs4713506 | -0.01573 | 0.001698 | -0.01364 | 85.7727 | 2E-20 |
|  | rs2038760 | -0.01153 | 0.001987 | -0.00854 | 33.66222 | 6.6E-09 |
|  | rs41271299 | 0.021189 | 0.003364 | 0.009276 | 39.67417 | 3E-10 |
|  | rs9371881 | 0.009479 | 0.00155 | 0.009007 | 37.4047 | 9.6E-10 |
|  | rs4621706 | -0.01171 | 0.0015 | -0.01149 | 60.9083 | 6E-15 |
|  | rs11769549 | 0.020496 | 0.003112 | 0.009698 | 43.36746 | 4.5E-11 |
|  | rs12533765 | -0.0092 | 0.001652 | -0.0082 | 31.01422 | 2.6E-08 |
|  | rs16870531 | 0.011198 | 0.001742 | 0.009465 | 41.30287 | 1.3E-10 |
|  | rs13227429 | -0.00859 | 0.001498 | -0.00844 | 32.87767 | 9.8E-09 |
|  | rs6977081 | 0.014753 | 0.001586 | 0.013695 | 86.47695 | 1.4E-20 |
|  | rs12673062 | -0.01077 | 0.001808 | -0.00877 | 35.47509 | 2.6E-09 |
|  | rs73307079 | 0.011103 | 0.001834 | 0.008915 | 36.64458 | 1.4E-09 |
|  | rs6962338 | -0.02141 | 0.003617 | -0.00872 | 35.03418 | 3.2E-09 |
|  | rs17282763 | 0.00894 | 0.001633 | 0.008062 | 29.96888 | 4.4E-08 |
|  | rs821100 | -0.0102 | 0.001686 | -0.00891 | 36.57733 | 1.5E-09 |
|  | rs59116179 | 0.008574 | 0.001536 | 0.008219 | 31.14379 | 2.4E-08 |
|  | rs4737446 | 0.010418 | 0.001619 | 0.009478 | 41.41474 | 1.2E-10 |
|  | rs1486925 | -0.01047 | 0.001602 | -0.00963 | 42.73569 | 6.3E-11 |
|  | rs4398863 | -0.00945 | 0.001685 | -0.00826 | 31.4713 | 2E-08 |
|  | rs4739739 | -0.00853 | 0.001505 | -0.00835 | 32.16735 | 1.4E-08 |
|  | rs10097417 | -0.01323 | 0.001971 | -0.00989 | 45.0773 | 1.9E-11 |
|  | rs7026798 | 0.008236 | 0.001506 | 0.008052 | 29.89126 | 4.6E-08 |
|  | rs7856625 | -0.01113 | 0.001521 | -0.01078 | 53.55075 | 2.5E-13 |
|  | rs16910750 | 0.01125 | 0.002041 | 0.008116 | 30.3695 | 3.6E-08 |
|  | rs116922558 | -0.02158 | 0.003854 | -0.00825 | 31.36116 | 2.1E-08 |
|  | rs2789514 | 0.012055 | 0.002209 | 0.008036 | 29.77065 | 4.9E-08 |
|  | rs10988217 | -0.00921 | 0.001529 | -0.00887 | 36.25086 | 1.7E-09 |
|  | rs11002322 | -0.00999 | 0.001573 | -0.00936 | 40.35115 | 2.1E-10 |
|  | rs10786706 | 0.010007 | 0.001487 | 0.009908 | 45.26513 | 1.7E-11 |
|  | rs35236379 | 0.012357 | 0.002122 | 0.008576 | 33.90657 | 5.8E-09 |
|  | rs11003014 | 0.011433 | 0.002021 | 0.00833 | 31.98917 | 1.6E-08 |
|  | rs4962700 | 0.009043 | 0.001635 | 0.008146 | 30.59567 | 3.2E-08 |
|  | rs772014 | -0.01062 | 0.001519 | -0.01029 | 48.8658 | 2.7E-12 |
|  | rs10821939 | -0.00935 | 0.001503 | -0.00916 | 38.71434 | 4.9E-10 |
|  | rs1556659 | 0.01629 | 0.001535 | 0.015632 | 112.6875 | 2.5E-26 |
|  | rs72977282 | -0.01556 | 0.001512 | -0.01515 | 105.9004 | 7.8E-25 |
|  | rs4930236 | 0.011903 | 0.002021 | 0.008675 | 34.69989 | 3.8E-09 |
|  | rs10831903 | 0.009287 | 0.001512 | 0.009048 | 37.74645 | 8.1E-10 |
|  | rs12790261 | -0.02519 | 0.002703 | -0.01372 | 86.83326 | 1.2E-20 |
|  | rs7124681 | -0.01166 | 0.001506 | -0.0114 | 59.88806 | 1E-14 |
|  | rs61389091 | 0.026163 | 0.003733 | 0.010322 | 49.12027 | 2.4E-12 |
|  | rs34845616 | 0.01084 | 0.001734 | 0.009208 | 39.08909 | 4E-10 |
|  | rs76895963 | 0.035963 | 0.005749 | 0.009213 | 39.13761 | 3.9E-10 |
|  | rs10846071 | -0.01664 | 0.001517 | -0.01616 | 120.3576 | 5.3E-28 |
|  | rs11168357 | -0.00963 | 0.001727 | -0.00821 | 31.09864 | 2.5E-08 |
|  | rs4575361 | -0.0108 | 0.001602 | -0.00993 | 45.45932 | 1.6E-11 |
|  | rs12316046 | -0.01742 | 0.00153 | -0.01676 | 129.5955 | 5E-30 |
|  | rs7970350 | -0.01013 | 0.001484 | -0.01006 | 46.62636 | 8.6E-12 |
|  | rs7963801 | -0.01043 | 0.001508 | -0.01019 | 47.86602 | 4.6E-12 |
|  | rs11111267 | 0.010836 | 0.001929 | 0.008273 | 31.55939 | 1.9E-08 |
|  | rs3118903 | -0.01745 | 0.001797 | -0.0143 | 94.27985 | 2.7E-22 |
|  | rs56060323 | 0.009069 | 0.001602 | 0.008335 | 32.03263 | 1.5E-08 |
|  | rs12889267 | -0.01374 | 0.001987 | -0.01018 | 47.79981 | 4.7E-12 |
|  | rs7148603 | 0.009572 | 0.001586 | 0.008888 | 36.4199 | 1.6E-09 |
|  | rs2359239 | -0.00881 | 0.001522 | -0.00853 | 33.56179 | 6.9E-09 |
|  | rs10144445 | -0.00937 | 0.001559 | -0.00885 | 36.10536 | 1.9E-09 |
|  | rs28542042 | 0.01101 | 0.001623 | 0.009992 | 46.03293 | 1.2E-11 |
|  | rs12906830 | 0.01084 | 0.001518 | 0.010517 | 50.99648 | 9.3E-13 |
|  | rs3959716 | -0.00833 | 0.001504 | -0.00815 | 30.62835 | 3.1E-08 |
|  | rs17466480 | -0.01182 | 0.001527 | -0.0114 | 59.92831 | 9.8E-15 |
|  | rs12914702 | 0.010951 | 0.001691 | 0.009539 | 41.95667 | 9.3E-11 |
|  | rs7176095 | -0.01338 | 0.00222 | -0.00887 | 36.28974 | 1.7E-09 |
|  | rs2871865 | -0.0218 | 0.002317 | -0.01386 | 88.53825 | 5E-21 |
|  | rs13337177 | -0.01428 | 0.001936 | -0.01086 | 54.3933 | 1.6E-13 |
|  | rs11642954 | -0.0117 | 0.001877 | -0.00918 | 38.87831 | 4.5E-10 |
|  | rs217181 | 0.011955 | 0.001881 | 0.009358 | 40.37782 | 2.1E-10 |
|  | rs9944324 | -0.00855 | 0.001502 | -0.00839 | 32.43754 | 1.2E-08 |
|  | rs7197751 | -0.00946 | 0.001563 | -0.00892 | 36.67219 | 1.4E-09 |
|  | rs7196917 | -0.01173 | 0.001501 | -0.01151 | 61.09989 | 5.4E-15 |
|  | rs181766 | 0.009632 | 0.001604 | 0.008846 | 36.07599 | 1.9E-09 |
|  | rs3814877 | 0.010539 | 0.001514 | 0.010252 | 48.46095 | 3.4E-12 |
|  | rs11076004 | -0.01154 | 0.001509 | -0.01126 | 58.43191 | 2.1E-14 |
|  | rs113434679 | -0.01492 | 0.001876 | -0.01171 | 63.20224 | 1.9E-15 |
|  | rs755547 | 0.016516 | 0.001898 | 0.012817 | 75.74464 | 3.2E-18 |
|  | rs2532111 | 0.010285 | 0.001558 | 0.009723 | 43.58369 | 4.1E-11 |
|  | rs999493 | 0.012871 | 0.001539 | 0.012314 | 69.91405 | 6.2E-17 |
|  | rs2587505 | -0.009 | 0.001507 | -0.0088 | 35.70279 | 2.3E-09 |
|  | rs635538 | -0.02166 | 0.002659 | -0.012 | 66.36097 | 3.8E-16 |
|  | rs4308051 | 0.015993 | 0.001819 | 0.01295 | 77.33316 | 1.4E-18 |
|  | rs62081464 | -0.00988 | 0.00178 | -0.00818 | 30.83285 | 2.8E-08 |
|  | rs143002906 | 0.026228 | 0.004574 | 0.008444 | 32.8777 | 9.8E-09 |
|  | rs35054365 | 0.012807 | 0.001497 | 0.012597 | 73.1746 | 1.2E-17 |
|  | rs10403906 | -0.01003 | 0.001487 | -0.00994 | 45.53901 | 1.5E-11 |
|  | rs11669079 | 0.010954 | 0.001632 | 0.009887 | 45.06984 | 1.9E-11 |
|  | rs8101782 | 0.009551 | 0.001722 | 0.00817 | 30.77383 | 2.9E-08 |
|  | rs8108461 | 0.009524 | 0.001507 | 0.00931 | 39.96101 | 2.6E-10 |
|  | rs143384 | 0.02092 | 0.001512 | 0.020379 | 191.5418 | 1.5E-43 |
|  | rs4811040 | -0.00925 | 0.001669 | -0.00816 | 30.71611 | 3E-08 |
|  | rs9611273 | 0.010789 | 0.00173 | 0.009182 | 38.86987 | 4.5E-10 |
|  | rs6006984 | 0.009849 | 0.001654 | 0.008769 | 35.45357 | 2.6E-09 |
| Hand grip strength (right) |  |  |  |  |  |  |
|  | rs58670122 | -0.01322 | 0.002148 | -0.00907 | 37.90271 | 7.4E-10 |
|  | rs10798876 | 0.008795 | 0.001498 | 0.008646 | 34.47063 | 4.3E-09 |
|  | rs10798483 | 0.014508 | 0.001495 | 0.014293 | 94.21733 | 2.8E-22 |
|  | rs6693965 | -0.01625 | 0.00223 | -0.01073 | 53.09113 | 3.2E-13 |
|  | rs4927015 | 0.013059 | 0.001509 | 0.012744 | 74.89799 | 5E-18 |
|  | rs1952256 | 0.009768 | 0.001563 | 0.009202 | 39.04429 | 4.1E-10 |
|  | rs35304341 | -0.01429 | 0.002603 | -0.00808 | 30.12718 | 4E-08 |
|  | rs823130 | -0.01239 | 0.001503 | -0.01213 | 67.89588 | 1.7E-16 |
|  | rs56144131 | -0.01307 | 0.002087 | -0.00922 | 39.23266 | 3.8E-10 |
|  | rs7549184 | 0.01056 | 0.001814 | 0.008574 | 33.90091 | 5.8E-09 |
|  | rs10799428 | -0.01436 | 0.001904 | -0.01111 | 56.89223 | 4.6E-14 |
|  | rs12562146 | 0.011871 | 0.002116 | 0.008263 | 31.48494 | 2E-08 |
|  | rs2147461 | 0.01343 | 0.00231 | 0.00856 | 33.78809 | 6.1E-09 |
|  | rs4121165 | -0.01198 | 0.001821 | -0.00969 | 43.25491 | 4.8E-11 |
|  | rs6693567 | -0.00975 | 0.001678 | -0.00856 | 33.75204 | 6.3E-09 |
|  | rs1892425 | 0.011336 | 0.001757 | 0.0095 | 41.61258 | 1.1E-10 |
|  | rs150330307 | -0.03258 | 0.004224 | -0.01136 | 59.50246 | 1.2E-14 |
|  | rs1550115 | 0.015284 | 0.001713 | 0.01314 | 79.62662 | 4.5E-19 |
|  | rs1442883 | -0.01062 | 0.001714 | -0.00912 | 38.37123 | 5.8E-10 |
|  | rs6711390 | 0.013266 | 0.001529 | 0.012779 | 75.30451 | 4E-18 |
|  | rs35833641 | 0.009223 | 0.001602 | 0.008479 | 33.1534 | 8.5E-09 |
|  | rs1840753 | 0.017694 | 0.003121 | 0.008349 | 32.14458 | 1.4E-08 |
|  | rs1047891 | 0.009687 | 0.001597 | 0.008935 | 36.81407 | 1.3E-09 |
|  | rs2894602 | 0.010133 | 0.001768 | 0.008441 | 32.85767 | 9.9E-09 |
|  | rs7565148 | -0.01035 | 0.001489 | -0.01024 | 48.31268 | 3.6E-12 |
|  | rs7576964 | 0.009745 | 0.001569 | 0.009146 | 38.57521 | 5.3E-10 |
|  | rs34030812 | -0.00913 | 0.001543 | -0.00871 | 35.01596 | 3.3E-09 |
|  | rs7575451 | -0.0106 | 0.001556 | -0.01004 | 46.46645 | 9.3E-12 |
|  | rs12616285 | 0.01222 | 0.002094 | 0.008594 | 34.05416 | 5.4E-09 |
|  | rs6715064 | -0.00921 | 0.001606 | -0.00845 | 32.9116 | 9.6E-09 |
|  | rs1641457 | 0.013073 | 0.001785 | 0.010783 | 53.61328 | 2.4E-13 |
|  | rs3771498 | 0.014161 | 0.001489 | 0.014008 | 90.49498 | 1.9E-21 |
|  | rs12052508 | -0.01335 | 0.00228 | -0.00862 | 34.29525 | 4.7E-09 |
|  | rs10193039 | -0.01033 | 0.001652 | -0.00921 | 39.08019 | 4.1E-10 |
|  | rs2194747 | 0.009851 | 0.001639 | 0.00885 | 36.11546 | 1.9E-09 |
|  | rs6792762 | -0.00912 | 0.001516 | -0.00886 | 36.18894 | 1.8E-09 |
|  | rs7652177 | 0.008581 | 0.001487 | 0.008499 | 33.31008 | 7.9E-09 |
|  | rs2194411 | 0.014283 | 0.002248 | 0.009357 | 40.3712 | 2.1E-10 |
|  | rs1440152 | 0.008271 | 0.0015 | 0.008121 | 30.40874 | 3.5E-08 |
|  | rs2362972 | -0.00857 | 0.001508 | -0.00837 | 32.30027 | 1.3E-08 |
|  | rs62234790 | 0.010585 | 0.001727 | 0.009028 | 37.5851 | 8.8E-10 |
|  | rs35701422 | -0.00869 | 0.001535 | -0.00834 | 32.06648 | 1.5E-08 |
|  | rs2341184 | 0.010061 | 0.001671 | 0.008865 | 36.23806 | 1.7E-09 |
|  | rs35457492 | 0.008339 | 0.001491 | 0.008236 | 31.27792 | 2.2E-08 |
|  | rs9757079 | 0.009802 | 0.001597 | 0.009039 | 37.67432 | 8.4E-10 |
|  | rs71298370 | 0.016599 | 0.002708 | 0.009025 | 37.56124 | 8.9E-10 |
|  | rs9853018 | 0.010198 | 0.001494 | 0.01005 | 46.57492 | 8.8E-12 |
|  | rs34587452 | -0.01109 | 0.00181 | -0.00902 | 37.5108 | 9.1E-10 |
|  | rs7657558 | 0.010726 | 0.001662 | 0.009502 | 41.63195 | 1.1E-10 |
|  | rs114924396 | -0.01913 | 0.003318 | -0.00849 | 33.22721 | 8.2E-09 |
|  | rs13146142 | -0.0208 | 0.002032 | -0.01507 | 104.7841 | 1.4E-24 |
|  | rs13106087 | 0.012926 | 0.001977 | 0.009628 | 42.74726 | 6.2E-11 |
|  | rs997850 | -0.00902 | 0.00153 | -0.00868 | 34.7659 | 3.7E-09 |
|  | rs13107325 | -0.0275 | 0.002825 | -0.01434 | 94.77551 | 2.1E-22 |
|  | rs13169333 | 0.009291 | 0.001696 | 0.008066 | 30.0002 | 4.3E-08 |
|  | rs75457267 | -0.01873 | 0.003395 | -0.00813 | 30.44286 | 3.4E-08 |
|  | rs12522139 | -0.01144 | 0.001973 | -0.00854 | 33.59795 | 6.8E-09 |
|  | rs6882168 | -0.00909 | 0.001576 | -0.0085 | 33.28786 | 7.9E-09 |
|  | rs13356200 | -0.00921 | 0.001531 | -0.00886 | 36.1908 | 1.8E-09 |
|  | rs6870324 | -0.00999 | 0.001683 | -0.00874 | 35.19691 | 3E-09 |
|  | rs13355365 | -0.00847 | 0.001534 | -0.00813 | 30.45939 | 3.4E-08 |
|  | rs4868110 | -0.00971 | 0.00159 | -0.00899 | 37.30867 | 1E-09 |
|  | rs2431112 | -0.01117 | 0.001497 | -0.01099 | 55.66774 | 8.6E-14 |
|  | rs2631360 | -0.01118 | 0.001486 | -0.01108 | 56.57034 | 5.4E-14 |
|  | rs2322754 | -0.0117 | 0.001985 | -0.00868 | 34.74143 | 3.8E-09 |
|  | rs9322822 | 0.01108 | 0.001593 | 0.010245 | 48.39779 | 3.5E-12 |
|  | rs9388051 | 0.010626 | 0.001912 | 0.008186 | 30.90025 | 2.7E-08 |
|  | rs7451021 | -0.0158 | 0.001606 | -0.01449 | 96.78046 | 7.7E-23 |
|  | rs113835839 | -0.00986 | 0.001725 | -0.00842 | 32.7007 | 1.1E-08 |
|  | rs11243202 | 0.011638 | 0.001493 | 0.011478 | 60.75736 | 6.5E-15 |
|  | rs645144 | -0.00869 | 0.001586 | -0.00807 | 29.99704 | 4.3E-08 |
|  | rs721101 | 0.009467 | 0.001674 | 0.008327 | 31.97476 | 1.6E-08 |
|  | rs77485342 | 0.035337 | 0.00559 | 0.00931 | 39.96448 | 2.6E-10 |
|  | rs9267806 | -0.01674 | 0.001702 | -0.01449 | 96.77953 | 7.7E-23 |
|  | rs185320691 | -0.02025 | 0.002693 | -0.01107 | 56.51665 | 5.6E-14 |
|  | rs113315602 | -0.02129 | 0.002668 | -0.01175 | 63.70177 | 1.4E-15 |
|  | rs1125 | -0.01003 | 0.001575 | -0.00938 | 40.57232 | 1.9E-10 |
|  | rs9396861 | -0.00963 | 0.001553 | -0.00913 | 38.42568 | 5.7E-10 |
|  | rs35175534 | -0.01918 | 0.002358 | -0.01198 | 66.16161 | 4.2E-16 |
|  | rs1885690 | -0.0084 | 0.001512 | -0.00818 | 30.85665 | 2.8E-08 |
|  | rs852520 | -0.0089 | 0.001573 | -0.00833 | 32.03433 | 1.5E-08 |
|  | rs4549685 | 0.009715 | 0.001581 | 0.009048 | 37.74719 | 8.1E-10 |
|  | rs112330055 | 0.017982 | 0.00319 | 0.008301 | 31.77568 | 1.7E-08 |
|  | rs7790322 | -0.0086 | 0.001509 | -0.00839 | 32.49431 | 1.2E-08 |
|  | rs6962338 | -0.02035 | 0.003623 | -0.00827 | 31.53914 | 2E-08 |
|  | rs6977081 | 0.01286 | 0.001589 | 0.01192 | 65.52613 | 5.7E-16 |
|  | rs2389763 | -0.00833 | 0.001514 | -0.0081 | 30.26261 | 3.8E-08 |
|  | rs2717351 | 0.01277 | 0.001835 | 0.010249 | 48.43992 | 3.4E-12 |
|  | rs9639938 | 0.00873 | 0.001495 | 0.008601 | 34.1109 | 5.2E-09 |
|  | rs10278546 | 0.010754 | 0.001884 | 0.008407 | 32.59077 | 1.1E-08 |
|  | rs4730984 | 0.010479 | 0.001742 | 0.008856 | 36.16685 | 1.8E-09 |
|  | rs4737446 | 0.010267 | 0.001621 | 0.009325 | 40.09942 | 2.4E-10 |
|  | rs62509875 | -0.01318 | 0.001973 | -0.00984 | 44.61218 | 2.4E-11 |
|  | rs1486925 | -0.00953 | 0.001605 | -0.00874 | 35.26184 | 2.9E-09 |
|  | rs6473015 | 0.0096 | 0.001646 | 0.008587 | 34.00122 | 5.5E-09 |
|  | rs7871404 | 0.011935 | 0.001901 | 0.009246 | 39.42421 | 3.4E-10 |
|  | rs116922558 | -0.02463 | 0.00386 | -0.00939 | 40.69506 | 1.8E-10 |
|  | rs2208562 | -0.01156 | 0.001522 | -0.01118 | 57.66922 | 3.1E-14 |
|  | rs7034200 | 0.008689 | 0.001489 | 0.008595 | 34.06199 | 5.3E-09 |
|  | rs113851275 | 0.013143 | 0.002398 | 0.00807 | 30.03056 | 4.3E-08 |
|  | rs11998884 | 0.017343 | 0.003124 | 0.008175 | 30.81471 | 2.8E-08 |
|  | rs600038 | -0.01038 | 0.001834 | -0.00833 | 32.02348 | 1.5E-08 |
|  | rs10761411 | -0.0115 | 0.002021 | -0.00838 | 32.37066 | 1.3E-08 |
|  | rs72820369 | 0.016127 | 0.00233 | 0.010194 | 47.92026 | 4.4E-12 |
|  | rs4751671 | 0.008233 | 0.0015 | 0.008082 | 30.11929 | 4.1E-08 |
|  | rs4752689 | 0.008753 | 0.001508 | 0.00855 | 33.70899 | 6.4E-09 |
|  | rs12412806 | -0.0091 | 0.001641 | -0.00816 | 30.70861 | 3E-08 |
|  | rs2273555 | 0.011032 | 0.001522 | 0.010677 | 52.56595 | 4.2E-13 |
|  | rs4962700 | 0.009327 | 0.001637 | 0.008389 | 32.45127 | 1.2E-08 |
|  | rs1556659 | 0.017535 | 0.001537 | 0.0168 | 130.1671 | 3.8E-30 |
|  | rs12763284 | 0.009597 | 0.001489 | 0.00949 | 41.52937 | 1.2E-10 |
|  | rs11022513 | -0.00921 | 0.00151 | -0.00898 | 37.1984 | 1.1E-09 |
|  | rs11039348 | -0.00977 | 0.001561 | -0.00922 | 39.15905 | 3.9E-10 |
|  | rs2244621 | 0.011596 | 0.002119 | 0.008057 | 29.93536 | 4.5E-08 |
|  | rs61389091 | 0.022055 | 0.003738 | 0.008689 | 34.81683 | 3.6E-09 |
|  | rs34845616 | 0.009795 | 0.001736 | 0.008308 | 31.82619 | 1.7E-08 |
|  | rs12790261 | -0.02637 | 0.002707 | -0.01434 | 94.88133 | 2E-22 |
|  | rs72977282 | -0.01681 | 0.001514 | -0.01635 | 123.2905 | 1.2E-28 |
|  | rs6592737 | -0.00925 | 0.001541 | -0.00884 | 36.00273 | 2E-09 |
|  | rs10770125 | 0.008441 | 0.001488 | 0.008356 | 32.19295 | 1.4E-08 |
|  | rs1635527 | 0.009753 | 0.001495 | 0.009607 | 42.56144 | 6.8E-11 |
|  | rs76895963 | 0.035977 | 0.005757 | 0.009203 | 39.05444 | 4.1E-10 |
|  | rs7301953 | -0.01154 | 0.001604 | -0.01059 | 51.72673 | 6.4E-13 |
|  | rs10846071 | -0.01566 | 0.001519 | -0.01518 | 106.2473 | 6.5E-25 |
|  | rs12823922 | -0.01142 | 0.001786 | -0.00941 | 40.86522 | 1.6E-10 |
|  | rs10784502 | -0.01112 | 0.001486 | -0.01102 | 56.03924 | 7.1E-14 |
|  | rs7963801 | -0.01127 | 0.00151 | -0.01099 | 55.70317 | 8.4E-14 |
|  | rs12316046 | -0.0162 | 0.001532 | -0.01557 | 111.8642 | 3.8E-26 |
|  | rs4768725 | 0.009059 | 0.001621 | 0.008231 | 31.23853 | 2.3E-08 |
|  | rs7953280 | -0.00899 | 0.001494 | -0.00886 | 36.16199 | 1.8E-09 |
|  | rs3118914 | -0.01944 | 0.001808 | -0.01583 | 115.5646 | 5.9E-27 |
|  | rs2296316 | -0.00821 | 0.001502 | -0.00805 | 29.89672 | 4.6E-08 |
|  | rs12889267 | -0.01229 | 0.00199 | -0.00909 | 38.11003 | 6.7E-10 |
|  | rs935728 | 0.009553 | 0.001587 | 0.008864 | 36.22846 | 1.8E-09 |
|  | rs7148603 | 0.009306 | 0.001588 | 0.008628 | 34.32692 | 4.7E-09 |
|  | rs10483727 | -0.00902 | 0.001525 | -0.00871 | 34.98922 | 3.3E-09 |
|  | rs9652468 | -0.01253 | 0.001721 | -0.01072 | 53.00356 | 3.3E-13 |
|  | rs2871865 | -0.02369 | 0.00232 | -0.01503 | 104.2164 | 1.8E-24 |
|  | rs2165241 | 0.012249 | 0.001488 | 0.012118 | 67.71895 | 1.9E-16 |
|  | rs4553566 | -0.0093 | 0.001492 | -0.00918 | 38.85335 | 4.6E-10 |
|  | rs12914702 | 0.010779 | 0.001693 | 0.009377 | 40.54332 | 1.9E-10 |
|  | rs12101479 | -0.01061 | 0.001755 | -0.0089 | 36.55516 | 1.5E-09 |
|  | rs12899474 | -0.01489 | 0.002399 | -0.00914 | 38.5324 | 5.4E-10 |
|  | rs246181 | 0.009704 | 0.001549 | 0.009228 | 39.2712 | 3.7E-10 |
|  | rs11642954 | -0.01319 | 0.001879 | -0.01034 | 49.26959 | 2.2E-12 |
|  | rs7196917 | -0.01058 | 0.001503 | -0.01037 | 49.57842 | 1.9E-12 |
|  | rs8055199 | -0.00896 | 0.001573 | -0.00839 | 32.47676 | 1.2E-08 |
|  | rs7206195 | -0.01532 | 0.001939 | -0.01163 | 62.419 | 2.8E-15 |
|  | rs248831 | 0.00989 | 0.001717 | 0.008483 | 33.18281 | 8.4E-09 |
|  | rs62037412 | 0.00933 | 0.001554 | 0.00884 | 36.03444 | 1.9E-09 |
|  | rs4785574 | -0.01038 | 0.0015 | -0.01019 | 47.88717 | 4.5E-12 |
|  | rs3848369 | -0.00954 | 0.001531 | -0.00917 | 38.79671 | 4.7E-10 |
|  | rs76749769 | 0.014432 | 0.00258 | 0.008239 | 31.29885 | 2.2E-08 |
|  | rs4784329 | -0.01334 | 0.001507 | -0.01303 | 78.2725 | 9E-19 |
|  | rs7214252 | -0.01024 | 0.001825 | -0.00826 | 31.49764 | 2E-08 |
|  | rs2854152 | 0.010991 | 0.001598 | 0.010131 | 47.32723 | 6E-12 |
|  | rs1043515 | 0.013828 | 0.001498 | 0.013589 | 85.15754 | 2.8E-20 |
|  | rs12452505 | -0.01443 | 0.002135 | -0.00995 | 45.67011 | 1.4E-11 |
|  | rs2587505 | -0.00927 | 0.001509 | -0.00905 | 37.74702 | 8.1E-10 |
|  | rs4793658 | -0.01367 | 0.002486 | -0.0081 | 30.23642 | 3.8E-08 |
|  | rs56074046 | -0.00904 | 0.00154 | -0.00864 | 34.44104 | 4.4E-09 |
|  | rs56365901 | -0.01426 | 0.001793 | -0.01171 | 63.27352 | 1.8E-15 |
|  | rs10520770 | 0.011742 | 0.001498 | 0.011545 | 61.46347 | 4.5E-15 |
|  | rs635538 | -0.02199 | 0.002663 | -0.01216 | 68.22802 | 1.5E-16 |
|  | rs4369779 | 0.017203 | 0.001821 | 0.013914 | 89.27732 | 3.4E-21 |
|  | rs34217742 | 0.014625 | 0.002278 | 0.009455 | 41.2229 | 1.4E-10 |
|  | rs7249 | 0.008482 | 0.001544 | 0.008088 | 30.16139 | 4E-08 |
|  | rs4802848 | 0.011077 | 0.001675 | 0.009739 | 43.73327 | 3.8E-11 |
|  | rs36065733 | 0.00972 | 0.001496 | 0.009568 | 42.21672 | 8.2E-11 |
|  | rs79723785 | -0.03405 | 0.006029 | -0.00832 | 31.89973 | 1.6E-08 |
|  | rs7266065 | 0.009939 | 0.001595 | 0.009177 | 38.83747 | 4.6E-10 |
|  | rs911642 | 0.00863 | 0.001538 | 0.008264 | 31.49205 | 2E-08 |
|  | rs6063504 | 0.008623 | 0.001494 | 0.008501 | 33.32554 | 7.8E-09 |
|  | rs143384 | 0.023042 | 0.001514 | 0.022413 | 231.7368 | 2.5E-52 |
|  | rs2226685 | 0.010333 | 0.001743 | 0.008729 | 35.13692 | 3.1E-09 |
|  | rs6006984 | 0.010299 | 0.001657 | 0.009155 | 38.64589 | 5.1E-10 |
| Low hand grip strength  (60 years and older) | | |  |  |  |  |
|  | rs12140813 | 0.0511 | 0.0094 | 0.010733 | 29.55195 | 4.76E-08 |
|  | rs958685 | -0.0428 | 0.0074 | -0.01142 | 33.45215 | 6.52E-09 |
|  | rs7624084 | -0.0428 | 0.0074 | -0.01142 | 33.45215 | 8.51E-09 |
|  | rs13107325 | 0.0897 | 0.0138 | 0.012833 | 42.25 | 7.42E-11 |
|  | rs34415150 | 0.0833 | 0.0099 | 0.016611 | 70.79778 | 4.42E-17 |
|  | rs185320691 | 0.0913 | 0.0146 | 0.012346 | 39.10532 | 3.84E-10 |
|  | rs10952289 | -0.0435 | 0.0078 | -0.01101 | 31.10207 | 2.1E-08 |
|  | rs11236213 | -0.0504 | 0.008 | -0.01244 | 39.69 | 3.01E-10 |
|  | rs10846071 | 0.0433 | 0.0075 | 0.011398 | 33.33138 | 7.32E-09 |
|  | rs34464763 | 0.0544 | 0.0086 | 0.012488 | 40.01298 | 3.15E-10 |
|  | rs3118903 | 0.0575 | 0.0088 | 0.0129 | 42.69434 | 6.71E-11 |
|  | rs2899611 | 0.0431 | 0.0074 | 0.011499 | 33.92275 | 6.01E-09 |
|  | rs8061064 | 0.0407 | 0.0074 | 0.010859 | 30.25 | 3.55E-08 |
|  | rs143459567 | 0.1185 | 0.0189 | 0.012378 | 39.31091 | 3.41E-10 |
|  | rs62102286 | -0.0487 | 0.0074 | -0.01299 | 43.31063 | 5.49E-11 |
|  | rs79723785 | 0.1674 | 0.0293 | 0.01128 | 32.64192 | 1.16E-08 |
|  | rs143384 | -0.0545 | 0.0075 | -0.01435 | 52.80444 | 4.47E-13 |
| Appendicular lean mass |  |  |  |  |  |  |
|  | rs200439 | -0.0128 | 0.0023 | -0.00829 | 30.97164 | 1.51E-08 |
|  | rs2807339 | 0.0162 | 0.0022 | 0.010973 | 54.22314 | 1.24E-13 |
|  | rs150188352 | 0.0305 | 0.0024 | 0.018936 | 161.5017 | 8.43E-38 |
|  | rs60804050 | -0.0217 | 0.0021 | -0.0154 | 106.7778 | 5.01E-24 |
|  | rs2025808 | 0.0122 | 0.0022 | 0.008264 | 30.75207 | 1.72E-08 |
|  | rs6693481 | -0.0143 | 0.002 | -0.01066 | 51.1225 | 2.21E-12 |
|  | rs61827272 | 0.0144 | 0.0021 | 0.010219 | 47.02041 | 7.89E-12 |
|  | rs12724708 | 0.0243 | 0.002 | 0.018104 | 147.6225 | 1.66E-35 |
|  | rs3033487 | 0.0153 | 0.0024 | 0.0095 | 40.64063 | 1.93E-10 |
|  | rs11260623 | 0.0117 | 0.0019 | 0.009177 | 37.91967 | 4.86E-10 |
|  | rs301807 | -0.0144 | 0.0019 | -0.01129 | 57.44044 | 2.53E-14 |
|  | rs212526 | 0.0214 | 0.0019 | 0.016783 | 126.8587 | 3.84E-29 |
|  | rs7522400 | 0.0129 | 0.0022 | 0.008738 | 34.38223 | 5.62E-09 |
|  | rs670318 | 0.0413 | 0.0044 | 0.013987 | 88.10382 | 2.52E-21 |
|  | rs2025609 | 0.0186 | 0.0026 | 0.010661 | 51.17751 | 2E-12 |
|  | rs7543202 | 0.0129 | 0.0019 | 0.010118 | 46.09695 | 2.86E-11 |
|  | rs11590254 | 0.0186 | 0.002 | 0.013859 | 86.49 | 4.34E-20 |
|  | rs34654458 | -0.0191 | 0.0019 | -0.01498 | 101.0554 | 5.72E-24 |
|  | rs4644481 | -0.0112 | 0.0019 | -0.00878 | 34.74792 | 3.54E-09 |
|  | rs7367519 | 0.0164 | 0.002 | 0.01222 | 67.24 | 4.68E-16 |
|  | rs951366 | 0.0205 | 0.0019 | 0.016078 | 116.4127 | 9.15E-27 |
|  | rs7418410 | 0.0155 | 0.0019 | 0.012157 | 66.55125 | 5.64E-16 |
|  | rs7543136 | -0.021 | 0.0021 | -0.0149 | 100 | 9.96E-24 |
|  | rs4360494 | -0.0198 | 0.0019 | -0.01553 | 108.5983 | 7.88E-26 |
|  | rs55717234 | 0.0122 | 0.0019 | 0.009569 | 41.22992 | 1.34E-10 |
|  | rs11580040 | 0.0325 | 0.0035 | 0.013837 | 86.22449 | 6.76E-21 |
|  | rs2209098 | 0.024 | 0.002 | 0.017881 | 144 | 1.73E-32 |
|  | rs17278379 | 0.0226 | 0.0029 | 0.011613 | 60.73246 | 2.4E-15 |
|  | rs66579625 | 0.0178 | 0.0019 | 0.013961 | 87.76731 | 8.91E-21 |
|  | rs4655345 | -0.0246 | 0.0019 | -0.01929 | 167.6343 | 5.81E-38 |
|  | rs1797070 | 0.0219 | 0.0021 | 0.01554 | 108.7551 | 4.93E-25 |
|  | rs12563442 | 0.0122 | 0.0021 | 0.008658 | 33.75057 | 9.93E-09 |
|  | rs80295797 | -0.0198 | 0.002 | -0.01475 | 98.01 | 3.83E-23 |
|  | rs4274112 | -0.0217 | 0.002 | -0.01617 | 117.7225 | 2.47E-28 |
|  | rs113107560 | -0.019 | 0.0019 | -0.0149 | 100 | 6.99E-23 |
|  | rs11210892 | 0.0118 | 0.002 | 0.008793 | 34.81 | 3.57E-09 |
|  | rs12074850 | 0.0393 | 0.0033 | 0.017745 | 141.8264 | 2.72E-33 |
|  | rs1514134 | -0.0114 | 0.0019 | -0.00894 | 36 | 3.57E-09 |
|  | rs34517439 | 0.0421 | 0.0029 | 0.02163 | 210.7503 | 5.8E-48 |
|  | rs10922475 | 0.0159 | 0.0019 | 0.012471 | 70.03047 | 2.17E-17 |
|  | rs3768495 | -0.0178 | 0.0021 | -0.01263 | 71.8458 | 1.07E-17 |
|  | rs28736838 | -0.0117 | 0.002 | -0.00872 | 34.2225 | 1.07E-08 |
|  | rs200091076 | -0.0117 | 0.002 | -0.00872 | 34.2225 | 6.92E-09 |
|  | rs905938 | 0.0394 | 0.0021 | 0.02795 | 352.0091 | 8.43E-77 |
|  | rs6675858 | -0.0137 | 0.0023 | -0.00888 | 35.48015 | 2.4E-09 |
|  | rs2789365 | -0.0145 | 0.0019 | -0.01137 | 58.241 | 1.14E-14 |
|  | rs377599 | 0.0217 | 0.0019 | 0.017018 | 130.4404 | 3.5E-29 |
|  | rs11121615 | -0.0202 | 0.002 | -0.01505 | 102.01 | 3.32E-23 |
|  | rs2791654 | -0.0239 | 0.0022 | -0.01619 | 118.0186 | 1.21E-28 |
|  | rs6425817 | 0.0157 | 0.002 | 0.011698 | 61.6225 | 2.98E-15 |
|  | rs2885697 | -0.0323 | 0.002 | -0.02406 | 260.8225 | 9.21E-60 |
|  | rs4847378 | 0.0136 | 0.0019 | 0.010667 | 51.23546 | 1.64E-12 |
|  | rs1405227 | 0.0129 | 0.002 | 0.009612 | 41.6025 | 1.57E-10 |
|  | rs1325596 | 0.0287 | 0.0019 | 0.022506 | 228.169 | 2.77E-52 |
|  | rs200348453 | 0.018 | 0.0019 | 0.014117 | 89.75069 | 7.88E-21 |
|  | rs234640 | -0.0131 | 0.0019 | -0.01027 | 47.5374 | 3.87E-12 |
|  | rs1005723 | 0.0161 | 0.0024 | 0.009997 | 45.00174 | 1.79E-11 |
|  | rs10171272 | 0.0136 | 0.002 | 0.010134 | 46.24 | 2.87E-11 |
|  | rs17681189 | -0.0131 | 0.0019 | -0.01027 | 47.5374 | 5.82E-12 |
|  | rs76517946 | -0.0368 | 0.0035 | -0.01567 | 110.5502 | 1.69E-26 |
|  | rs867529 | 0.0184 | 0.0021 | 0.013057 | 76.77098 | 1E-18 |
|  | rs6543146 | 0.0154 | 0.0019 | 0.012078 | 65.69529 | 4.19E-16 |
|  | rs6738207 | 0.0127 | 0.0019 | 0.009961 | 44.67867 | 4.14E-11 |
|  | rs71414738 | 0.015 | 0.0025 | 0.008942 | 36 | 1E-09 |
|  | rs2390669 | 0.0174 | 0.0028 | 0.009261 | 38.61735 | 7E-10 |
|  | rs13430869 | 0.0272 | 0.0021 | 0.0193 | 167.7642 | 6.37E-37 |
|  | rs17408561 | 0.0123 | 0.002 | 0.009165 | 37.8225 | 6E-10 |
|  | rs17246129 | 0.0254 | 0.002 | 0.018924 | 161.29 | 1.27E-35 |
|  | rs2305141 | 0.0183 | 0.0019 | 0.014353 | 92.76731 | 1.07E-21 |
|  | rs2971857 | -0.0119 | 0.0019 | -0.00933 | 39.22715 | 3.7E-10 |
|  | rs10203320 | 0.0138 | 0.002 | 0.010283 | 47.61 | 7.86E-12 |
|  | rs144627572 | 0.0439 | 0.0053 | 0.012343 | 68.6084 | 1.3E-16 |
|  | rs1260326 | 0.0323 | 0.0019 | 0.025327 | 289 | 6.16E-64 |
|  | rs650508 | -0.013 | 0.002 | -0.00969 | 42.25 | 1.86E-10 |
|  | rs60142646 | -0.0237 | 0.0042 | -0.00841 | 31.84184 | 2.49E-08 |
|  | rs75022676 | -0.0163 | 0.0023 | -0.01056 | 50.22495 | 2.84E-12 |
|  | rs199647708 | 0.0114 | 0.0019 | 0.008942 | 36 | 3.43E-09 |
|  | rs17400325 | 0.0345 | 0.0047 | 0.010939 | 53.88185 | 2.1E-13 |
|  | rs1035583 | 0.0148 | 0.0019 | 0.011608 | 60.6759 | 1.99E-14 |
|  | rs7598430 | -0.016 | 0.0019 | -0.01255 | 70.91413 | 1.37E-17 |
|  | rs10202701 | 0.0227 | 0.0019 | 0.017802 | 142.7396 | 3.11E-33 |
|  | rs10205141 | 0.0241 | 0.0044 | 0.008163 | 30.00052 | 4.71E-08 |
|  | rs10203386 | -0.0238 | 0.0019 | -0.01866 | 156.9086 | 1.75E-36 |
|  | rs10202845 | -0.0288 | 0.003 | -0.01431 | 92.16 | 5.35E-22 |
|  | rs67716382 | 0.0226 | 0.0023 | 0.014642 | 96.55198 | 1.65E-23 |
|  | rs2717008 | -0.0127 | 0.0019 | -0.00996 | 44.67867 | 4.99E-11 |
|  | rs4852257 | -0.0231 | 0.0019 | -0.01812 | 147.8144 | 6.21E-34 |
|  | rs201570119 | 0.0194 | 0.0023 | 0.012569 | 71.14556 | 5.48E-17 |
|  | rs35223841 | 0.0106 | 0.0019 | 0.008314 | 31.12465 | 4.25E-08 |
|  | rs55852614 | -0.0393 | 0.0022 | -0.02661 | 319.1095 | 3.29E-73 |
|  | rs3063063 | -0.0182 | 0.002 | -0.01356 | 82.81 | 2.57E-19 |
|  | rs144343497 | -0.0134 | 0.0023 | -0.00868 | 33.94329 | 5.06E-09 |
|  | rs700677 | 0.0173 | 0.002 | 0.01289 | 74.8225 | 1.13E-18 |
|  | rs12997625 | -0.017 | 0.0019 | -0.01333 | 80.0554 | 1.5E-19 |
|  | rs3116194 | -0.0295 | 0.0032 | -0.01374 | 84.98535 | 8.29E-21 |
|  | rs7570235 | -0.0168 | 0.0019 | -0.01318 | 78.18283 | 2.08E-18 |
|  | rs7563362 | 0.0352 | 0.0027 | 0.019426 | 169.9643 | 3.27E-39 |
|  | rs6721191 | -0.0144 | 0.0019 | -0.01129 | 57.44044 | 3.17E-14 |
|  | rs12713004 | 0.0367 | 0.0021 | 0.026036 | 305.4172 | 2.4E-68 |
|  | rs6739278 | -0.021 | 0.0024 | -0.01304 | 76.5625 | 1.27E-18 |
|  | rs2347603 | -0.0181 | 0.0022 | -0.01226 | 67.68802 | 5.65E-17 |
|  | rs702886 | 0.012 | 0.002 | 0.008942 | 36 | 1.11E-09 |
|  | rs55980611 | 0.0164 | 0.0028 | 0.008729 | 34.30612 | 4.22E-09 |
|  | rs14976 | 0.0144 | 0.002 | 0.01073 | 51.84 | 1.43E-12 |
|  | rs72809820 | -0.0111 | 0.002 | -0.00827 | 30.8025 | 3.13E-08 |
|  | rs9636364 | 0.011 | 0.0019 | 0.008628 | 33.51801 | 5.09E-09 |
|  | rs10864899 | -0.0112 | 0.0019 | -0.00878 | 34.74792 | 3.85E-09 |
|  | rs488621 | 0.0191 | 0.0019 | 0.01498 | 101.0554 | 2.86E-24 |
|  | rs2138374 | -0.0149 | 0.002 | -0.0111 | 55.5025 | 2.79E-13 |
|  | rs10221831 | 0.03 | 0.0053 | 0.008435 | 32.03987 | 1.8E-08 |
|  | rs17773965 | -0.0163 | 0.0027 | -0.009 | 36.44582 | 1.51E-09 |
|  | rs62106258 | -0.0504 | 0.0044 | -0.01707 | 131.2066 | 6.45E-31 |
|  | rs3769598 | 0.0171 | 0.0027 | 0.009438 | 40.11111 | 1.32E-10 |
|  | rs202098543 | 0.0132 | 0.002 | 0.009836 | 43.56 | 1.56E-11 |
|  | rs59985551 | -0.0313 | 0.0022 | -0.0212 | 202.4153 | 2.43E-44 |
|  | rs62143873 | -0.0115 | 0.0019 | -0.00902 | 36.63435 | 1.19E-09 |
|  | rs12616192 | -0.0261 | 0.0038 | -0.01024 | 47.17521 | 6.83E-12 |
|  | rs61397287 | 0.0235 | 0.0036 | 0.009728 | 42.61188 | 4.45E-11 |
|  | rs13391980 | -0.0225 | 0.0029 | -0.01156 | 60.1962 | 7.6E-15 |
|  | rs34788019 | -0.0139 | 0.0019 | -0.0109 | 53.52078 | 2.84E-13 |
|  | rs1047891 | 0.0233 | 0.002 | 0.01736 | 135.7225 | 5.7E-31 |
|  | rs1478575 | 0.0312 | 0.002 | 0.023243 | 243.36 | 5.2E-54 |
|  | rs11684531 | -0.0172 | 0.0028 | -0.00915 | 37.73469 | 4.17E-10 |
|  | rs1899040 | 0.0152 | 0.0023 | 0.009849 | 43.67486 | 9.04E-11 |
|  | rs2270894 | -0.0332 | 0.0024 | -0.02061 | 191.3611 | 1.25E-42 |
|  | rs113671109 | -0.015 | 0.0023 | -0.00972 | 42.53308 | 4.23E-11 |
|  | rs6789000 | 0.0121 | 0.002 | 0.009016 | 36.6025 | 1.61E-09 |
|  | rs4504126 | 0.046 | 0.0058 | 0.011819 | 62.90131 | 1.62E-15 |
|  | rs140440099 | 0.0613 | 0.0063 | 0.014499 | 94.67599 | 1.45E-22 |
|  | rs17718736 | 0.0115 | 0.002 | 0.008569 | 33.0625 | 1.39E-08 |
|  | rs4682483 | -0.0165 | 0.0026 | -0.00946 | 40.27367 | 2.65E-10 |
|  | rs4683435 | 0.0144 | 0.0022 | 0.009754 | 42.84298 | 1.6E-10 |
|  | rs900399 | 0.0164 | 0.0019 | 0.012863 | 74.50416 | 1.35E-17 |
|  | rs1290786 | -0.0143 | 0.0019 | -0.01122 | 56.64543 | 7.14E-14 |
|  | rs9647379 | 0.0215 | 0.0019 | 0.016862 | 128.0471 | 5.55E-29 |
|  | rs2194411 | 0.0443 | 0.0029 | 0.02276 | 233.352 | 2.43E-54 |
|  | rs11720869 | 0.0141 | 0.002 | 0.010506 | 49.7025 | 2.54E-12 |
|  | rs73052033 | -0.0151 | 0.0024 | -0.00938 | 39.58507 | 4.79E-10 |
|  | rs336630 | -0.0106 | 0.0019 | -0.00831 | 31.12465 | 2.9E-08 |
|  | rs9838614 | -0.0185 | 0.0019 | -0.01451 | 94.80609 | 1.21E-21 |
|  | rs6762851 | -0.0218 | 0.002 | -0.01624 | 118.81 | 1.5E-28 |
|  | rs182798714 | 0.0376 | 0.0062 | 0.009038 | 36.77836 | 1.45E-09 |
|  | rs591668 | -0.0174 | 0.0019 | -0.01365 | 83.86704 | 2E-19 |
|  | rs36012032 | 0.0298 | 0.0033 | 0.013457 | 81.54637 | 9.93E-20 |
|  | rs839255 | -0.0126 | 0.0021 | -0.00894 | 36 | 8.38E-10 |
|  | rs9809116 | -0.016 | 0.0019 | -0.01255 | 70.91413 | 1.31E-16 |
|  | rs7633464 | 0.0175 | 0.0019 | 0.013725 | 84.8338 | 1.27E-20 |
|  | rs115010283 | 0.034 | 0.002 | 0.025327 | 289 | 2.35E-63 |
|  | rs61732778 | 0.023 | 0.0037 | 0.009264 | 38.64134 | 3.13E-10 |
|  | rs4076108 | 0.0174 | 0.0022 | 0.011786 | 62.55372 | 2.3E-15 |
|  | rs34312629 | -0.017 | 0.0021 | -0.01206 | 65.53288 | 2.12E-15 |
|  | rs200739311 | -0.0128 | 0.002 | -0.00954 | 40.96 | 1.55E-10 |
|  | rs544136 | 0.0121 | 0.0022 | 0.008196 | 30.25 | 2.5E-08 |
|  | rs4073154 | 0.0274 | 0.0023 | 0.017751 | 141.9206 | 1.92E-33 |
|  | rs2871960 | 0.0469 | 0.0019 | 0.036762 | 609.3102 | 2.2E-135 |
|  | rs1730028 | 0.0131 | 0.0019 | 0.010275 | 47.5374 | 7.39E-12 |
|  | rs71635721 | 0.0316 | 0.0039 | 0.012074 | 65.65155 | 3.46E-16 |
|  | rs34390533 | -0.0257 | 0.0022 | -0.01741 | 136.4649 | 6.69E-32 |
|  | rs7610055 | -0.0373 | 0.0029 | -0.01916 | 165.4328 | 3.55E-38 |
|  | rs56239180 | -0.0459 | 0.0062 | -0.01103 | 54.80775 | 1.85E-13 |
|  | rs9828525 | 0.0121 | 0.0019 | 0.009491 | 40.55679 | 2.51E-10 |
|  | rs116493405 | 0.0287 | 0.0042 | 0.010183 | 46.69444 | 9.52E-12 |
|  | rs9832919 | -0.0179 | 0.002 | -0.01334 | 80.1025 | 7.9E-20 |
|  | rs1823217 | -0.0181 | 0.002 | -0.01349 | 81.9025 | 4.06E-20 |
|  | rs11461979 | 0.0143 | 0.0022 | 0.009687 | 42.25 | 9.8E-11 |
|  | rs113289555 | -0.0206 | 0.0023 | -0.01335 | 80.21928 | 7.33E-20 |
|  | rs13127468 | -0.0123 | 0.0019 | -0.00965 | 41.90859 | 9.86E-11 |
|  | rs10005035 | -0.0175 | 0.0021 | -0.01242 | 69.44444 | 6.51E-17 |
|  | rs1472852 | -0.0638 | 0.0026 | -0.03655 | 602.1361 | 8.2E-135 |
|  | rs963317 | -0.0136 | 0.002 | -0.01013 | 46.24 | 1.03E-11 |
|  | rs781669 | 0.0164 | 0.0019 | 0.012863 | 74.50416 | 3.14E-18 |
|  | rs13103161 | -0.0284 | 0.0019 | -0.02227 | 223.4238 | 2.38E-48 |
|  | rs6849302 | 0.0155 | 0.0024 | 0.009624 | 41.71007 | 7.11E-11 |
|  | rs145126099 | -0.0126 | 0.0019 | -0.00988 | 43.97784 | 3.29E-11 |
|  | rs1443536 | 0.0218 | 0.0021 | 0.015469 | 107.7642 | 1.91E-26 |
|  | rs72657800 | -0.0219 | 0.0035 | -0.00932 | 39.15184 | 6.39E-10 |
|  | rs11098677 | -0.0263 | 0.0023 | -0.01704 | 130.7543 | 3.94E-30 |
|  | rs12512942 | -0.0162 | 0.002 | -0.01207 | 65.61 | 1.58E-16 |
|  | rs59950280 | -0.0254 | 0.002 | -0.01892 | 161.29 | 7.32E-36 |
|  | rs10019221 | -0.0124 | 0.0019 | -0.00973 | 42.5928 | 1.22E-10 |
|  | rs36052389 | 0.0132 | 0.0019 | 0.010353 | 48.26593 | 5.27E-12 |
|  | rs3103223 | 0.0126 | 0.0022 | 0.008535 | 32.80165 | 6.02E-09 |
|  | rs139921635 | 0.0385 | 0.0062 | 0.009254 | 38.56009 | 6.16E-10 |
|  | rs111612346 | -0.0138 | 0.002 | -0.01028 | 47.61 | 4.25E-12 |
|  | rs34548509 | 0.0118 | 0.002 | 0.008793 | 34.81 | 3.2E-09 |
|  | rs13123591 | 0.0185 | 0.002 | 0.013784 | 85.5625 | 2.35E-20 |
|  | rs6821305 | 0.0204 | 0.0019 | 0.015999 | 115.2798 | 3.15E-26 |
|  | rs72695791 | -0.0297 | 0.0051 | -0.00868 | 33.91349 | 4.58E-09 |
|  | rs2324154 | 0.015 | 0.0019 | 0.011765 | 62.32687 | 1.92E-15 |
|  | rs116339650 | -0.0175 | 0.0029 | -0.00899 | 36.41498 | 1.05E-09 |
|  | rs2303423 | 0.0168 | 0.003 | 0.008345 | 31.36 | 2.64E-08 |
|  | rs13109280 | 0.0131 | 0.002 | 0.009761 | 42.9025 | 9.15E-11 |
|  | rs116052377 | 0.0225 | 0.0035 | 0.00958 | 41.32653 | 7.89E-11 |
|  | rs7689420 | 0.0466 | 0.0025 | 0.027769 | 347.4496 | 1.5E-76 |
|  | rs11721522 | 0.0106 | 0.0019 | 0.008314 | 31.12465 | 4.03E-08 |
|  | rs73856768 | -0.0247 | 0.0035 | -0.01052 | 49.80327 | 1.55E-12 |
|  | rs111622870 | -0.0282 | 0.0044 | -0.00955 | 41.07645 | 1.86E-10 |
|  | rs190823861 | -0.0345 | 0.0045 | -0.01142 | 58.77778 | 2.09E-14 |
|  | rs148617731 | 0.041 | 0.0043 | 0.014209 | 90.91401 | 5.92E-22 |
|  | rs11727162 | -0.017 | 0.0019 | -0.01333 | 80.0554 | 2.15E-19 |
|  | rs2035901 | 0.024 | 0.0019 | 0.018822 | 159.5568 | 9.43E-37 |
|  | rs7679276 | -0.033 | 0.0048 | -0.01025 | 47.26563 | 5.93E-12 |
|  | rs395980 | -0.0184 | 0.0021 | -0.01306 | 76.77098 | 1.02E-17 |
|  | rs2578565 | -0.0141 | 0.002 | -0.01051 | 49.7025 | 1.37E-12 |
|  | rs12655296 | -0.011 | 0.002 | -0.0082 | 30.25 | 1.62E-08 |
|  | rs1177765 | -0.0232 | 0.0019 | -0.01819 | 149.097 | 1.32E-34 |
|  | rs11959466 | 0.038 | 0.0042 | 0.013483 | 81.85941 | 2.24E-19 |
|  | rs62370472 | -0.0253 | 0.0023 | -0.01639 | 121 | 1.45E-27 |
|  | rs10471339 | -0.011 | 0.0019 | -0.00863 | 33.51801 | 1.45E-08 |
|  | rs36048468 | 0.0254 | 0.0023 | 0.016456 | 121.9584 | 9.34E-28 |
|  | rs7735891 | 0.0259 | 0.0019 | 0.020311 | 185.8199 | 1.14E-42 |
|  | rs55758152 | 0.0145 | 0.002 | 0.010804 | 52.5625 | 1.05E-12 |
|  | rs57059662 | 0.0118 | 0.002 | 0.008793 | 34.81 | 5.63E-09 |
|  | rs34313173 | -0.0284 | 0.0019 | -0.02227 | 223.4238 | 3.17E-50 |
|  | rs10461725 | 0.0134 | 0.002 | 0.009985 | 44.89 | 1.98E-11 |
|  | rs4865956 | -0.0258 | 0.0021 | -0.01831 | 150.9388 | 3.85E-36 |
|  | rs12517711 | -0.0147 | 0.0019 | -0.01153 | 59.85873 | 2.79E-14 |
|  | rs12188208 | -0.0195 | 0.0022 | -0.01321 | 78.56405 | 1.4E-18 |
|  | rs115912456 | 0.0577 | 0.0047 | 0.018293 | 150.7148 | 3.69E-34 |
|  | rs861674 | 0.0128 | 0.0019 | 0.010039 | 45.38504 | 1.36E-11 |
|  | rs10068640 | 0.0112 | 0.002 | 0.008345 | 31.36 | 1.28E-08 |
|  | rs2545339 | 0.0115 | 0.002 | 0.008569 | 33.0625 | 3.48E-09 |
|  | rs144622623 | 0.0145 | 0.0022 | 0.009822 | 43.44008 | 4.67E-11 |
|  | rs31196 | -0.0107 | 0.0019 | -0.00839 | 31.71468 | 2.07E-08 |
|  | rs111365325 | -0.0271 | 0.0022 | -0.01835 | 151.7376 | 1.12E-33 |
|  | rs6874142 | 0.0288 | 0.0031 | 0.013844 | 86.31009 | 5.15E-20 |
|  | rs10075249 | 0.0143 | 0.0019 | 0.011216 | 56.64543 | 4.56E-14 |
|  | rs10036789 | 0.0163 | 0.0019 | 0.012784 | 73.59834 | 1.16E-17 |
|  | rs33986149 | -0.0154 | 0.0019 | -0.01208 | 65.69529 | 2.23E-15 |
|  | rs261223 | 0.0175 | 0.0019 | 0.013725 | 84.8338 | 2.3E-19 |
|  | rs12519407 | 0.0181 | 0.0022 | 0.01226 | 67.68802 | 3.38E-17 |
|  | rs249677 | -0.0109 | 0.002 | -0.00812 | 29.7025 | 2.4E-08 |
|  | rs13170063 | -0.0152 | 0.0019 | -0.01192 | 64 | 4.11E-15 |
|  | rs447352 | -0.0181 | 0.0029 | -0.0093 | 38.95482 | 6.62E-10 |
|  | rs7731023 | 0.0166 | 0.0019 | 0.01302 | 76.33241 | 3.48E-18 |
|  | rs7448554 | -0.0132 | 0.002 | -0.00984 | 43.56 | 1.77E-11 |
|  | rs3822742 | 0.0162 | 0.002 | 0.012071 | 65.61 | 1.03E-16 |
|  | rs4282339 | -0.0311 | 0.0023 | -0.02015 | 182.8374 | 6.16E-41 |
|  | rs244711 | 0.0279 | 0.0022 | 0.018897 | 160.8285 | 1.54E-37 |
|  | rs40270 | 0.0151 | 0.0022 | 0.010228 | 47.1095 | 1.9E-11 |
|  | rs34287 | 0.0187 | 0.002 | 0.013933 | 87.4225 | 1.17E-20 |
|  | rs331917 | -0.0127 | 0.0019 | -0.00996 | 44.67867 | 3.54E-11 |
|  | rs6860245 | 0.0589 | 0.0022 | 0.039868 | 716.7789 | 9.7E-160 |
|  | rs4976262 | -0.0245 | 0.002 | -0.01825 | 150.0625 | 4.37E-33 |
|  | rs258794 | 0.0147 | 0.0021 | 0.010432 | 49 | 6.23E-12 |
|  | rs7701233 | -0.0179 | 0.0019 | -0.01404 | 88.75623 | 5.12E-21 |
|  | rs3792819 | 0.021 | 0.0034 | 0.009204 | 38.14879 | 4.42E-10 |
|  | rs11243202 | 0.0302 | 0.0019 | 0.023681 | 252.6427 | 2.83E-57 |
|  | rs13209685 | 0.0277 | 0.0026 | 0.015876 | 113.5044 | 7.49E-27 |
|  | rs2142644 | -0.0181 | 0.002 | -0.01349 | 81.9025 | 3.37E-19 |
|  | rs78000963 | 0.0169 | 0.0031 | 0.008124 | 29.72008 | 4.39E-08 |
|  | rs370927791 | -0.0208 | 0.002 | -0.0155 | 108.16 | 4.99E-25 |
|  | rs2268718 | 0.0141 | 0.0021 | 0.010006 | 45.08163 | 3.24E-11 |
|  | rs6931421 | -0.0279 | 0.002 | -0.02079 | 194.6025 | 2.31E-43 |
|  | rs9375188 | 0.0136 | 0.0019 | 0.010667 | 51.23546 | 6.8E-13 |
|  | rs9391254 | 0.0166 | 0.002 | 0.012369 | 68.89 | 2.1E-16 |
|  | rs113898003 | -0.036 | 0.0021 | -0.02554 | 293.8776 | 1.19E-63 |
|  | rs1933081 | 0.0267 | 0.0034 | 0.011703 | 61.66869 | 5.33E-15 |
|  | rs141641494 | -0.0115 | 0.002 | -0.00857 | 33.0625 | 8.39E-09 |
|  | rs9385002 | -0.0147 | 0.0022 | -0.00996 | 44.64669 | 3.26E-11 |
|  | rs7768382 | -0.0201 | 0.0019 | -0.01576 | 111.9141 | 1.57E-26 |
|  | rs372987459 | 0.0147 | 0.002 | 0.010953 | 54.0225 | 6.85E-14 |
|  | rs2788213 | 0.0123 | 0.0021 | 0.008729 | 34.30612 | 3.8E-09 |
|  | rs2569888 | 0.0133 | 0.0022 | 0.009009 | 36.54752 | 2.29E-09 |
|  | rs876122 | 0.0162 | 0.0029 | 0.008325 | 31.20571 | 2.19E-08 |
|  | rs41271299 | 0.0616 | 0.0043 | 0.021345 | 205.2223 | 3.19E-47 |
|  | rs188617336 | 0.0138 | 0.0021 | 0.009793 | 43.18367 | 6.66E-11 |
|  | rs4380799 | -0.0255 | 0.0021 | -0.01809 | 147.449 | 6.45E-33 |
|  | rs1319012 | -0.052 | 0.0037 | -0.02094 | 197.5164 | 3.3E-45 |
|  | rs1324538 | 0.0237 | 0.0019 | 0.018586 | 155.5928 | 1.73E-34 |
|  | rs655113 | 0.0188 | 0.0021 | 0.013341 | 80.14512 | 7.42E-20 |
|  | rs9344126 | -0.0185 | 0.0019 | -0.01451 | 94.80609 | 2.21E-22 |
|  | rs293517 | -0.013 | 0.0021 | -0.00923 | 38.322 | 2.8E-10 |
|  | rs7768973 | -0.024 | 0.0019 | -0.01882 | 159.5568 | 5.44E-36 |
|  | rs78051210 | 0.0263 | 0.0036 | 0.010887 | 53.37114 | 1.63E-13 |
|  | rs10807137 | -0.0455 | 0.0025 | -0.02711 | 331.24 | 5.68E-75 |
|  | rs9343327 | 0.014 | 0.0019 | 0.010981 | 54.29363 | 1.09E-13 |
|  | rs35166681 | 0.0214 | 0.0026 | 0.012265 | 67.74556 | 1.55E-16 |
|  | rs2754255 | -0.0153 | 0.0023 | -0.00991 | 44.25142 | 1.05E-11 |
|  | rs9388490 | 0.0462 | 0.0019 | 0.036214 | 591.2576 | 1.3E-130 |
|  | rs6902109 | -0.0167 | 0.0019 | -0.0131 | 77.25485 | 1.03E-18 |
|  | rs599004 | -0.0157 | 0.0021 | -0.01114 | 55.89342 | 6.53E-14 |
|  | rs2748501 | -0.0195 | 0.0019 | -0.01529 | 105.3324 | 1.31E-24 |
|  | rs718603 | 0.0131 | 0.0021 | 0.009296 | 38.91383 | 6.68E-10 |
|  | rs9266244 | -0.0427 | 0.0021 | -0.03029 | 413.4444 | 1.21E-94 |
|  | rs72894003 | -0.0423 | 0.0038 | -0.01659 | 123.912 | 1.9E-28 |
|  | rs2764264 | 0.0203 | 0.0021 | 0.014405 | 93.44444 | 5.07E-23 |
|  | rs6570509 | -0.0244 | 0.0021 | -0.01731 | 135.0023 | 1.27E-31 |
|  | rs543650 | 0.025 | 0.002 | 0.018626 | 156.25 | 1.49E-37 |
|  | rs3828729 | -0.016 | 0.002 | -0.01192 | 64 | 4.67E-15 |
|  | rs2105333 | -0.019 | 0.002 | -0.01416 | 90.25 | 1.7E-21 |
|  | rs2763263 | -0.017 | 0.0022 | -0.01152 | 59.71074 | 1.37E-14 |
|  | rs798548 | -0.0359 | 0.0021 | -0.02547 | 292.2472 | 2.86E-68 |
|  | rs12533452 | 0.0237 | 0.0026 | 0.013584 | 83.09024 | 1.31E-19 |
|  | rs34776209 | -0.0317 | 0.0022 | -0.02147 | 207.6219 | 1.78E-47 |
|  | rs12536902 | 0.0479 | 0.0081 | 0.008813 | 34.97043 | 3.66E-09 |
|  | rs1880318 | 0.0147 | 0.0024 | 0.009128 | 37.51563 | 6.86E-10 |
|  | rs73696333 | 0.0191 | 0.0024 | 0.01186 | 63.33507 | 3.2E-15 |
|  | rs35732917 | 0.0204 | 0.0021 | 0.014476 | 94.36735 | 2.08E-22 |
|  | rs139163241 | -0.0164 | 0.0027 | -0.00905 | 36.89438 | 1.79E-09 |
|  | rs62466110 | -0.0371 | 0.0041 | -0.01348 | 81.88043 | 5.74E-20 |
|  | rs143355941 | 0.0189 | 0.0019 | 0.014823 | 98.95014 | 4.65E-23 |
|  | rs987666 | 0.0185 | 0.0029 | 0.009507 | 40.6956 | 2.33E-10 |
|  | rs62621812 | 0.0743 | 0.0069 | 0.016046 | 115.9523 | 3.16E-27 |
|  | rs757834 | 0.0256 | 0.0024 | 0.015895 | 113.7778 | 1.25E-25 |
|  | rs28529426 | -0.0168 | 0.0026 | -0.00963 | 41.75148 | 5.82E-11 |
|  | rs10242866 | 0.0157 | 0.0019 | 0.012314 | 68.27978 | 3.66E-16 |
|  | rs2529090 | 0.0136 | 0.0025 | 0.008107 | 29.5936 | 3.39E-08 |
|  | rs2237485 | 0.0191 | 0.0023 | 0.012375 | 68.96219 | 3.73E-17 |
|  | rs10627558 | -0.0181 | 0.0022 | -0.01226 | 67.68802 | 6.91E-16 |
|  | rs12672217 | 0.0139 | 0.002 | 0.010357 | 48.3025 | 1.33E-12 |
|  | rs177591 | -0.0191 | 0.0027 | -0.01054 | 50.04252 | 1.51E-12 |
|  | rs60408354 | 0.0259 | 0.0036 | 0.010721 | 51.76003 | 1.15E-12 |
|  | rs42039 | 0.0481 | 0.0022 | 0.032566 | 478.0186 | 3.5E-106 |
|  | rs56363908 | -0.0382 | 0.0047 | -0.01211 | 66.05885 | 3.88E-16 |
|  | rs2140619 | 0.0113 | 0.0019 | 0.008863 | 35.37119 | 5.18E-09 |
|  | rs3778858 | 0.0108 | 0.002 | 0.008047 | 29.16 | 4.26E-08 |
|  | rs822530 | 0.0255 | 0.0024 | 0.015833 | 112.8906 | 2.36E-27 |
|  | rs12702693 | 0.0173 | 0.0019 | 0.013568 | 82.90582 | 6.56E-20 |
|  | rs680882 | 0.0133 | 0.0022 | 0.009009 | 36.54752 | 1.98E-09 |
|  | rs723149 | -0.0276 | 0.0019 | -0.02164 | 211.0139 | 1.43E-47 |
|  | rs1202186 | -0.012 | 0.002 | -0.00894 | 36 | 1.65E-09 |
|  | rs9640283 | -0.0119 | 0.0019 | -0.00933 | 39.22715 | 4.53E-10 |
|  | rs6977416 | 0.0457 | 0.002 | 0.034034 | 522.1225 | 1.4E-113 |
|  | rs10225945 | -0.0146 | 0.0026 | -0.00837 | 31.53254 | 3.28E-08 |
|  | rs12700901 | -0.0184 | 0.0019 | -0.01443 | 93.78393 | 1.67E-21 |
|  | rs6593210 | 0.0146 | 0.0024 | 0.009066 | 37.00694 | 4.88E-10 |
|  | rs11562101 | 0.0115 | 0.002 | 0.008569 | 33.0625 | 9.39E-09 |
|  | rs2188805 | 0.0114 | 0.002 | 0.008494 | 32.49 | 2E-08 |
|  | rs76364830 | -0.0471 | 0.0039 | -0.018 | 145.8521 | 2.7E-33 |
|  | rs7826059 | 0.0114 | 0.002 | 0.008494 | 32.49 | 7.61E-09 |
|  | rs1063582 | -0.0185 | 0.0022 | -0.01253 | 70.71281 | 1.12E-16 |
|  | rs117818446 | 0.0423 | 0.0068 | 0.00927 | 38.69572 | 5.29E-10 |
|  | rs7014590 | -0.0228 | 0.0022 | -0.01544 | 107.405 | 4.48E-26 |
|  | rs112537273 | -0.0212 | 0.0022 | -0.01436 | 92.8595 | 3.34E-21 |
|  | rs4602848 | 0.016 | 0.002 | 0.011922 | 64 | 3.14E-15 |
|  | rs35086476 | 0.0109 | 0.0019 | 0.008549 | 32.91136 | 8.43E-09 |
|  | rs2142331 | -0.0165 | 0.0019 | -0.01294 | 75.41551 | 1.38E-17 |
|  | rs6470771 | -0.0268 | 0.0025 | -0.01597 | 114.9184 | 1.54E-26 |
|  | rs12334478 | -0.0161 | 0.0019 | -0.01263 | 71.80332 | 2.27E-17 |
|  | rs10112506 | -0.012 | 0.0019 | -0.00941 | 39.8892 | 5.76E-10 |
|  | rs7816345 | 0.0255 | 0.0025 | 0.015199 | 104.04 | 6.22E-24 |
|  | rs2923411 | 0.0127 | 0.0019 | 0.009961 | 44.67867 | 4.77E-11 |
|  | rs72656010 | -0.0668 | 0.0028 | -0.03553 | 569.1633 | 7.3E-126 |
|  | rs7828086 | 0.0135 | 0.0022 | 0.009145 | 37.65496 | 1.11E-09 |
|  | rs62501195 | -0.0198 | 0.0025 | -0.0118 | 62.7264 | 8.11E-15 |
|  | rs62515437 | 0.0369 | 0.0023 | 0.023903 | 257.3932 | 8.79E-60 |
|  | rs2925155 | -0.015 | 0.0022 | -0.01016 | 46.4876 | 5.47E-12 |
|  | rs11778491 | -0.0247 | 0.0022 | -0.01673 | 126.0517 | 8.39E-30 |
|  | rs1340022 | 0.0118 | 0.0019 | 0.009255 | 38.57064 | 4.47E-10 |
|  | rs72721979 | -0.0229 | 0.0027 | -0.01264 | 71.93553 | 2.2E-17 |
|  | rs7007389 | -0.0134 | 0.002 | -0.00998 | 44.89 | 2.94E-11 |
|  | rs115105539 | 0.0231 | 0.0025 | 0.013769 | 85.3776 | 7.83E-20 |
|  | rs4077103 | -0.0143 | 0.0026 | -0.0082 | 30.25 | 4.18E-08 |
|  | rs61729527 | -0.0346 | 0.0043 | -0.01199 | 64.74635 | 4.86E-16 |
|  | rs4735761 | 0.0331 | 0.0021 | 0.023484 | 248.4376 | 3.66E-56 |
|  | rs10283100 | 0.0575 | 0.0041 | 0.020896 | 196.6835 | 4.11E-44 |
|  | rs4870941 | -0.0297 | 0.0023 | -0.01924 | 166.7467 | 1.09E-39 |
|  | rs12541381 | -0.0319 | 0.0022 | -0.0216 | 210.25 | 2.81E-49 |
|  | rs10107388 | -0.0159 | 0.002 | -0.01185 | 63.2025 | 6.95E-16 |
|  | rs10815274 | 0.0124 | 0.0019 | 0.009726 | 42.5928 | 6.46E-11 |
|  | rs7858712 | 0.0347 | 0.0034 | 0.015208 | 104.16 | 1.04E-24 |
|  | rs34522021 | 0.0126 | 0.0019 | 0.009883 | 43.97784 | 3.38E-11 |
|  | rs75508358 | 0.0266 | 0.0045 | 0.008809 | 34.94123 | 4.29E-09 |
|  | rs12347137 | -0.046 | 0.0024 | -0.02855 | 367.3611 | 9.8E-85 |
|  | rs10123619 | -0.0171 | 0.0026 | -0.0098 | 43.25592 | 4.05E-11 |
|  | rs10975935 | -0.0121 | 0.0022 | -0.0082 | 30.25 | 4.15E-08 |
|  | rs1056747 | -0.0155 | 0.0019 | -0.01216 | 66.55125 | 8.05E-16 |
|  | rs143554698 | -0.0257 | 0.0027 | -0.01418 | 90.60219 | 3.39E-21 |
|  | rs10982888 | -0.0328 | 0.003 | -0.01629 | 119.5378 | 3.97E-28 |
|  | rs73384223 | -0.0205 | 0.0024 | -0.01273 | 72.96007 | 1.27E-17 |
|  | rs10962212 | 0.0143 | 0.0019 | 0.011216 | 56.64543 | 7.47E-14 |
|  | rs7863102 | -0.011 | 0.0019 | -0.00863 | 33.51801 | 9.97E-09 |
|  | rs3901421 | 0.0215 | 0.0019 | 0.016862 | 128.0471 | 7.55E-30 |
|  | rs2236406 | 0.0394 | 0.002 | 0.029347 | 388.09 | 1.26E-87 |
|  | rs373966865 | 0.0389 | 0.0025 | 0.023183 | 242.1136 | 1.84E-53 |
|  | rs3205136 | -0.0184 | 0.0033 | -0.00831 | 31.08907 | 1.85E-08 |
|  | rs10858246 | -0.0188 | 0.002 | -0.01401 | 88.36 | 2.35E-20 |
|  | rs1330826 | 0.0162 | 0.0023 | 0.010496 | 49.61059 | 1.04E-12 |
|  | rs7020491 | -0.0178 | 0.0019 | -0.01396 | 87.76731 | 1.22E-20 |
|  | rs74458759 | 0.0171 | 0.0022 | 0.011583 | 60.41529 | 3.12E-15 |
|  | rs12340775 | -0.0287 | 0.0043 | -0.00995 | 44.54786 | 1.73E-11 |
|  | rs12351226 | 0.0218 | 0.0025 | 0.012994 | 76.0384 | 9.13E-18 |
|  | rs1341215 | 0.0229 | 0.0027 | 0.012639 | 71.93553 | 6.32E-17 |
|  | rs12344515 | -0.0163 | 0.0022 | -0.01104 | 54.89463 | 2.28E-13 |
|  | rs80280630 | -0.0168 | 0.003 | -0.00835 | 31.36 | 2.28E-08 |
|  | rs112367251 | 0.0179 | 0.0021 | 0.012702 | 72.65533 | 1.29E-17 |
|  | rs10793931 | -0.0132 | 0.002 | -0.00984 | 43.56 | 3.23E-11 |
|  | rs7082659 | 0.0156 | 0.0028 | 0.008303 | 31.04082 | 2.27E-08 |
|  | rs10829226 | -0.0112 | 0.002 | -0.00835 | 31.36 | 1.33E-08 |
|  | rs10776560 | -0.0157 | 0.0019 | -0.01231 | 68.27978 | 7.88E-17 |
|  | rs68049170 | -0.0259 | 0.0021 | -0.01838 | 152.1111 | 2.69E-34 |
|  | rs2274351 | 0.017 | 0.0019 | 0.013333 | 80.0554 | 3.07E-19 |
|  | rs72841270 | 0.0294 | 0.0028 | 0.015646 | 110.25 | 2.25E-26 |
|  | rs11421589 | 0.0119 | 0.0019 | 0.009334 | 39.22715 | 5.99E-10 |
|  | rs11424084 | 0.0228 | 0.0027 | 0.012584 | 71.30864 | 7.24E-17 |
|  | rs1556659 | 0.0163 | 0.002 | 0.012145 | 66.4225 | 7.19E-17 |
|  | rs35288270 | -0.0328 | 0.0028 | -0.01746 | 137.2245 | 3.43E-32 |
|  | rs4748008 | -0.0125 | 0.0019 | -0.0098 | 43.28255 | 8.95E-11 |
|  | rs332116 | -0.0206 | 0.0021 | -0.01462 | 96.22676 | 2.89E-22 |
|  | rs10822117 | -0.0176 | 0.0022 | -0.01192 | 64 | 4.24E-15 |
|  | rs67527161 | -0.0182 | 0.0023 | -0.01179 | 62.61626 | 5.79E-15 |
|  | rs10128333 | -0.0146 | 0.0025 | -0.0087 | 34.1056 | 9.51E-09 |
|  | rs7095472 | 0.0267 | 0.0019 | 0.020938 | 197.4765 | 7.66E-45 |
|  | rs117335233 | -0.0236 | 0.0042 | -0.00837 | 31.5737 | 2.56E-08 |
|  | rs12773500 | 0.0171 | 0.0028 | 0.009101 | 37.29719 | 5.05E-10 |
|  | rs11187838 | 0.0394 | 0.0019 | 0.03089 | 430.0166 | 1.16E-94 |
|  | rs2181834 | 0.0254 | 0.0019 | 0.019919 | 178.7147 | 7.69E-41 |
|  | rs496783 | -0.0124 | 0.0019 | -0.00973 | 42.5928 | 8.13E-11 |
|  | rs11198591 | 0.0148 | 0.002 | 0.011028 | 54.76 | 4.91E-14 |
|  | rs2362487 | 0.0154 | 0.0022 | 0.010432 | 49 | 3.57E-12 |
|  | rs947099 | 0.0117 | 0.002 | 0.008718 | 34.2225 | 2.71E-09 |
|  | rs71463518 | -0.0132 | 0.0021 | -0.00937 | 39.5102 | 2.92E-10 |
|  | rs10824307 | -0.0194 | 0.002 | -0.01445 | 94.09 | 1.79E-22 |
|  | rs664317 | -0.0177 | 0.0026 | -0.01015 | 46.34467 | 4.5E-12 |
|  | rs2648725 | 0.0165 | 0.0023 | 0.010691 | 51.46503 | 8.2E-13 |
|  | rs7893378 | 0.0175 | 0.0031 | 0.008413 | 31.86785 | 2.36E-08 |
|  | rs291979 | 0.0242 | 0.0023 | 0.015679 | 110.707 | 6.76E-27 |
|  | rs11009928 | -0.0148 | 0.0022 | -0.01003 | 45.2562 | 1.04E-11 |
|  | rs5786398 | -0.0177 | 0.0021 | -0.01256 | 71.04082 | 1.54E-17 |
|  | rs4752689 | 0.0205 | 0.0019 | 0.016078 | 116.4127 | 1.36E-26 |
|  | rs11014285 | 0.0342 | 0.0026 | 0.0196 | 173.0237 | 2.89E-40 |
|  | rs2490302 | 0.0221 | 0.0034 | 0.009687 | 42.25 | 6.24E-11 |
|  | rs11191208 | 0.0147 | 0.0024 | 0.009128 | 37.51563 | 3.69E-10 |
|  | rs10749157 | 0.0113 | 0.002 | 0.00842 | 31.9225 | 1.24E-08 |
|  | rs2283200 | -0.0281 | 0.0042 | -0.00997 | 44.76247 | 1.48E-11 |
|  | rs73413540 | -0.0124 | 0.0023 | -0.00803 | 29.06616 | 4.42E-08 |
|  | rs985136 | 0.0138 | 0.002 | 0.010283 | 47.61 | 2.96E-12 |
|  | rs4752829 | 0.0262 | 0.0021 | 0.01859 | 155.6553 | 5.9E-36 |
|  | rs10796828 | 0.0154 | 0.002 | 0.011475 | 59.29 | 5.77E-15 |
|  | rs7902 | 0.0149 | 0.0019 | 0.011686 | 61.49861 | 5.22E-15 |
|  | rs11221657 | 0.0179 | 0.0028 | 0.009527 | 40.86862 | 1.21E-10 |
|  | rs112873218 | 0.0216 | 0.0031 | 0.010384 | 48.54943 | 4.02E-12 |
|  | rs10832963 | -0.0203 | 0.0022 | -0.01375 | 85.14256 | 9.23E-21 |
|  | rs704660 | 0.0153 | 0.0019 | 0.012 | 64.84488 | 2.28E-15 |
|  | rs7107356 | 0.0133 | 0.0019 | 0.010432 | 49 | 1.86E-12 |
|  | rs11233117 | -0.0176 | 0.0019 | -0.0138 | 85.80609 | 1.74E-20 |
|  | rs73006226 | -0.0182 | 0.0029 | -0.00935 | 39.38644 | 2.23E-10 |
|  | rs545104 | 0.0127 | 0.002 | 0.009463 | 40.3225 | 8.08E-11 |
|  | rs56207600 | 0.0192 | 0.003 | 0.009538 | 40.96 | 2.5E-10 |
|  | rs61878760 | 0.019 | 0.0034 | 0.008328 | 31.22837 | 3.74E-08 |
|  | rs7952436 | -0.0453 | 0.0034 | -0.01985 | 177.5164 | 1.62E-39 |
|  | rs4244809 | -0.0262 | 0.0023 | -0.01697 | 129.7618 | 5.55E-29 |
|  | rs1584011 | 0.0159 | 0.002 | 0.011847 | 63.2025 | 9.78E-16 |
|  | rs7941305 | -0.0129 | 0.0021 | -0.00915 | 37.73469 | 6.01E-10 |
|  | rs11605297 | 0.0146 | 0.0022 | 0.00989 | 44.04132 | 8.03E-11 |
|  | rs7129320 | -0.0389 | 0.0025 | -0.02318 | 242.1136 | 7.29E-53 |
|  | rs34345560 | 0.0219 | 0.0024 | 0.013598 | 83.26563 | 7.1E-20 |
|  | rs604723 | -0.0166 | 0.0021 | -0.01178 | 62.48526 | 8.16E-15 |
|  | rs11042717 | -0.029 | 0.0019 | -0.02274 | 232.964 | 4.04E-53 |
|  | rs10657263 | -0.013 | 0.0019 | -0.0102 | 46.8144 | 7.98E-12 |
|  | rs4938359 | -0.0156 | 0.0024 | -0.00969 | 42.25 | 3.24E-11 |
|  | rs11217863 | -0.0268 | 0.003 | -0.01331 | 79.80444 | 1.06E-19 |
|  | rs772222 | 0.0121 | 0.0021 | 0.008587 | 33.19955 | 1.55E-08 |
|  | rs1168768 | 0.0332 | 0.006 | 0.008246 | 30.61778 | 3.61E-08 |
|  | rs2089111 | -0.0172 | 0.0022 | -0.01165 | 61.12397 | 1.73E-15 |
|  | rs4622329 | 0.0149 | 0.002 | 0.011102 | 55.5025 | 8.62E-14 |
|  | rs3764002 | 0.028 | 0.0021 | 0.019867 | 177.7778 | 4.47E-39 |
|  | rs12423821 | 0.0161 | 0.0027 | 0.008886 | 35.55693 | 1.17E-09 |
|  | rs7137546 | 0.0142 | 0.0019 | 0.011137 | 55.85596 | 9.71E-14 |
|  | rs67551338 | 0.0576 | 0.004 | 0.021456 | 207.36 | 1.04E-47 |
|  | rs10845408 | 0.0255 | 0.002 | 0.018998 | 162.5625 | 3.25E-38 |
|  | rs17478946 | -0.0192 | 0.0021 | -0.01362 | 83.59184 | 9.98E-21 |
|  | rs12230946 | 0.0271 | 0.0033 | 0.012238 | 67.43893 | 1.45E-16 |
|  | rs9669278 | -0.0496 | 0.0019 | -0.03888 | 681.4848 | 5.2E-151 |
|  | rs2229840 | 0.0341 | 0.0026 | 0.019542 | 172.0133 | 3.02E-40 |
|  | rs7485647 | -0.0261 | 0.0026 | -0.01496 | 100.7707 | 1.04E-23 |
|  | rs35756741 | -0.0378 | 0.0033 | -0.01707 | 131.2066 | 5.8E-31 |
|  | rs6582398 | 0.014 | 0.002 | 0.010432 | 49 | 1.14E-12 |
|  | rs10748128 | 0.0255 | 0.002 | 0.018998 | 162.5625 | 6.77E-38 |
|  | rs11178643 | 0.0109 | 0.002 | 0.008122 | 29.7025 | 4.44E-08 |
|  | rs310796 | 0.0142 | 0.002 | 0.010581 | 50.41 | 2.53E-12 |
|  | rs9634212 | 0.0471 | 0.0023 | 0.030505 | 419.3592 | 8.59E-95 |
|  | rs7971536 | -0.0194 | 0.0019 | -0.01522 | 104.2548 | 1.06E-24 |
|  | rs2454390 | -0.0176 | 0.0026 | -0.01009 | 45.82249 | 1.74E-11 |
|  | rs11612462 | 0.015 | 0.0025 | 0.008942 | 36 | 2.55E-09 |
|  | rs34338597 | -0.0112 | 0.0019 | -0.00878 | 34.74792 | 7.88E-09 |
|  | rs3184504 | 0.0183 | 0.0019 | 0.014353 | 92.76731 | 2.71E-22 |
|  | rs610694 | 0.0136 | 0.0019 | 0.010667 | 51.23546 | 4.29E-13 |
|  | rs76895963 | 0.1639 | 0.0073 | 0.033442 | 504.0948 | 8.2E-112 |
|  | rs11175919 | 0.0349 | 0.0059 | 0.008815 | 34.99023 | 3.16E-09 |
|  | rs11068230 | 0.0238 | 0.0028 | 0.012667 | 72.25 | 9.67E-18 |
|  | rs2101017 | -0.0223 | 0.0028 | -0.01187 | 63.42985 | 1.44E-15 |
|  | rs11060942 | -0.0354 | 0.0052 | -0.01015 | 46.34467 | 7.49E-12 |
|  | rs28592876 | 0.03 | 0.0023 | 0.019435 | 170.1323 | 9.09E-38 |
|  | rs3782811 | -0.0165 | 0.0022 | -0.01118 | 56.25 | 3.96E-14 |
|  | rs61919240 | 0.0137 | 0.002 | 0.010208 | 46.9225 | 9.77E-12 |
|  | rs1444628 | 0.024 | 0.002 | 0.017881 | 144 | 6.85E-32 |
|  | rs11049704 | -0.0183 | 0.0021 | -0.01299 | 75.93878 | 1.1E-18 |
|  | rs12831751 | 0.0172 | 0.0021 | 0.012205 | 67.0839 | 1.57E-16 |
|  | rs12099669 | 0.0331 | 0.002 | 0.024657 | 273.9025 | 1.39E-58 |
|  | rs2071450 | -0.0174 | 0.002 | -0.01296 | 75.69 | 8.85E-19 |
|  | rs3782232 | -0.0339 | 0.0037 | -0.01365 | 83.94522 | 2.41E-20 |
|  | rs7301341 | -0.0255 | 0.002 | -0.019 | 162.5625 | 9.27E-37 |
|  | rs7321635 | -0.0132 | 0.002 | -0.00984 | 43.56 | 2.58E-11 |
|  | rs3116602 | -0.0612 | 0.0023 | -0.03962 | 708.0227 | 9.5E-155 |
|  | rs3818416 | 0.0279 | 0.0022 | 0.018897 | 160.8285 | 2.01E-35 |
|  | rs61944841 | 0.0253 | 0.002 | 0.018849 | 160.0225 | 3.54E-37 |
|  | rs77013652 | 0.049 | 0.0081 | 0.009015 | 36.59503 | 1.5E-09 |
|  | rs7328187 | 0.0116 | 0.0019 | 0.009098 | 37.27424 | 1.19E-09 |
|  | rs7320878 | -0.015 | 0.0019 | -0.01176 | 62.32687 | 1.34E-14 |
|  | rs144109601 | -0.0278 | 0.0048 | -0.00863 | 33.5434 | 5.15E-09 |
|  | rs9590328 | 0.0153 | 0.0027 | 0.008445 | 32.11111 | 2.02E-08 |
|  | rs78525785 | -0.0169 | 0.002 | -0.01259 | 71.4025 | 9.81E-18 |
|  | rs9594714 | 0.0144 | 0.0021 | 0.010219 | 47.02041 | 2.65E-12 |
|  | rs9568031 | -0.0115 | 0.0021 | -0.00816 | 29.98866 | 3.34E-08 |
|  | rs2812208 | 0.1156 | 0.0066 | 0.026094 | 306.7805 | 5.51E-68 |
|  | rs8000973 | 0.0134 | 0.0019 | 0.01051 | 49.73961 | 2.05E-12 |
|  | rs9525326 | -0.0184 | 0.0024 | -0.01142 | 58.77778 | 3.62E-14 |
|  | rs532499 | -0.0127 | 0.0022 | -0.0086 | 33.32438 | 4.9E-09 |
|  | rs9517483 | -0.0181 | 0.0021 | -0.01284 | 74.28798 | 2.26E-18 |
|  | rs2296316 | -0.0192 | 0.0019 | -0.01506 | 102.1163 | 1.59E-23 |
|  | rs113827862 | -0.0235 | 0.004 | -0.00876 | 34.51563 | 4.67E-09 |
|  | rs4900578 | -0.0177 | 0.002 | -0.01319 | 78.3225 | 1.76E-19 |
|  | rs56112295 | 0.0154 | 0.0024 | 0.009562 | 41.17361 | 1.12E-10 |
|  | rs8019890 | 0.025 | 0.0019 | 0.019606 | 173.1302 | 1.96E-38 |
|  | rs8017006 | 0.0122 | 0.002 | 0.009091 | 37.21 | 2.22E-09 |
|  | rs10483727 | -0.0368 | 0.0019 | -0.02885 | 375.1357 | 6.73E-80 |
|  | rs8020095 | -0.0145 | 0.0027 | -0.008 | 28.84088 | 4.53E-08 |
|  | rs35230100 | -0.0262 | 0.002 | -0.01952 | 171.61 | 4.42E-40 |
|  | rs117068593 | 0.0403 | 0.0024 | 0.025017 | 281.9601 | 8.83E-62 |
|  | rs1190540 | 0.0125 | 0.0021 | 0.008871 | 35.43084 | 1.62E-09 |
|  | rs17197114 | 0.0177 | 0.0025 | 0.010551 | 50.1264 | 1.54E-12 |
|  | rs45528934 | 0.0262 | 0.0026 | 0.015016 | 101.5444 | 1.97E-24 |
|  | rs28529055 | -0.0147 | 0.0019 | -0.01153 | 59.85873 | 1.88E-14 |
|  | rs36226649 | 0.0485 | 0.0038 | 0.019018 | 162.8982 | 3.05E-37 |
|  | rs28678024 | -0.0119 | 0.0021 | -0.00844 | 32.11111 | 1.9E-08 |
|  | rs8904 | -0.0157 | 0.002 | -0.0117 | 61.6225 | 1.52E-15 |
|  | rs10637890 | -0.0123 | 0.002 | -0.00917 | 37.8225 | 3.96E-10 |
|  | rs2070598 | 0.0204 | 0.0019 | 0.015999 | 115.2798 | 6.36E-27 |
|  | rs909220 | -0.015 | 0.0019 | -0.01176 | 62.32687 | 3.24E-15 |
|  | rs79066296 | -0.0169 | 0.0022 | -0.01145 | 59.01033 | 6.11E-14 |
|  | rs12882130 | -0.0202 | 0.002 | -0.01505 | 102.01 | 1.88E-24 |
|  | rs8018486 | -0.0138 | 0.0024 | -0.00857 | 33.0625 | 1.18E-08 |
|  | rs7144307 | -0.0122 | 0.002 | -0.00909 | 37.21 | 6.1E-10 |
|  | rs13316 | 0.0115 | 0.0019 | 0.00902 | 36.63435 | 3.66E-09 |
|  | rs147233090 | -0.0446 | 0.0061 | -0.0109 | 53.45767 | 3.95E-13 |
|  | rs5812543 | 0.0135 | 0.0021 | 0.00958 | 41.32653 | 2.65E-10 |
|  | rs4383083 | 0.0111 | 0.002 | 0.008271 | 30.8025 | 2.91E-08 |
|  | rs8042578 | 0.0287 | 0.0022 | 0.019438 | 170.1839 | 2.29E-38 |
|  | rs990315 | -0.0115 | 0.002 | -0.00857 | 33.0625 | 5.07E-09 |
|  | rs74379684 | -0.0272 | 0.0036 | -0.01126 | 57.08642 | 4.39E-14 |
|  | rs2871865 | -0.0493 | 0.003 | -0.02448 | 270.0544 | 3.4E-62 |
|  | rs17205463 | -0.0263 | 0.0019 | -0.02062 | 191.6039 | 4.21E-43 |
|  | rs36016415 | -0.0275 | 0.0019 | -0.02157 | 209.4875 | 3.22E-47 |
|  | rs4965298 | -0.0119 | 0.0021 | -0.00844 | 32.11111 | 1.81E-08 |
|  | rs11070842 | -0.0146 | 0.0026 | -0.00837 | 31.53254 | 1.3E-08 |
|  | rs12907139 | -0.0149 | 0.0019 | -0.01169 | 61.49861 | 4.89E-15 |
|  | rs5742915 | 0.0248 | 0.0019 | 0.019449 | 170.3712 | 9.33E-39 |
|  | rs140657345 | -0.02 | 0.0022 | -0.01355 | 82.64463 | 5.53E-20 |
|  | rs373736365 | 0.0189 | 0.0022 | 0.012802 | 73.80372 | 4.53E-18 |
|  | rs11633371 | 0.0216 | 0.0019 | 0.01694 | 129.241 | 7.49E-30 |
|  | rs4932439 | -0.0151 | 0.0025 | -0.009 | 36.4816 | 1.43E-09 |
|  | rs2174008 | -0.0192 | 0.0019 | -0.01506 | 102.1163 | 5.89E-24 |
|  | rs577289 | -0.0125 | 0.0021 | -0.00887 | 35.43084 | 5.39E-09 |
|  | rs72726050 | -0.0192 | 0.0034 | -0.00842 | 31.88927 | 1.84E-08 |
|  | rs12909863 | 0.0189 | 0.0022 | 0.012802 | 73.80372 | 6.05E-18 |
|  | rs713467 | 0.0146 | 0.0019 | 0.011451 | 59.04709 | 3.09E-14 |
|  | rs11629593 | -0.0109 | 0.002 | -0.00812 | 29.7025 | 4.42E-08 |
|  | rs2663126 | -0.0139 | 0.0021 | -0.00986 | 43.81179 | 1.36E-11 |
|  | rs116092985 | -0.0401 | 0.0033 | -0.01811 | 147.6593 | 1.17E-34 |
|  | rs35811052 | -0.0148 | 0.0022 | -0.01003 | 45.2562 | 8.84E-12 |
|  | rs72771070 | 0.015 | 0.0021 | 0.010644 | 51.02041 | 1.3E-12 |
|  | rs62033029 | -0.0141 | 0.0023 | -0.00914 | 37.58223 | 1.73E-09 |
|  | rs72801843 | 0.0313 | 0.0021 | 0.022207 | 222.1519 | 8.83E-52 |
|  | rs55872725 | 0.0222 | 0.0019 | 0.01741 | 136.5208 | 1.46E-30 |
|  | rs4985445 | -0.0175 | 0.0019 | -0.01373 | 84.8338 | 3.3E-20 |
|  | rs17818592 | -0.0129 | 0.0019 | -0.01012 | 46.09695 | 1.23E-11 |
|  | rs8054549 | -0.0251 | 0.0019 | -0.01968 | 174.518 | 3.37E-39 |
|  | rs7185244 | -0.0148 | 0.0023 | -0.00959 | 41.40643 | 1.4E-10 |
|  | rs113478686 | -0.0244 | 0.0023 | -0.01581 | 112.5444 | 3.76E-27 |
|  | rs12051245 | 0.0299 | 0.0022 | 0.020251 | 184.7128 | 2.56E-40 |
|  | rs143076454 | -0.0499 | 0.007 | -0.01062 | 50.81653 | 1.06E-12 |
|  | rs246177 | 0.0214 | 0.002 | 0.015944 | 114.49 | 2.04E-27 |
|  | rs116008080 | -0.0415 | 0.0063 | -0.00982 | 43.39254 | 4.06E-11 |
|  | rs12926103 | 0.0272 | 0.0038 | 0.010667 | 51.23546 | 9.6E-13 |
|  | rs77364196 | -0.033 | 0.0043 | -0.01144 | 58.8967 | 8.35E-15 |
|  | rs61528919 | 0.014 | 0.002 | 0.010432 | 49 | 3.33E-12 |
|  | rs35816944 | -0.1088 | 0.0117 | -0.01386 | 86.4741 | 1.27E-20 |
|  | rs78457529 | -0.0904 | 0.0088 | -0.01531 | 105.5289 | 1.22E-24 |
|  | rs4788218 | 0.0275 | 0.0019 | 0.021565 | 209.4875 | 5.52E-46 |
|  | rs2240735 | 0.0189 | 0.0022 | 0.012802 | 73.80372 | 3.99E-18 |
|  | rs77809369 | 0.0237 | 0.0039 | 0.009056 | 36.92899 | 9.83E-10 |
|  | rs35268848 | 0.0737 | 0.0101 | 0.010874 | 53.24664 | 2.83E-13 |
|  | rs62070319 | -0.018 | 0.002 | -0.01341 | 81 | 2.54E-20 |
|  | rs6502935 | -0.0125 | 0.0022 | -0.00847 | 32.28306 | 6.31E-09 |
|  | rs113146332 | 0.0311 | 0.0049 | 0.009459 | 40.28363 | 2.99E-10 |
|  | rs9894577 | -0.031 | 0.002 | -0.02309 | 240.25 | 1.4E-52 |
|  | rs2005172 | 0.048 | 0.002 | 0.035745 | 576 | 2.3E-128 |
|  | rs28485212 | -0.0188 | 0.0027 | -0.01038 | 48.48285 | 1.24E-12 |
|  | rs9890062 | 0.0267 | 0.0039 | 0.010202 | 46.86982 | 1.2E-11 |
|  | rs117972846 | 0.0335 | 0.0057 | 0.008759 | 34.5414 | 5.47E-09 |
|  | rs2289629 | -0.0148 | 0.002 | -0.01103 | 54.76 | 8.02E-14 |
|  | rs2019203 | 0.0189 | 0.0019 | 0.014823 | 98.95014 | 1.84E-23 |
|  | rs9905385 | -0.0339 | 0.002 | -0.02525 | 287.3025 | 1.94E-63 |
|  | rs78766798 | 0.0319 | 0.0035 | 0.013582 | 83.0702 | 2.61E-20 |
|  | rs78378222 | 0.138 | 0.0087 | 0.023633 | 251.6052 | 4.51E-56 |
|  | rs2112617 | -0.0167 | 0.0019 | -0.0131 | 77.25485 | 1.07E-18 |
|  | rs2676298 | -0.0269 | 0.0027 | -0.01485 | 99.26063 | 2.39E-23 |
|  | rs7220127 | -0.0105 | 0.0019 | -0.00824 | 30.54017 | 4.6E-08 |
|  | rs36000545 | -0.022 | 0.002 | -0.01639 | 121 | 2.56E-29 |
|  | rs6505216 | -0.0498 | 0.0023 | -0.03225 | 468.8166 | 1.8E-101 |
|  | rs57791149 | -0.0173 | 0.0019 | -0.01357 | 82.90582 | 3.26E-19 |
|  | rs2521349 | 0.0155 | 0.0019 | 0.012157 | 66.55125 | 2.01E-15 |
|  | rs173135 | -0.0341 | 0.003 | -0.01694 | 129.2011 | 3.25E-30 |
|  | rs12943867 | 0.0184 | 0.002 | 0.01371 | 84.64 | 7.57E-20 |
|  | rs9898189 | -0.0163 | 0.0021 | -0.01157 | 60.24717 | 1.88E-15 |
|  | rs57513571 | -0.0191 | 0.0024 | -0.01186 | 63.33507 | 7.02E-16 |
|  | rs11867855 | -0.0132 | 0.0022 | -0.00894 | 36 | 2.93E-09 |
|  | rs4640244 | -0.02 | 0.0019 | -0.01569 | 110.8033 | 3.81E-25 |
|  | rs72829852 | 0.0309 | 0.0039 | 0.011807 | 62.77515 | 3.74E-15 |
|  | rs2592208 | -0.0124 | 0.0019 | -0.00973 | 42.5928 | 5.52E-11 |
|  | rs113232639 | 0.0327 | 0.0019 | 0.025641 | 296.2022 | 4.79E-64 |
|  | rs8084413 | -0.0127 | 0.0019 | -0.00996 | 44.67867 | 3.25E-11 |
|  | rs2978362 | 0.0106 | 0.0019 | 0.008314 | 31.12465 | 2.85E-08 |
|  | rs71336393 | 0.049 | 0.0023 | 0.031734 | 453.8752 | 9E-105 |
|  | rs74494415 | -0.0417 | 0.0049 | -0.01268 | 72.42357 | 1.82E-17 |
|  | rs2347808 | -0.0125 | 0.0019 | -0.0098 | 43.28255 | 5.79E-11 |
|  | rs33973388 | 0.0249 | 0.0019 | 0.019527 | 171.7479 | 1.45E-38 |
|  | rs4940874 | 0.0148 | 0.0024 | 0.00919 | 38.02778 | 1.24E-09 |
|  | rs1786263 | -0.019 | 0.0019 | -0.0149 | 100 | 1.03E-22 |
|  | rs62103240 | 0.0212 | 0.0037 | 0.008539 | 32.8298 | 1.4E-08 |
|  | rs4121583 | 0.0118 | 0.002 | 0.008793 | 34.81 | 4.62E-09 |
|  | rs568267 | 0.0122 | 0.0022 | 0.008264 | 30.75207 | 2.23E-08 |
|  | rs35073631 | 0.0112 | 0.0019 | 0.008785 | 34.74792 | 5.92E-09 |
|  | rs12962050 | 0.0153 | 0.002 | 0.0114 | 58.5225 | 1.52E-14 |
|  | rs7229520 | -0.0224 | 0.002 | -0.01669 | 125.44 | 9.21E-29 |
|  | rs7228151 | -0.0185 | 0.0023 | -0.01199 | 64.69754 | 3.19E-15 |
|  | rs9957318 | 0.0187 | 0.002 | 0.013933 | 87.4225 | 1.02E-20 |
|  | rs151123488 | 0.0178 | 0.0029 | 0.009147 | 37.6742 | 5.07E-10 |
|  | rs60389750 | -0.0175 | 0.0021 | -0.01242 | 69.44444 | 1.06E-16 |
|  | rs79441499 | -0.0138 | 0.0019 | -0.01082 | 52.75346 | 9.67E-13 |
|  | rs11373507 | -0.0113 | 0.002 | -0.00842 | 31.9225 | 1.12E-08 |
|  | rs45474992 | -0.0617 | 0.0051 | -0.01803 | 146.3626 | 2.17E-33 |
|  | rs10421750 | -0.0145 | 0.0021 | -0.01029 | 47.67574 | 5.73E-12 |
|  | rs16989695 | -0.0139 | 0.0019 | -0.0109 | 53.52078 | 1.93E-13 |
|  | rs12150907 | -0.0219 | 0.0024 | -0.0136 | 83.26563 | 7.22E-20 |
|  | rs10948 | -0.0252 | 0.002 | -0.01877 | 158.76 | 3.43E-36 |
|  | rs2607234 | -0.0302 | 0.0043 | -0.01047 | 49.32612 | 2E-12 |
|  | rs117203652 | -0.0346 | 0.0055 | -0.00938 | 39.57554 | 4.32E-10 |
|  | rs4807472 | -0.0158 | 0.002 | -0.01177 | 62.41 | 8.18E-15 |
|  | rs12461874 | -0.0181 | 0.0021 | -0.01284 | 74.28798 | 1.26E-17 |
|  | rs116919274 | 0.0271 | 0.0046 | 0.00878 | 34.70747 | 3.52E-09 |
|  | rs111901094 | -0.0253 | 0.0025 | -0.01508 | 102.4144 | 4.04E-24 |
|  | rs75702986 | -0.0163 | 0.0025 | -0.00972 | 42.5104 | 3.14E-11 |
|  | rs4252548 | -0.0753 | 0.0065 | -0.01726 | 134.2033 | 2.96E-31 |
|  | rs8112948 | -0.0297 | 0.0022 | -0.02012 | 182.25 | 4.24E-42 |
|  | rs350832 | -0.0165 | 0.0023 | -0.01069 | 51.46503 | 3.44E-13 |
|  | rs11260035 | 0.015 | 0.0021 | 0.010644 | 51.02041 | 1.86E-12 |
|  | rs11672848 | -0.0171 | 0.0019 | -0.01341 | 81 | 7.73E-19 |
|  | rs2287821 | -0.0153 | 0.0019 | -0.012 | 64.84488 | 6.95E-16 |
|  | rs147110934 | -0.0722 | 0.0062 | -0.01735 | 135.6098 | 9.39E-32 |
|  | rs6054390 | -0.0188 | 0.002 | -0.01401 | 88.36 | 1.45E-21 |
|  | rs73125634 | -0.0195 | 0.0021 | -0.01384 | 86.22449 | 5.11E-20 |
|  | rs12185775 | -0.0167 | 0.003 | -0.0083 | 30.98778 | 3.66E-08 |
|  | rs112021215 | -0.0147 | 0.0025 | -0.00876 | 34.5744 | 5.99E-09 |
|  | rs4815952 | -0.0161 | 0.0019 | -0.01263 | 71.80332 | 1.24E-16 |
|  | rs35963161 | -0.0157 | 0.0019 | -0.01231 | 68.27978 | 7.46E-16 |
|  | rs77447813 | 0.0224 | 0.0034 | 0.009818 | 43.40484 | 3.21E-11 |
|  | rs2236096 | 0.018 | 0.0023 | 0.011663 | 61.24764 | 1.29E-15 |
|  | rs6142059 | 0.0116 | 0.0019 | 0.009098 | 37.27424 | 1.19E-09 |
|  | rs143384 | 0.0725 | 0.0019 | 0.056775 | 1456.025 | 1E-200 |
|  | rs80132799 | 0.0231 | 0.0038 | 0.009059 | 36.9536 | 1.31E-09 |
|  | rs6054491 | -0.0142 | 0.0022 | -0.00962 | 41.66116 | 2.15E-10 |
|  | rs684905 | -0.0118 | 0.0019 | -0.00926 | 38.57064 | 7.1E-10 |
|  | rs6082354 | -0.024 | 0.002 | -0.01788 | 144 | 1.19E-32 |
|  | rs4287835 | 0.0147 | 0.0019 | 0.01153 | 59.85873 | 9.94E-15 |
|  | rs1291114 | 0.0173 | 0.0031 | 0.008317 | 31.1436 | 1.95E-08 |
|  | rs57696574 | 0.0173 | 0.002 | 0.01289 | 74.8225 | 4.5E-18 |
|  | rs34879158 | -0.0363 | 0.0022 | -0.02458 | 272.25 | 1.55E-63 |
|  | rs6028716 | -0.021 | 0.0022 | -0.01422 | 91.1157 | 4.58E-22 |
|  | rs6066122 | 0.0127 | 0.0023 | 0.008229 | 30.4896 | 1.76E-08 |
|  | rs13037813 | 0.0292 | 0.0022 | 0.019777 | 176.1653 | 1.71E-39 |
|  | rs73197345 | 0.0211 | 0.0028 | 0.01123 | 56.78699 | 3.55E-14 |
|  | rs12483401 | -0.0387 | 0.0067 | -0.00861 | 33.36356 | 9.22E-09 |
|  | rs2230033 | -0.0265 | 0.0019 | -0.02078 | 194.5291 | 3.49E-43 |
|  | rs112153300 | 0.0261 | 0.0034 | 0.01144 | 58.9282 | 7.05E-15 |
|  | rs35631698 | 0.0145 | 0.002 | 0.010804 | 52.5625 | 2.59E-13 |
|  | rs2212926 | -0.022 | 0.0023 | -0.01425 | 91.49338 | 7.75E-21 |
|  | rs4818280 | -0.0124 | 0.002 | -0.00924 | 38.44 | 2.84E-10 |
|  | rs9610447 | 0.0152 | 0.0022 | 0.010296 | 47.73554 | 5.29E-12 |
|  | rs5753518 | 0.0242 | 0.0033 | 0.010928 | 53.77778 | 5.04E-13 |
|  | rs7286917 | 0.0171 | 0.0023 | 0.011079 | 55.27599 | 5.18E-14 |
|  | rs41311445 | -0.0328 | 0.0032 | -0.01527 | 105.0625 | 4.73E-24 |
|  | rs5763821 | 0.0191 | 0.002 | 0.014231 | 91.2025 | 1.06E-21 |
|  | rs6000886 | 0.0131 | 0.002 | 0.009761 | 42.9025 | 5.88E-11 |
|  | rs8136517 | 0.0267 | 0.0039 | 0.010202 | 46.86982 | 6.58E-12 |
|  | rs165849 | 0.0157 | 0.0021 | 0.011141 | 55.89342 | 4.57E-14 |
|  | rs10453441 | -0.0139 | 0.002 | -0.01036 | 48.3025 | 9.1E-13 |
|  | rs28379706 | 0.0114 | 0.002 | 0.008494 | 32.49 | 4.49E-09 |
| Usual walking pace |  |  |  |  |  |  |
|  | rs12739999 | -0.01353 | 0.001975 | -0.01183 | 46.9193 | 7.41E-12 |
|  | rs75854315 | -0.01713 | 0.003007 | -0.00983 | 32.43972 | 1.23E-08 |
|  | rs7560257 | -0.01149 | 0.002065 | -0.00961 | 30.95655 | 2.64E-08 |
|  | rs61134960 | 0.010127 | 0.001799 | 0.00972 | 31.68746 | 1.81E-08 |
|  | rs5026760 | 0.011317 | 0.002036 | 0.009599 | 30.9043 | 2.71E-08 |
|  | rs2054079 | 0.009792 | 0.00159 | 0.010635 | 37.93159 | 7.33E-10 |
|  | rs10865958 | -0.00992 | 0.001483 | -0.01154 | 44.69082 | 2.31E-11 |
|  | rs9844666 | -0.00965 | 0.001739 | -0.00958 | 30.80451 | 2.86E-08 |
|  | rs13107325 | -0.02596 | 0.002824 | -0.01587 | 84.47089 | 3.92E-20 |
|  | rs9637592 | -0.0091 | 0.0016 | -0.00982 | 32.31833 | 1.31E-08 |
|  | rs9379843 | 0.008685 | 0.001482 | 0.010118 | 34.33125 | 4.65E-09 |
|  | rs4839898 | 0.014075 | 0.002414 | 0.010069 | 34.00068 | 5.51E-09 |
|  | rs9471333 | 0.008604 | 0.00149 | 0.009968 | 33.32636 | 7.8E-09 |
|  | rs11152989 | -0.00954 | 0.001599 | -0.0103 | 35.59028 | 2.44E-09 |
|  | rs4509216 | -0.00844 | 0.001484 | -0.00983 | 32.38501 | 1.27E-08 |
|  | rs9791848 | -0.00916 | 0.001672 | -0.00946 | 30.02259 | 4.27E-08 |
|  | rs13238384 | 0.010212 | 0.001541 | 0.011444 | 43.92677 | 3.41E-11 |
|  | rs7896518 | 0.009012 | 0.001516 | 0.010263 | 35.32523 | 2.79E-09 |
|  | rs10828258 | -0.00983 | 0.001594 | -0.01065 | 38.04445 | 6.92E-10 |
|  | rs7124681 | -0.01009 | 0.001507 | -0.01156 | 44.83279 | 2.15E-11 |
|  | rs10750025 | -0.00895 | 0.001602 | -0.00965 | 31.21686 | 2.31E-08 |
|  | rs10862220 | 0.010301 | 0.001589 | 0.011194 | 42.0295 | 9E-11 |
|  | rs10149134 | -0.00941 | 0.00152 | -0.01069 | 38.34778 | 5.93E-10 |
|  | rs7140836 | -0.00871 | 0.001501 | -0.01002 | 33.6762 | 6.51E-09 |
|  | rs4780421 | 0.008904 | 0.001532 | 0.010034 | 33.76503 | 6.22E-09 |
|  | rs34898535 | 0.008425 | 0.001533 | 0.009492 | 30.22 | 3.86E-08 |
|  | rs9972653 | -0.01029 | 0.001519 | -0.0117 | 45.91454 | 1.24E-11 |
|  | rs1652376 | 0.008937 | 0.001488 | 0.010373 | 36.08709 | 1.89E-09 |
|  | rs784257 | -0.01401 | 0.001911 | -0.01266 | 53.72973 | 2.31E-13 |
|  | rs273505 | -0.00999 | 0.001504 | -0.01148 | 44.16884 | 3.02E-11 |
| KOA |  |  |  |  |  |  |
|  | rs17567417 | -0.0655 | 0.0093 | -0.01109 | 49.604 | 1.96E-12 |
|  | rs12470967 | -0.0584 | 0.0103 | -0.00893 | 32.1478 | 1.5E-08 |
|  | rs9277552 | -0.064 | 0.0114 | -0.00884 | 31.51739 | 1.97E-08 |
|  | rs1078301 | 0.0679 | 0.0106 | 0.010088 | 41.03248 | 1.27E-10 |
|  | rs56116847 | 0.0612 | 0.0097 | 0.009937 | 39.80699 | 3.19E-10 |
|  | rs4775006 | 0.0578 | 0.0094 | 0.009684 | 37.80942 | 8.4E-10 |
|  | rs6499244 | 0.0622 | 0.0094 | 0.010421 | 43.78497 | 3.88E-11 |
|  | rs35087650 | 0.0694 | 0.0114 | 0.009588 | 37.06033 | 1.18E-09 |
|  | rs8067763 | -0.0566 | 0.0095 | -0.00938 | 35.49651 | 2.39E-09 |
|  | rs143384 | -0.0935 | 0.0095 | -0.0155 | 96.86704 | 4.77E-23 |
| HOA |  |  |  |  |  |  |
|  | rs12040949 | -0.0665 | 0.012 | -0.00883 | 30.71007 | 2.83E-08 |
|  | rs11583641 | -0.0811 | 0.0131 | -0.00986 | 38.3265 | 5.57E-10 |
|  | rs4338381 | -0.095 | 0.0121 | -0.01251 | 61.64196 | 4.37E-15 |
|  | rs74767794 | -0.0751 | 0.0126 | -0.0095 | 35.52538 | 2.56E-09 |
|  | rs2785988 | 0.0828 | 0.0127 | 0.010388 | 42.50629 | 7.3E-11 |
|  | rs7571789 | -0.0886 | 0.0117 | -0.01207 | 57.34502 | 3.26E-14 |
|  | rs1835323 | -0.0673 | 0.0123 | -0.00872 | 29.9378 | 4.56E-08 |
|  | rs3774355 | 0.0907 | 0.0121 | 0.011943 | 56.18803 | 8.2E-14 |
|  | rs798748 | 0.0715 | 0.012 | 0.009494 | 35.50174 | 2.5E-09 |
|  | rs1913707 | -0.0795 | 0.012 | -0.01056 | 43.89063 | 2.96E-11 |
|  | rs12209223 | 0.1558 | 0.0191 | 0.012996 | 66.53776 | 3.88E-16 |
|  | rs2396502 | 0.0842 | 0.012 | 0.01118 | 49.23361 | 2.12E-12 |
|  | rs115740542 | 0.1263 | 0.0224 | 0.008984 | 31.79147 | 1.6E-08 |
|  | rs80287694 | 0.1093 | 0.0184 | 0.009465 | 35.28618 | 2.66E-09 |
|  | rs60890741 | -0.1087 | 0.0185 | -0.00936 | 34.52356 | 4.5E-09 |
|  | rs2929451 | -0.0691 | 0.0117 | -0.00941 | 34.88063 | 3.11E-09 |
|  | rs13300602 | 0.0716 | 0.0119 | 0.009587 | 36.20196 | 1.65E-09 |
|  | rs34687269 | -0.0826 | 0.0117 | -0.01125 | 49.84119 | 1.67E-12 |
|  | rs10896015 | -0.0782 | 0.0132 | -0.00944 | 35.09665 | 2.74E-09 |
|  | rs10492367 | 0.1518 | 0.0148 | 0.016341 | 105.2011 | 1.25E-24 |
|  | rs79056043 | 0.1625 | 0.0268 | 0.009661 | 36.76522 | 1.33E-09 |
|  | rs11059094 | 0.0759 | 0.0117 | 0.010336 | 42.0835 | 7.38E-11 |
|  | rs12901372 | -0.0783 | 0.0118 | -0.01057 | 44.0311 | 3.46E-11 |
|  | rs62063281 | 0.0964 | 0.014 | 0.010971 | 47.41306 | 5.3E-12 |
|  | rs7222178 | 0.0965 | 0.0146 | 0.010531 | 43.68667 | 3.77E-11 |
|  | rs4252548 | 0.2785 | 0.0396 | 0.011205 | 49.46067 | 1.96E-12 |
|  | rs2836618 | 0.0876 | 0.0132 | 0.010574 | 44.04132 | 3.2E-11 |
| KOA/HOA |  |  |  |  |  |  |
|  | rs2622873 | -0.0684 | 0.0113 | -0.00937 | 36.63999 | 1.58E-09 |
|  | rs2820443 | 0.0543 | 0.0083 | 0.010123 | 42.79997 | 6.01E-11 |
|  | rs4630744 | -0.0538 | 0.0076 | -0.01095 | 50.1115 | 2.1E-12 |
|  | rs3821262 | -0.0554 | 0.0076 | -0.01128 | 53.13643 | 3.52E-13 |
|  | rs12470967 | -0.048 | 0.0085 | -0.00874 | 31.88927 | 1.53E-08 |
|  | rs3774354 | 0.0536 | 0.0079 | 0.010499 | 46.03365 | 1.37E-11 |
|  | rs11923760 | -0.0448 | 0.0082 | -0.00845 | 29.8489 | 4.16E-08 |
|  | rs11732213 | -0.0588 | 0.0096 | -0.00948 | 37.51563 | 8.81E-10 |
|  | rs3884606 | -0.0437 | 0.0076 | -0.0089 | 33.0625 | 8.25E-09 |
|  | rs9277552 | -0.0592 | 0.0093 | -0.00985 | 40.52075 | 2.37E-10 |
|  | rs10948196 | 0.0426 | 0.0078 | 0.008451 | 29.8284 | 4.5E-08 |
|  | rs2299285 | 0.0463 | 0.008 | 0.008956 | 33.49516 | 7.57E-09 |
|  | rs11997261 | -0.0542 | 0.0087 | -0.00964 | 38.81147 | 5.16E-10 |
|  | rs4979341 | 0.0597 | 0.0086 | 0.010742 | 48.18943 | 3.35E-12 |
|  | rs10758594 | 0.0436 | 0.0077 | 0.008762 | 32.06207 | 1.69E-08 |
|  | rs17659798 | -0.0539 | 0.0085 | -0.00981 | 40.21052 | 2.06E-10 |
|  | rs7935877 | -0.0822 | 0.0149 | -0.00854 | 30.43485 | 3.41E-08 |
|  | rs10492367 | 0.0545 | 0.0097 | 0.008694 | 31.56818 | 1.96E-08 |
|  | rs4144502 | 0.0468 | 0.0076 | 0.009529 | 37.91967 | 9.48E-10 |
|  | rs56116847 | 0.0453 | 0.008 | 0.008762 | 32.06391 | 1.28E-08 |
|  | rs2472304 | 0.0452 | 0.0081 | 0.008635 | 31.13916 | 2.03E-08 |
|  | rs9930333 | 0.0464 | 0.0077 | 0.009325 | 36.31236 | 1.51E-09 |
|  | rs2953013 | -0.0524 | 0.0083 | -0.00977 | 39.85716 | 3.07E-10 |
|  | rs75621460 | 0.1523 | 0.0256 | 0.009206 | 35.3932 | 2.88E-09 |
|  | rs143384 | -0.0634 | 0.0077 | -0.01274 | 67.79491 | 2.42E-16 |
|  | rs9977881 | 0.0607 | 0.0102 | 0.009209 | 35.41417 | 2.54E-09 |
